# Amino-BODIPY as the ratiometric fluorescent sensor for monitoring drug release or “power supply” selector for molecular electronics†

**DOI:** 10.1039/c9ra03472b

**Published:** 2019-08-13

**Authors:** Martin Porubský, Soňa Gurská, Jarmila Stanková, Marián Hajdúch, Petr Džubák, Jan Hlaváč

**Affiliations:** Department of Organic Chemistry, Faculty of Science, Palacký University Tr. 17. Listopadu 12 771 46 Olomouc Czech Republic jan.hlavac@upol.cz; Institute of Molecular and Translational Medicine, Faculty of Medicine and Dentistry, Palacký University Hněvotínská 5 779 00 Olomouc Czech Republic

## Abstract

The glutathione cleavable conjugates of amino-BODIPY dye with model drugs have been tested for monitoring the drug release *via* ratiometric fluorescence based on two excitation and one emission wavelength. As a self-immolative linker was used for the construction of conjugates, free amino-BODIPY was released with the drug. Different excitation profiles of the dye before and after conjugate cleavage and similar emission wavelengths that enabled monitoring the release of the drug *via* the OFF–ON effect were successfully tested inside the cancer cells. UV/Vis spectrometry could be used in the quantification of the conjugate/drug in an analyte irrespective of the cleavage grade. As the system functionality was based only on the altered acylamino-BODIPY present in the conjugate to amino-BODIPY released during the cleavage, the method could be applied as a ratiometric fluorescence theranostic system to other non-fluorescent drugs. Moreover, the present conjugates demonstrated their potential application in molecular electronics as a “power supply” selector enabling the application of two power sources for one “bulb” to maintain its light intensity.

## Abstract 2

Amino-BODIPY as the universal and highly fluorescent OFF–ON and ratiometric sensor for thiol-mediated drug release monitoring.

## Graphical abstract

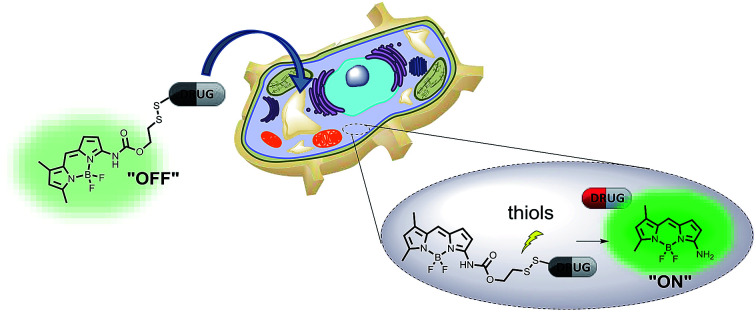

## Introduction

The ability to monitor a drug's fate, including its conjugate penetration and the subsequent drug release is one of the key features in the development of new drug delivery systems (DDS) in cancer therapy.^1^ The detection of relevant markers along with monitoring the drug release with time ranks these systems among the most intensively studied theranostics.^2^

The most frequently used visualization method for drug release is optical fluorescent spectroscopy, using various dyes such as cyanines, xanthene dyes, coumarines, *etc.* The frequently-used BODIPY dyes with total neutral charge, hydrophobic nature, and adjustable photochemical properties seem to be the first candidates of choice for penetration-visualization studies. In addition, BODIPYs are highly photostable and possess high quantum yields, high extinction coefficients, and sharp excitation/emission spectra.^3^

Optical imaging often utilizes activatable probes that effectuate amplified signal in the presence of selective or overexpressed biomarker or due to specific molecular events. This phenomenon is fundamental for the OFF–ON or ON–OFF systems used in several diagnostic applications.^4–10^ The greatest advantage of the fluorescent systems applied in chemical biology is the ratiometric measurement,^11–13^ when two emission maxima are reached after excitation at one wavelength or when two excitation wavelengths cause one emission peak. Ratiometric systems easily overcome some drawbacks of simple intensiometric systems, especially false response caused by variation in the local concentration of the probe, light scattering by the sample matrix, excitation source fluctuation, or microenvironment effects around the probe.

Thiols, mainly glutathione (GSH), responsible for different redox state of the cancer cells,^14^ are the key intracellular stimuli for the release of a drug from DDS or theranostics. Consequently, the level of glutathione in some tumors is up to 10-fold higher as compared to normal cells.^15^ Elevated GSH level is typical of some cancer cells,^16^ which makes them ideal triggers for the release of a drug from systems including disulfide linker.

Although a few disulfide theranostic prodrugs with an ability to monitor drug releasing *via* fluorescence have been introduced in recent years,^17–22^ only a few were reported to have the ratiometric ability for monitoring the drug release.^23,24^ These ratiometric OFF–ON systems are responsive to the presence of GSH and are based on one excitation/two emissions. However, the drawback of these systems is the application of the fluorescence resonance energy transfer (FRET) between camptothecin as the drug and the fluorescent dye. Thus, this system lacks the general application to drugs without regards to their fluorescent properties.

In the molecular electronics, the OFF–ON or ON–OFF concept could be used for the construction of molecular logic gates operated by various inputs, such as pH, the presence of metal ions, or other specific markers.^25^ Although the molecular switches based on the (ir)reversible turn-on or turn-off effect giving the fluorescence response in the presence of appropriate marker has been described several times,^26^ a selector as a molecular electronic device that can switch two different power supplies for one appliance while maintaining the level of power has not yet been described.

In this study, we report a new acylamino-BODIPY/amino-BODIPY system applicable in chemical biology or molecular electronics. As the specific spectral properties of the amino-BODIPY are exhibited together with the drug after conjugate cleavage, the drug release can be monitored *via* OFF–ON effect as well as ratiometric fluorescence irrespective of the fluorescent properties of the drug. The system could also serve as a molecular electronic selector activated by the presence of thiols and optimal pH.

## Experimental section

### Materials and methods

For the preparation and characterization of the compounds, LC/MS analyses were performed using UHPLC/MS with an UHPLC chromatograph (Acquity) with a PDA detector, a single quadrupole mass spectrometer (Waters), an X-Select C18 column at 30 °C and a flow rate of 600 μl min^−1^. The mobile phase consisted of (A) 0.01 M ammonium acetate in water and (B) acetonitrile, with a linear gradient over the course of 2.5 min; at the conclusion of the gradient, the final ratio was maintained for 1.5 min. The column was re-equilibrated with 10% B for 1 min. The APCI ionization operated at a discharge current of 5 μA, a vaporizer temperature of 350 °C and a capillary temperature of 200 °C. Compound purity was determined using the ratio of the appropriate peak area to sum of areas of all peaks of the mixture. Areas were determined by integration of the peaks from the PDA detector response. Purity of final compounds was determined by this method and was >95%.

Purification was performed using semi-preparative HPLC with a Waters 1500 series HPLC equipped with a 2707 Autosampler, a 1525 binary HPLC pump, a 2998 Waters Photodiode Array Detector and a Waters Fraction Collector III with a YMC C18 reverse phase column (20 × 100 mm, 5 μm particle size). The mobile phase consisted of acetonitrile and a 10 mM aqueous ammonium acetate gradient over 6 min.

NMR spectra were measured in CDCl_3_ or DMSO-d_6_ using a Jeol ECX-500 (500 MHz) spectrometer. Chemical shifts (*δ*) are reported in parts per million (ppm), and coupling constants (*J*) are reported in Hertz (Hz).

HR-MS analysis was performed using an Orbitrap Elite high-resolution mass spectrometer (Thermo Fischer Scientific, MA, USA) operating at positive full scan mode (120 000 FWMH) in the range of 2000–3000 *m*/*z*. The settings for electrospray ionization were as follows: oven temperature of 300 °C, sheath gas of 8 arb. units and source voltage of 1.5 kV. Samples were diluted to a final concentration of 20 μmol l^−1^ with 0.1% formic acid in water and methanol (50 : 50, v/v).

Rink amide resin and Fmoc-amino acids were purchased from AAPPTec (Louisville, KY). Solvents and other chemicals were purchased from Sigma-Aldrich (Milwaukee, IL, http://www.sigmaaldrich.com).

Fluorescence spectra were recorded on a Varian Cary Eclipse fluorescence spectrophotometer equipped with a thermostat (FL1009M015). Excitation and emission slits were 5 nm. Absorption spectra were recorded on a Cary 300 UV/Vis spectrophotometer (UV111M031, Agilent). Excitation spectra of the model drugs 1–3 correspond to general observations published previously^27,28^ and don't interfere with the spectra of the conjugates 4–6 or released dye 7.

### Quantum yield determination

Quantum yields (*Φ*) were calculated by standard procedure using Fluorescein as a reference (*Φ* = 0.91) according to eqn (1).

1 *Φ* = *Φ*_R_ × *I*/*I*_R_ × *A*_R_/*A* × *η*^2^/(*η*_R_^2^)

where *Φ*_R_ is the quantum yield of reference fluorophore, *I* is area under emission peak, *A* is absorbance at the excitation wavelength *η* is refractive index of the solvent.

### Cleavage of the conjugates 4–6 by glutathione and its LC/MS monitoring

0.25 ml of the conjugate 4, 5 and 6 solution (2 mM) in DMSO was mixed with 0.1 ml of GSH solution (50 mM) in HEPES (0.1 M, pH 7.4) and diluted with 0.65 ml DMSO/HEPES (2 : 1 v/v). The mixture was heated to 37 °C and analyzed by LC/MS within the time. Intracellular cleavage of conjugates 4–6 by glutathione and its fluorescence monitoring.

### Cleavage of the conjugates 4–6 by glutathione and its fluorescence monitoring

5 μl of the probe 4, 5 or 6 solution (1 mM) in DMSO was mixed with 20 μl, 60 μl or 100 μl of the GSH solution (50 mM) in HEPES buffer (0.1 M; pH 7.4) and diluted by HEPES buffer (0.1 M; pH 7.4) or DMSO/HEPES buffer (2 : 1) to 1 ml. The mixture was heated to 37 °C and the fluorescence was measured within the time.

### Intracellular cleavage of conjugates 4–6 by glutathione and its fluorescence monitoring

HeLa cells were added to black 96-well plates by MultiDrop Combi (Thermo Fisher Scientific, USA) at a cell density of 1.25 × 10^4^ per well and incubated overnight. Pre-treatment with GSH was performed by incubation of cells with GSH (20 mM in medium) for 2 h. The cells were washed with PBS, immediately treated with tested compounds for 2 min and washed with PBS again. Finally, 50 μl of PBS was added to each well. Fluorescence intensity was measured by EnVision plate reader (PerkinElmer, USA), two reads for each time point (first with ex 510 nm/em 535 nm and second with ex 485 nm/em 535 nm).

### Fluorescent microscopy imaging

HeLa cells (3000 per well, 30 μl per well) were seeded into 384 CellCarrier plates (PerkinElmer, USA) for live cell fluorescence imaging and preincubated for 24 h at 37 °C and 5% CO_2_ to adhere. The cells were pretreated with 20 mM GSH for 2 h and stained by Hoechst dye in concentration 1.62 μM within last 20 min. Further, the stained cells were rinsed with fresh media, treated with tested compounds (10 μM) for 2 min and rinsed with fresh media again. The live-cell imaging was performed by Cell Voyager CV7000 (Yokogawa, Japan) spinning disc confocal microscopy system at 37 °C in 5% CO_2_ atmosphere. Living cells were monitored by a 40× water immersion objective. All images were post-processed, background subtracted and deconvolved using Image J software.

## Results and discussion

**Scheme 1:**
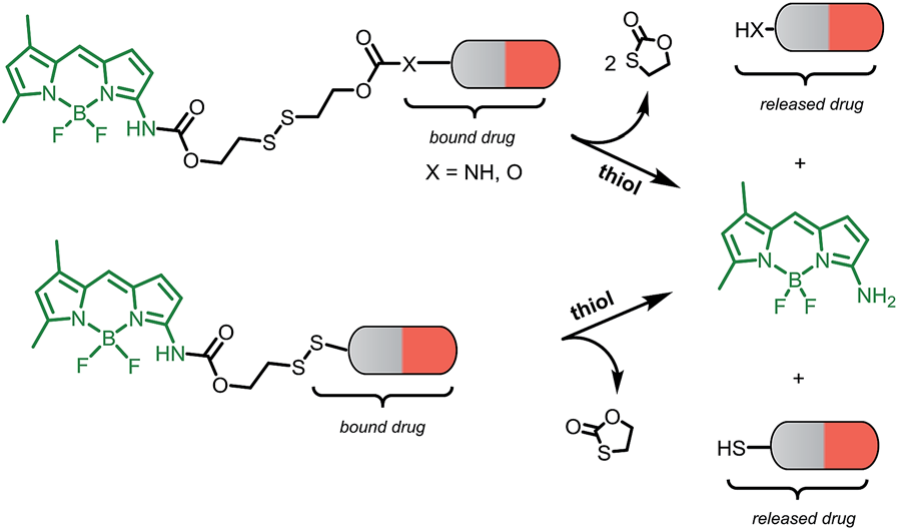
Schematic illustration of the drug and amino-BODIPY release from their conjugates.

This novel system is suggested as the conjugate of amino-BODIPY dye acylated by (a) symmetrical self-immolative disulfide linker, connected to a drug predestinated to release. This system was designed based on the previous study by Jain *et al.*^29^ The symmetrical linker can be used for binding of a drug with an amino or hydroxy group, whereas the asymmetrical linker can be used for binding of a drug *via* its thiol group (Scheme 1). The disulfide bridge provides a switch that triggers the release of the drug and free amino-BODIPY in the presence of thiols.

**Fig. 1:**
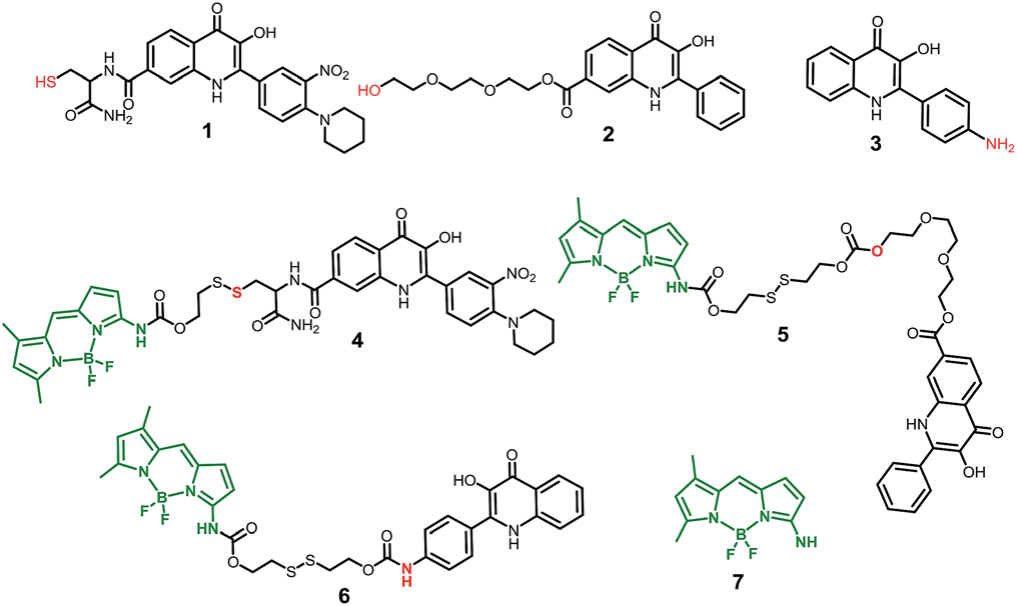
Structure of model drugs 1, 2, and 3 (ref. 18) and their conjugates 4, 5, and 6 used for thiol-mediated cleavage studies.

To study the possibility of monitoring a drug release, we used the compounds 1–3 from a group of 2-phenyl-3-hydroxy-4(1*H*)-quinolinone derivatives, known for their anticancer activity^30^ as the model drugs. These drugs are substituted by the thiol, hydroxy, or amino group, suitable for conjugation with amino-BODIPY 7. The structure of these model drugs and their conjugates 4–6 are presented in Fig. 1.

**Scheme 2:**
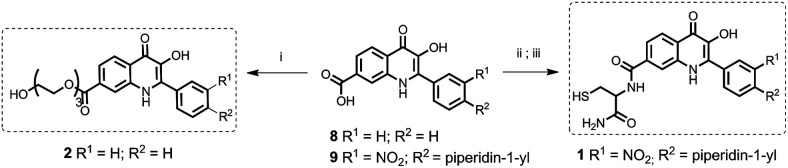
Synthesis of model drugs 1 and 2. (i) 8, triethyleneglycol, H_2_SO_4_ (cat.), THF, reflux, on; (ii) Rink amide resin preloaded with cysteine, 9, HOBt, DIC, DMF/Pyr, 3 h, rt. (iii) TFA/DCM/TES (2 : 1 : 0.05), 1 h, rt.

The model drug 1 was synthesized from quinolinone 8 (ref. 30) by standard peptide synthesis with immobilized Fmoc-cysteine on Rink resin. Compound 2 was prepared by esterification of the starting derivative 9 with triethyleneglycol (Scheme 2). Compound 3 was synthesized according to the previously published procedure.^30^

**Scheme 3:**
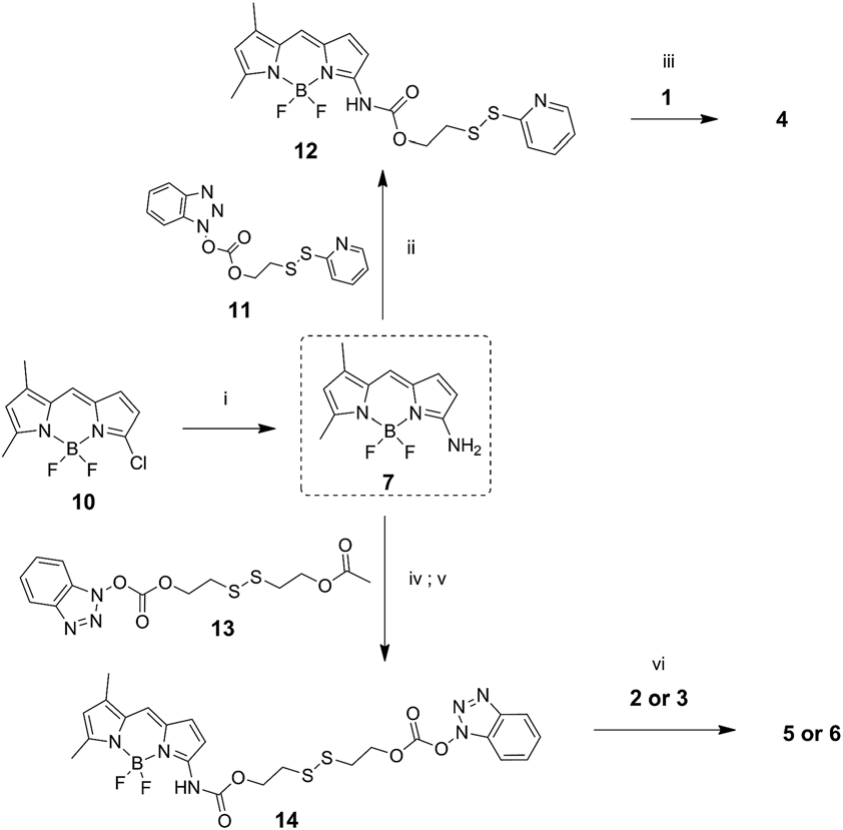
Synthesis of amino-BODIPY 7 and its conjugates 4–6. (i) 7.0 M NH_3_/MeOH, DCM 70 °C, on; (ii) DMAP, TEA, DCM, rt, 3 h; (iii) DMF, 60 °C, on; (iv) TEA, DMAP, 13, DCM, rt, 3 h. (v) K_2_CO_3_, THF/MeOH 3 : 1, rt, on; (vi) DMAP, TEA, DCM, rt, on.

Amino-BODIPY 7 was prepared by reaction of previously published chloroderivative 10 (ref. 31) with ammonia in methanol/DCM as described previously. The reaction with disulfide precursor 11 (ref. 32) effectuated the intermediate 12. The disulfide exchange of this intermediate with mercapto derivative 1 afforded the final disulfide conjugate 4 (Scheme 3).

The coupling of amino-BODIPY 7 with linker precursor 13 and subsequent reaction with HOBt gave rise to compound 14 with activated carbonate group. Then, final conjugates 5 and 6 were prepared by coupling the derivative 14 with the corresponding quinolinones 2 or 3 respectively *via* carbamate or carbonate bond (Scheme 3).

The fluorescence spectra of amino-BODIPY 7 and its conjugates 4–6 were first measured in HEPES buffer (0.1 M, pH 7.4) to meet the requirements for the planned biological experiments.

**Fig. 2:**
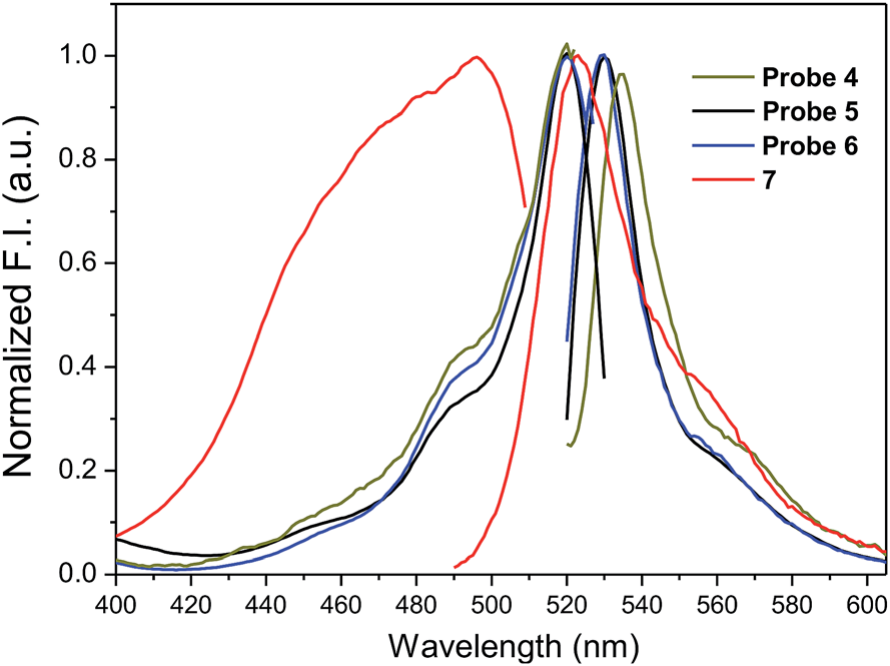
Normalized fluorescence excitation and emission spectra of amino-BODIPY 7 and its conjugates 4–6 (HEPES buffer, 0.1 M, pH 7.4).

Amino-BODIPY 7 has one broad excitation maximum at 480 nm and emission at 523 nm (Table 1; Fig. 2). Acylamino-BODIPY in conjugates 4–6 possesses excitation maxima 515–517 nm, while emission of 525–527 nm is very close to the emission of amino-BODIPY 7 (Fig. 2; Table 1).

**Table 1:** Fluorescence profile of conjugates 4–6, 18, 19 and BODIPY dyes 7, 20

Compound	Solvent	*λ*_exc_ (nm)	*λ*_em_ (nm)	Δ*λ* (nm)	QY (%)
4	HEPES	515	527	12	1.4
	DMSO/HEPES	517	531	14	7.7
5	HEPES	516	525	9	14
	DMSO/HEPES	520	530	10	58
6	HEPES	517	525	8	32
	DMSO/HEPES	521	532	11	53
7	HEPES	480	523	43	77
	DMSO/HEPES	496	523	40	95
18	DMSO/HEPES	521	549	28	23
19	DMSO/HEPES	520	531	11	95
20	DMSO/HEPES	521	550	29	48

**Fig. 3:**
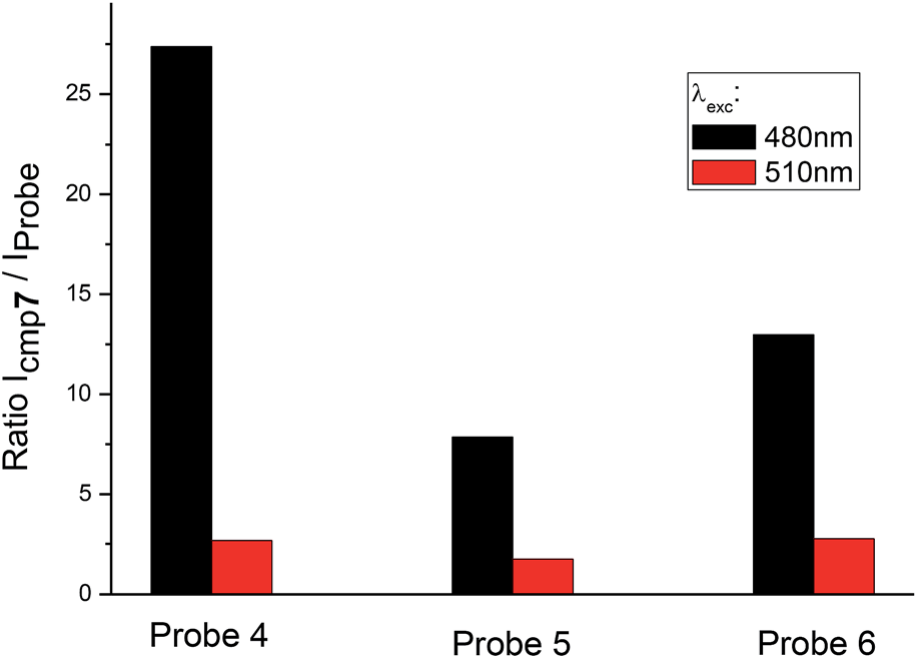
Ratio of fluorescence intensity of amino-BODIPY 7 to conjugates 4–6 at 525 nm after excitation at 480 nm or 510 nm (HEPES buffer, 0.1 M, pH 7.4).

Besides the shift of excitation maxima, a significant difference was observed between amino-BODIPY 7 and its conjugates 4–6 with respect to the quantum yields and fluorescence intensity. When excitation wavelength at 480 nm, which is the characteristic maximum for amino-BODIPY 7, was used and emission at 525 nm was collected, a significant difference was observed between the fluorescence intensity of amino-BODIPY 7 and its conjugates 4–6 (Fig. 3), and thus, the system can work as OFF–ON during the cleavage. The intensity ratio of amino-BODIPY 7 to appropriate conjugate 4–6 differs probably due to different quenching effect of the bound derivatives 1–3. Therefore a significant difference was observed between amino-BODIPY 7 and conjugate 4, in which the fluorescence is expectedly quenched by the nitro group.

When the excitation wavelength was selected at 510 nm, what is close to the excitation maxima of conjugates 4–6 and sufficiently distal from the emission maximum of BODIPY 7, the emission intensity was high for amino-BODIPY 7 due to its broad excitation spectrum and high quantum yield (Fig. 3; Table 1). The system could work as OFF–ON during the cleavage as well, but less efficiently.

**Fig. 4:**
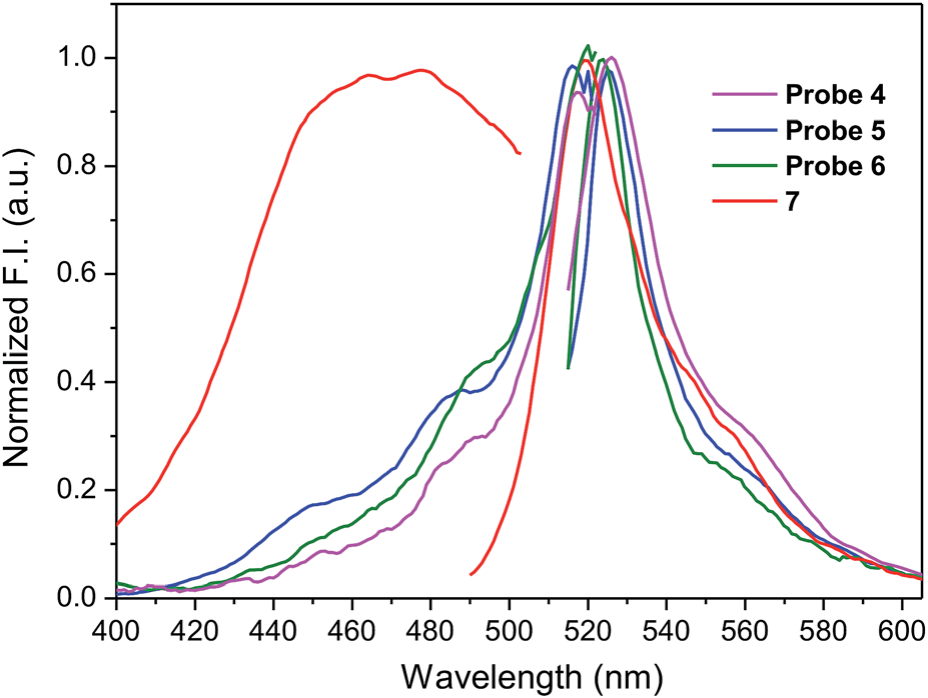
Fluorescence excitation and emission spectra of amino-BODIPY 7 and conjugates 4–6 (DMSO/HEPES buffer 2 : 1, 0.1 M, 7.4 pH).

To assess the effect of solvent polarity to OFF–ON effect, we mixed the HEPES buffer with DMSO (1 : 2 v/v). Based on the results from Table 1 and Fig. 4, the excitation as well as emission spectra of conjugates 4–6 exhibit bathochromic shift to approximately 520 nm and 530 nm, respectively and enhanced quantum yield.

**Fig. 5:**
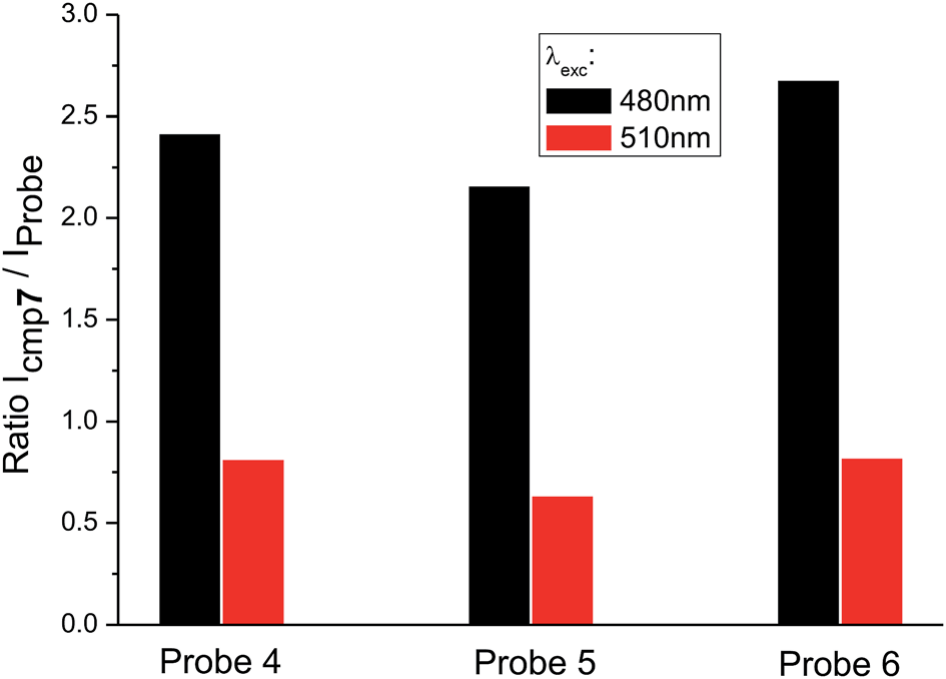
Ratio of fluorescence intensity of amino-BODIPY 7 to conjugates 4–6 at 530 nm after excitation of the appropriate couple at 480 nm or 510 nm (DMSO/HEPES buffer 2 : 1, 0.1 M, pH 7.4).

When the excitation wavelength *λ*_exc_ = 480 nm is applied, the emission intensity ratio for *λ*_em_ = 530 nm between amino-BODIPY 7 and its conjugates 4–6 is approximately 2–3, and hence, the OFF–ON effect is not as effective as in the HEPES buffer. When *λ*_exc_ = 510 nm is used, the fluorescence intensity is lower for amino-BODIPY 7 as compared to conjugates 4–6 (Fig. 5).

**Fig. 6:**
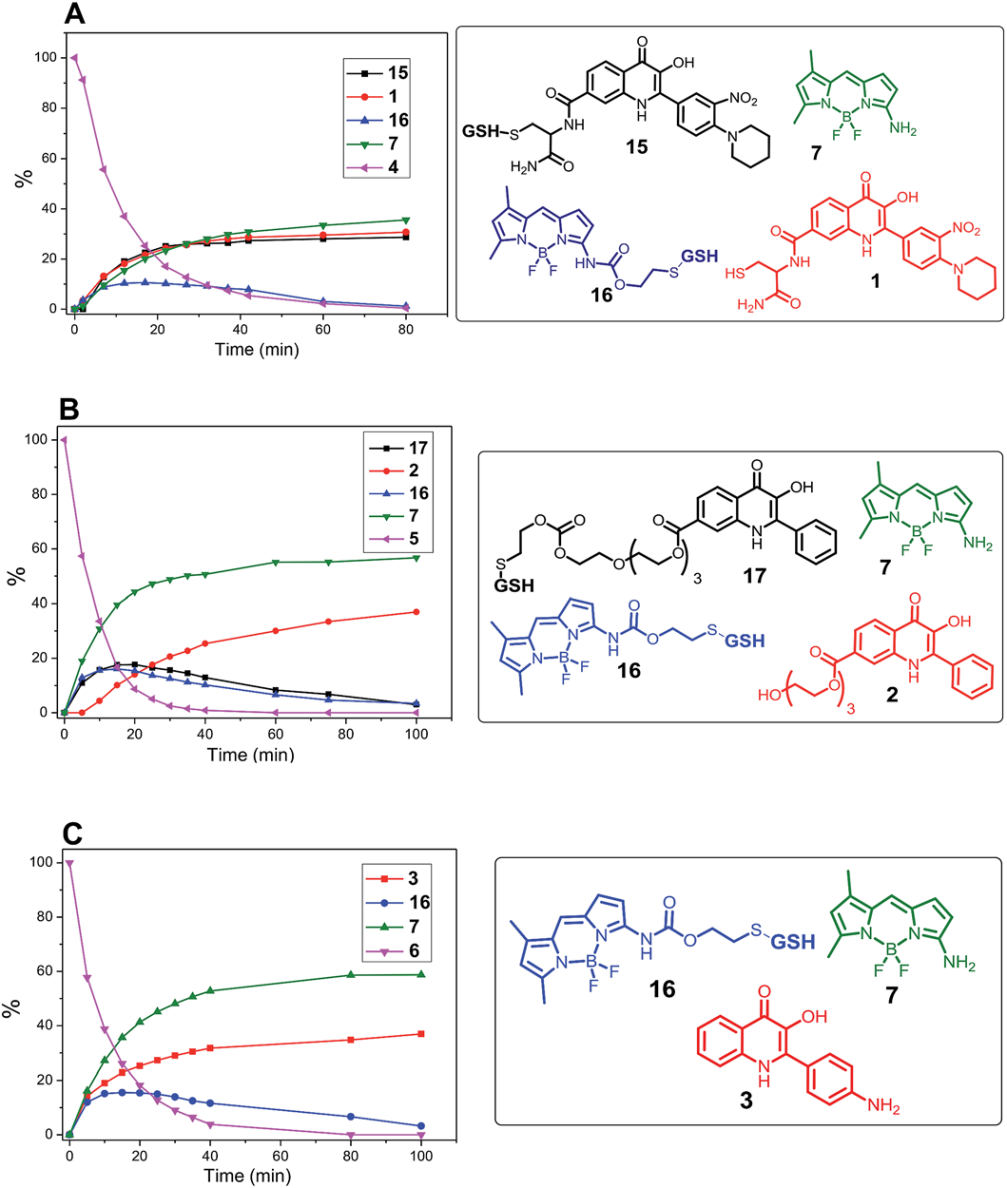
Monitoring the drug release from conjugates 4, 5, and 6 (0.5 mM; DMSO/HEPES buffer 2 : 1, 0.1 M; pH 7.4; 37 °C) by GSH (5 mM) by HPLC/MS.

To confirm the ability of disulfide linker to release the amino-BODIPY 7 and the model drugs 1–3 by thiols according to Scheme 1, conjugates 4–6 were treated with 0.5 mM glutathione at physiological conditions (37 °C, pH 7.4) and monitored by LC/MS. Because of the necessity to use a high concentration of the probes for such a detection, the present study was performed only in DMSO/HEPES buffer (2 : 1). As shown in Fig. 6, the treatment of prodrug 4 with GSH resulted in four products detected by HPLC. According to mass spectrometry, we detected the presence of the expected released drug 1 and amino-BODIPY 7 indicating complete cleavage of the conjugate, but also the formation of adducts 15 and 16 derived from the 3-HQ or BODIPY dye. Interestingly, the concentration of the adduct 15 increases in a time-dependent manner, which might be attributed to the equilibrium between 15 and 1. Conversely, the concentration of adduct 16 decreased with time because of the presence of self-immolative disulfide linker that prevents similar equilibrium.

The cleavage of the conjugates 5 and 6, wherein 3HQ is bound *via* carbonate and carbamate bonds, afforded the corresponding 3HQs 2 and 3, respectively. Although the GSH adducts 16 and 17 were also observed, these were subsequently converted to the final free 3HQ derivative 2 or 3 and amino-BODIPY 7 (Fig. 6B and C). The treatment using all prodrugs 4–6 was performed with 20 μM in the extracellular matrix.^33^ No cleavage was observed for any derivative in this case.

**Fig. 7:**
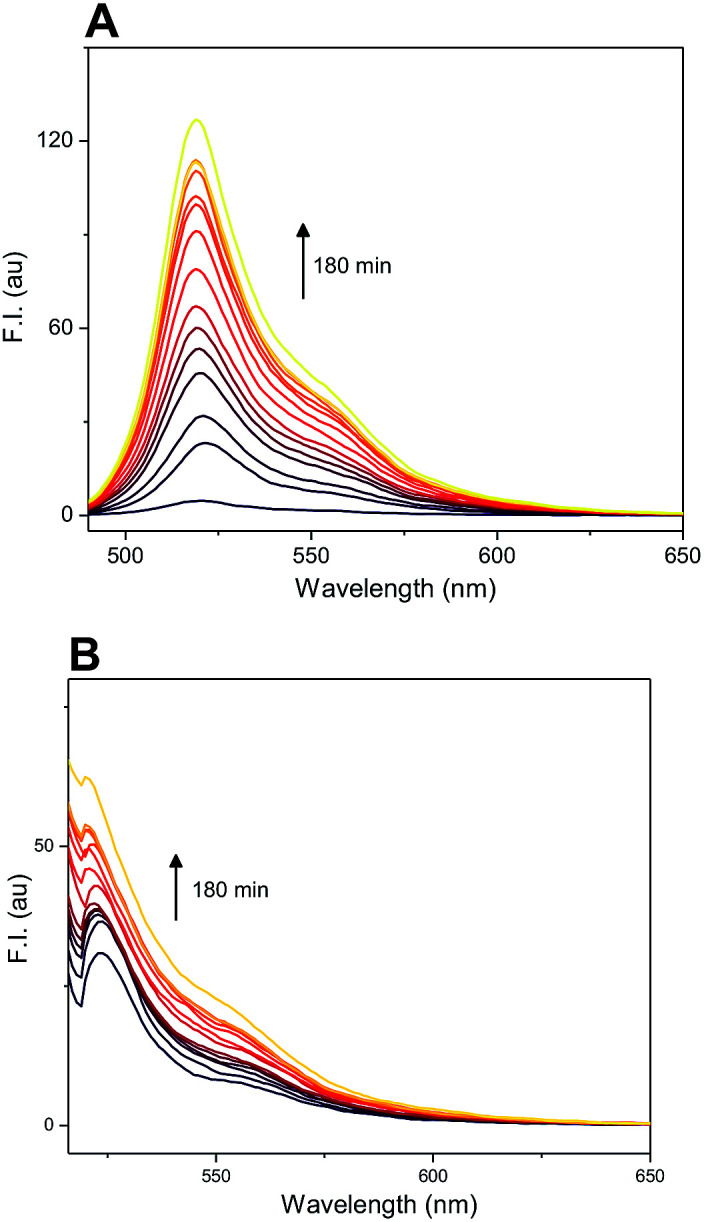
GSH-mediated cleavage of probe 4 monitored within 180 min by fluorescence emission at 525 nm after excitation at 480 nm (A) and 510 nm (B) (5 μM probe 4, 5 mM GSH; HEPES buffer, 0.1 M, pH 7.4, 37 °C).

The difference in fluorescence profile of amino-BODIPY 7 and conjugates 4–6 should enable efficient monitoring of the cleavage of conjugates *via* OFF–ON mode in HEPES buffer (Fig. 3), which is optimal for potential biological applications. Next we performed the GSH-mediated cleavage of probes 4–6 monitored by fluorescence with excitation at 480 nm as well as 510 nm and emission at 525 nm. Within 180 min we observed the expected enhancement of the fluorescence intensity for excitation at 480 nm and 510 nm (Fig. 7; Fig. S2 and S4†).

**Fig. 8:**
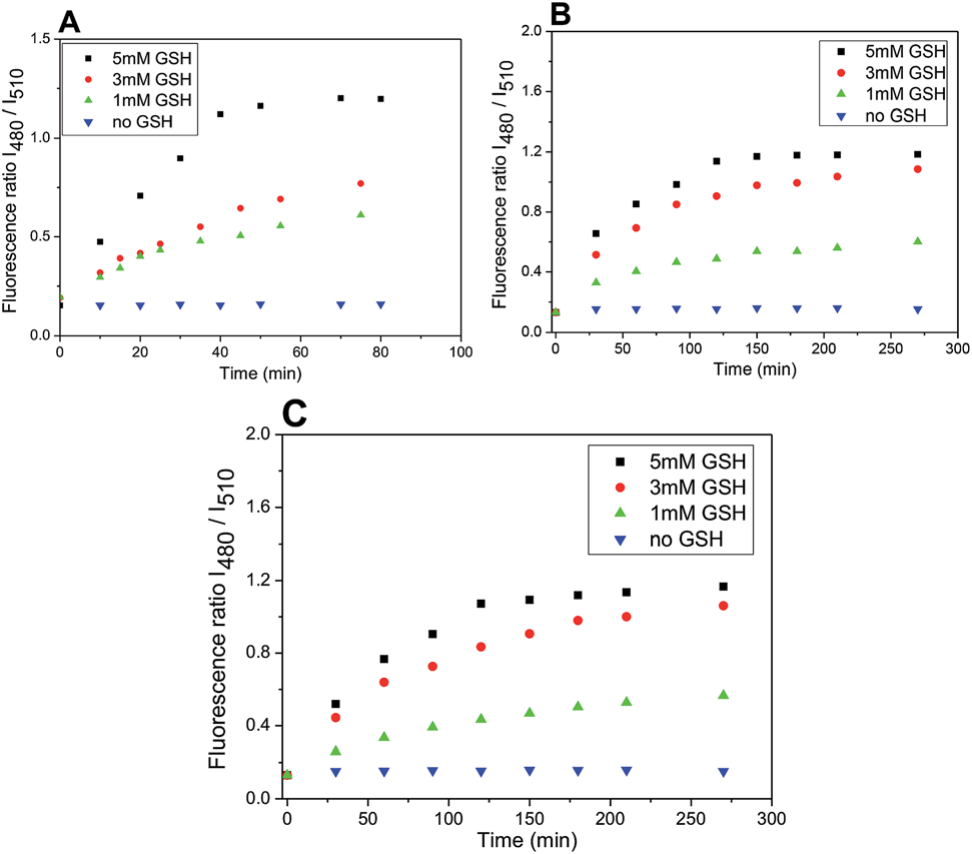
Ratio of emission intensities at 525 nm upon excitation at 480 and 510 nm in time after treatment with different concentrations of GSH (5, 3, 1 mM). Probe 4 (A); probe 5 (B); probe 6 (C) (5 μM probes 4–6; DMSO/HEPES buffer 2 : 1, 0.1 M, pH 7.4, 37 °C).

Furthermore, plotting the ratio of emission intensities at 525 nm after excitation at 480 nm and 510 nm (*I*_480_/*I*_510_) *vs.* time allows ratiometric fluorescence monitoring the cleavage in a concentration independent manner (Fig. 8).

**Fig. 9:**
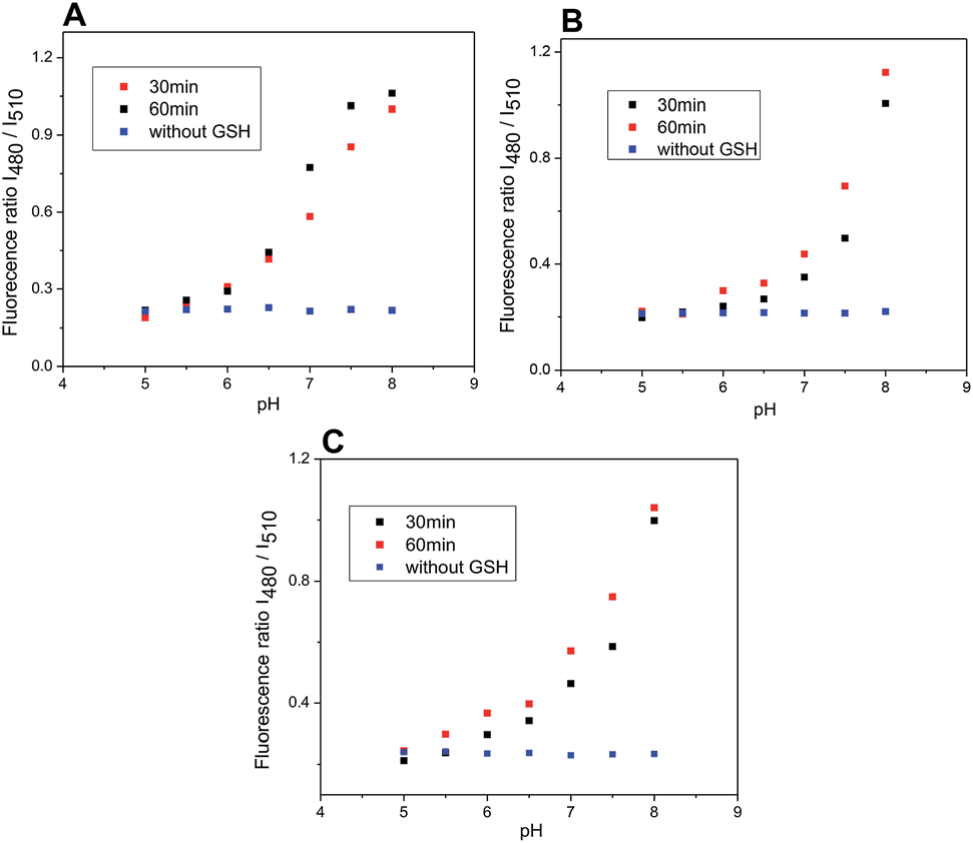
Ratio of emission intensities at 525 nm upon excitation at 480 and 510 nm after 30 and 60 minutes of incubation of probes 4–6 with GSH at various pH (5.0–8.0) and without GSH after 60 minutes. Probe 4 (A); probe 5 (B); probe 6 (C) (5 μM probe 4–6, 5 mM glutathione; DMSO/HEPES buffer 2 : 1, 0.1 M, 37 °C).

The rate of the GSH-mediated cleavage of disulfide bridge depends on the concentration of GSH. While 5 mM GSH normally present in some cancer cells,^14^ was sufficient for full cleavage of conjugate 4 within 50 min and 120 min for conjugates 5 and 6, respectively; the low concentrations of GSH caused only partial cleavage during the same period (Fig. 8). The limit of detection (LOD) for GSH was determined for compounds 5 and 6 (HEPES buffer 7.4 pH, 37 °C, 2 h incubation). Obtained LOD values were 305 nM and 752 nM, respectively (ESI,†Fig. 12). In addition, the cleavage is pH dependent and proceeds rapidly in basic conditions. In acidic medium, none or only little conjugate is cleaved (Fig. 9).

**Fig. 10:**
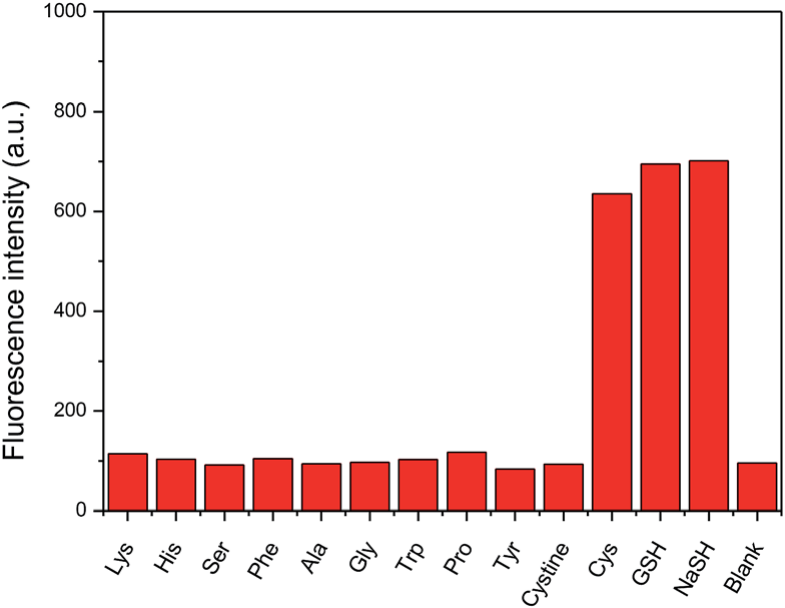
Fluorescence intensity enhancement upon incubation of probe 4 (5 μM) with various amino acids and NaSH (100 mM, DMSO/HEPES buffer 2 : 1, 0.1 M, pH 7.4) after 60 min. (*λ*_exc_ = 480 nm, *λ*_em_ = 525 nm).

As exemplified on probe 4 not only GSH but also cysteine, commonly present in a biological medium, can cause the amino-BODIPY 7 releasing. On the other hand, the system is resistant to all other amino acids (Fig. 10).

**Fig. 11:**
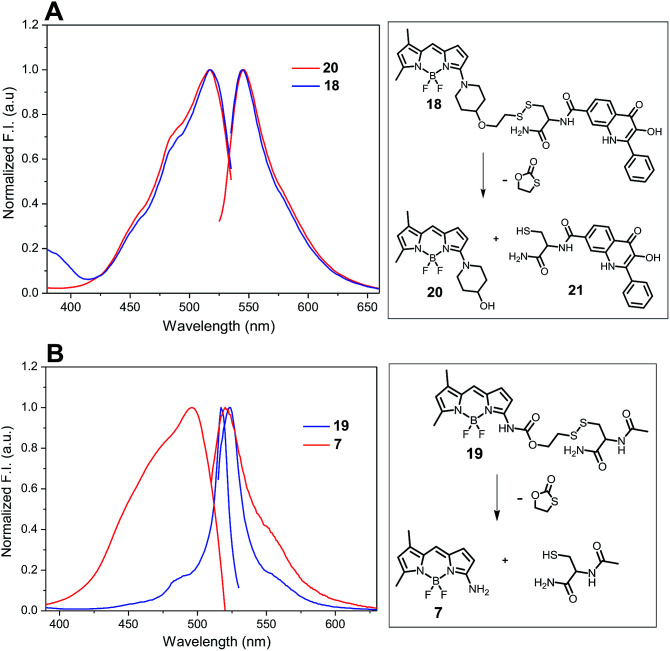
Normalized excitation and emission profile of compounds 18, 19, and 20 (5 μM in DMSO/HEPES buffer 2 : 1, 0.1 M, pH 7.4).

The release of amino-BODIPY 7 from conjugates 4–6 is accompanied by the release of drugs 1–3 (Fig. 6) and therefore enhancing the fluorescence of amino-BODIPY 7 reflects release of the drugs. Thus, the monitoring of the drug release is dependent only on the change of acylamino-BODIPY to amino-BODIPY derivative and is independent of the type of the drug. To prove this hypothesis, compounds 18, 19, and 20 with different substitutions on amino-BODIPY (Fig. 11) were synthesized as described in Scheme 3 (see ESI†). Conjugate 18 and released BODIPY dye 20 showed the same excitation/emission profile due to the same piperidyl substitution directly bound to the BODIPY scaffold. On the other hand, conjugate 19 effectuating amino-BODIPY 7 after cleavage follows the same excitation/emission profile changes as conjugates 4–6 (Fig. 11 and 4). Thus, we can conclude that the principle of OFF–ON effect is not influenced by the nature of a compound conjugated to BODIPY dye, but rather depends only on the transformation of acylamino-BODIPY to amino-BODIPY derivative.

**Fig. 12:**
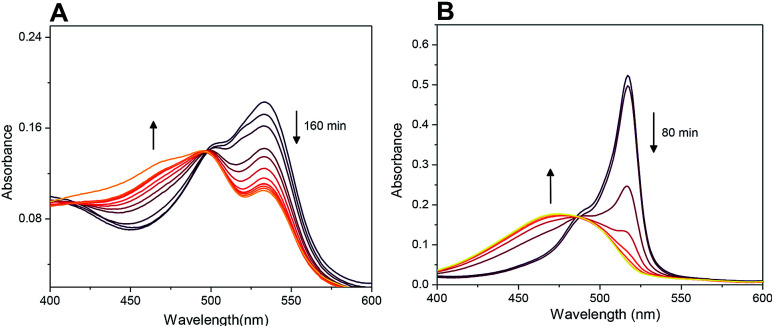
Monitoring of cleavage of probes 4 (A) and 19 (B) by UV/Vis spectroscopy (5 μM probes; 5 mM GSH, 0.1 M HEPES buffer, pH 7.4, 37 °C). For spectra highlighting change of fluorescence in time see Fig. 7 and S7.†

Probe 4-based monitoring of the cleavage by UV/Vis spectroscopy resulted in the hyperchromic as well as hypochromic shift with an isosbestic point at approximately 490 nm (Fig. 12). As the absorption profile of quinolinones 1–3 bound in conjugates 4–6 did not interfere with this wavelength (see ESI†), the isosbestic point was connected with the change of acylamino-BODIPY to amino-BODIPY regardless of the bound drug. This phenomenon could be supported by the same results from UV/Vis-based monitoring of cleavage of conjugate 19 bearing cysteine having no absorption in the UV/Vis region (Fig. 12).

**Fig. 13:**
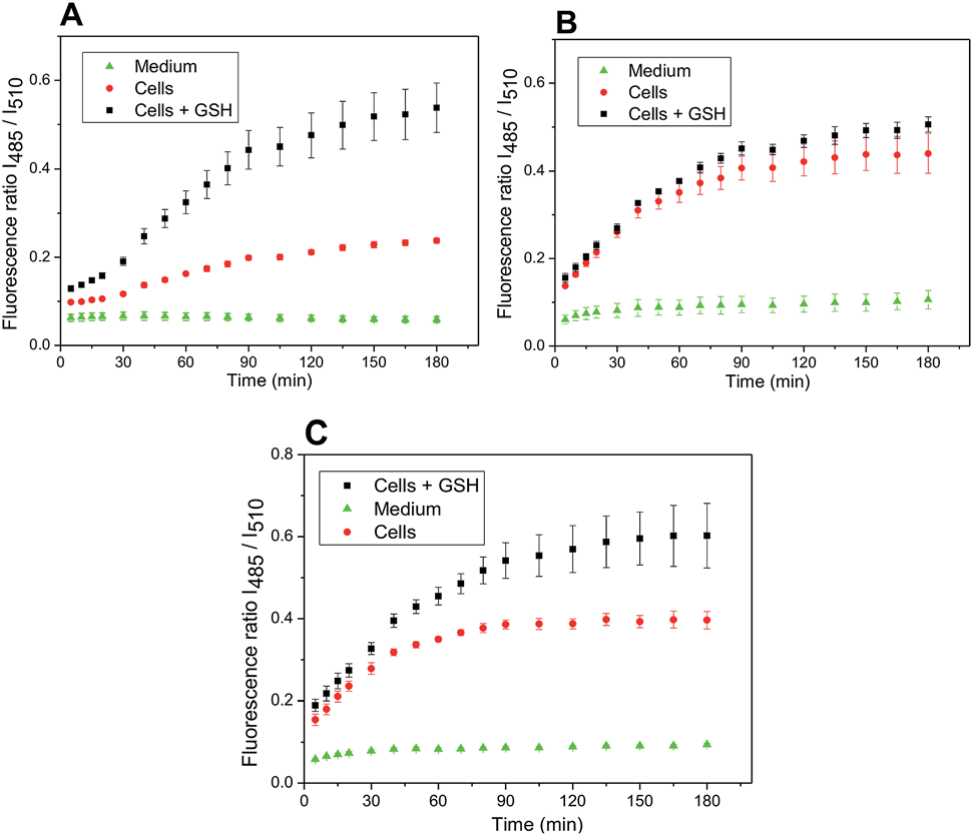
Ratio of emission intensities at 525 nm upon excitation at 485 nm and 510 nm in HeLa cells corresponding to the cleavage of conjugate 4 (A), 5 (B), and 6 (C) within the prescribed period.

The developed system might be used for monitoring the release of a drug inside the cancer cells, well known for the increased level of redox potential due to high concentration of thiols, mainly GSH.^14,15^ A maximal release of a drug within a time period corresponded to the maximal release of amino-BODIPY 7 from appropriate conjugate (Scheme 1; Fig. 6). To prove the real functioning of the system, we performed the cleavage experiment of conjugates 4–6 in HeLa cells that were pretreated with glutathione (20 mM). Due to the instrumental limitation, excitation at 485 nm was used instead of *λ*_exc_ = 480 nm. Although the inherent concentration of GSH in the cells was sufficient to cleave the disulfide conjugates 4–6, the cleavage was efficient when conjugates were added to the cells preincubated with additional GSH (Fig. 13), which is similar to the previous results (Fig. 8). The exception is conjugate 5 with the most susceptible carbonate linker between dye and model drug. Supposedly, the GSH concentration in HeLa cells is sufficiently high for maximal cleavage of the linker, and the preincubation with GSH does not accelerate the disruption of the disulfide bond.

**Fig. 14:**
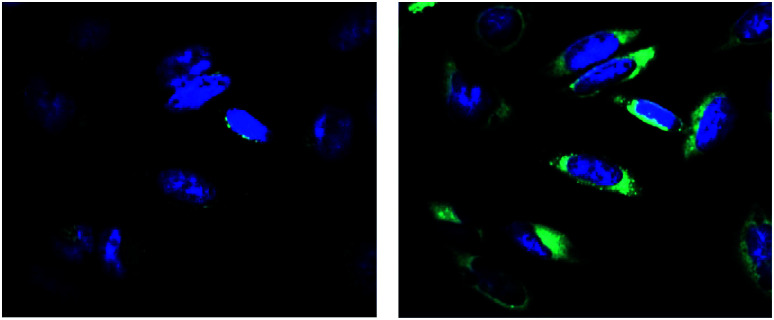
Fluorescence intensity of released amino-BODIPY 7 (green color) after cleavage of conjugate 4 inside the HeLa cells pretreated with glutathione at time 0 min (left) and 120 min (right). *λ*_exc_ = 485 nm.

The release of a drug inside the cells can be monitored *via* the OFF–ON effect by fluorescence microscopy. For this purpose, the HeLa cells were localized by staining the nuclei by Hoechst dye (blue color) and then treated with the conjugate 4, generating green emission due to the release of amino-BODIPY 7 and the corresponding drug in a time-dependent manner (Fig. 14).

**Fig. 15:**
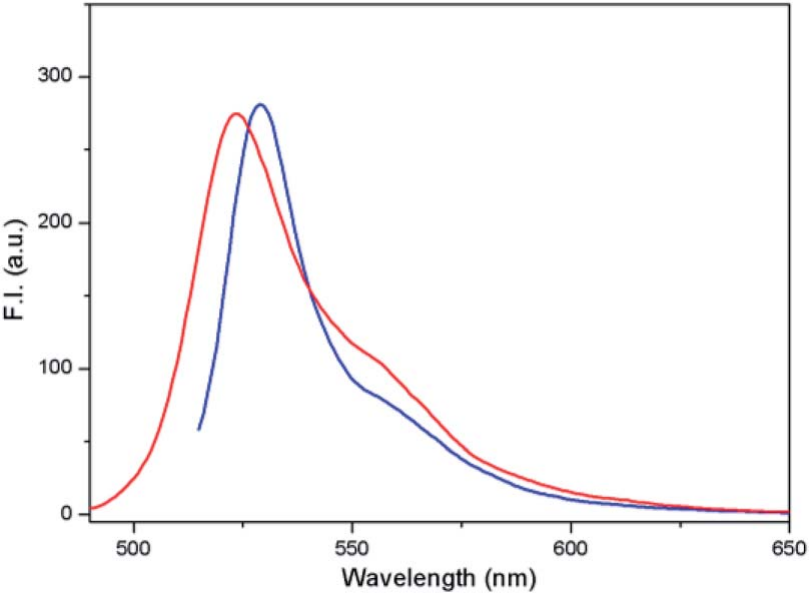
Fluorescence emission spectra of compound 4 (5 μM; DMSO/HEPES buffer 2 : 1, 0.1 M, pH 7.4) after excitation at 510 nm (blue) before cleavage and at 480 nm (red) after cleavage.

The other possible applicability of the introduced system can be demonstrated by the construction of a molecular electronic “selector” of two sources for one appliance. Therefore, the derivative 4 was studied in DMSO/HEPES (2 : 1 v/v) at a concentration of 5 μM. If none or only one of the above-mentioned conditions (lack of thiols and pH < 6) were fulfilled, the conjugate was stable (Fig. 9). However, when both conditions were fulfilled, the conjugate was cleaved. In the case of intensity of emission at 527 nm or 543 nm, the excitation at *λ*_exc_ = 510 nm before cleavage and at *λ*_exc_ = 480 nm after cleavage was similar (Fig. 15).

**Fig. 16:**
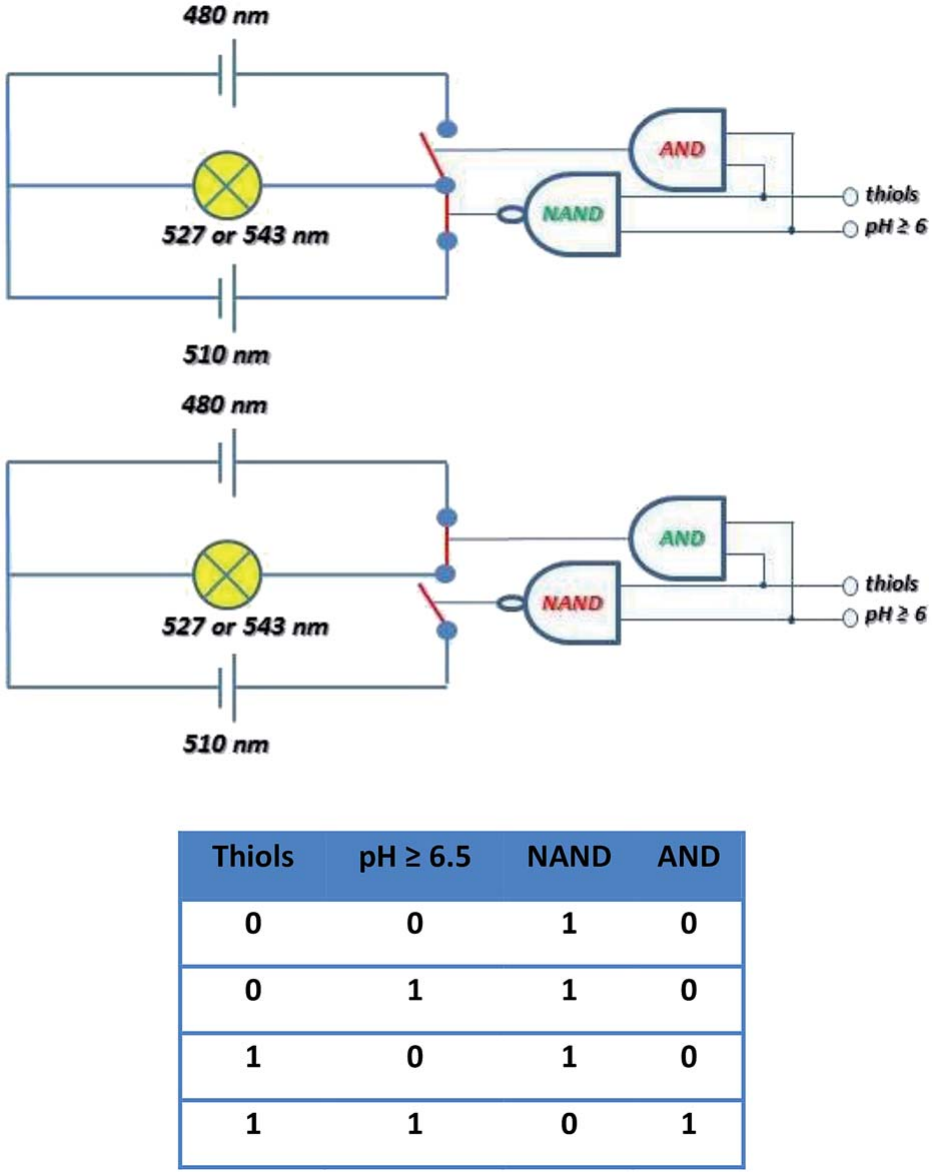
Schematic description of the selector based on cleavage of probe 4 able to switch the source of the light to maintain the same intensity and accuracy based on the truth table.

The molecular selector represented schematically in Fig. 16, wherein two circuits with different power supplies are connected to one light. The selector able to change the source is operated by the tandem of molecular gates AND and NAND operated by thiol and pH inputs. Before the conjugate cleavage the output of operator NAND is “one” and source 520 nm is active. When the conjugate is cleaved NAND operator has the output “zero” and immediately operator AND produces output “one. The source 480 nm is than active. This circuitry imitates “emergency power unit” for the generation of energy for the “bulb” after the failure of the main source.

## Conclusion

In conclusion, the conjugates of amino-BODIPY and model drugs bound by self-immolative linker were synthesized and analyzed using glutathione for their cleavage with respect to the change in fluorescent properties. The spectral differences between acylamino-BODIPY in the conjugates and amino-BODIPY released after the glutathione attack enabled the monitoring of the release based on two excitations/one emission ratiometric fluorescence or *via* the OFF–ON effect. Thus, the drug release corresponded to the release of the amino-BODIPY used for monitoring, rendering the system valuable for monitoring the release of non-fluorescent drugs. Moreover, the drug interactions are effectuated *via* their amino, hydroxy, or thiol group that renders versatility to the compound structure. The rate of drug release is dependent on the concentration of glutathione as well as on the pH of the solution. The monitoring of the model drug release *via* ratiometric fluorescence as well as the OFF–ON effect was also verified in cancer cells with native and artificially increased concentration of glutathione. Furthermore, the UV/Vis spectroscopy allows the estimation of the concentration of conjugates independently on the extent of cleavage.

The altered excitation wavelengths post-cleavage also showed similar emission, and hence, was used for the construction of molecular electronic selector. This selector was operated by two logic gates for irreversible switching of the two sources to maintain the intensity of one light.

The concept of acylamino-BODIPY conjugates affording the amino-BODIPY dye after thiol-mediated cleavage based on the altered fluorescence offers development of various applications in chemical biology and molecular electronics in the future.

## Conflicts of interest

There are no conflicts to declare.

## Supplementary Material

RA-009-C9RA03472B-s001
