# Supplementary material for: Amino-BODIPY as the ratiometric fluorescent sensor for monitoring drug release or “power supply” selector for molecular electronics
Source: RSC Adv. 2019 Aug 13;9(43):25075–83. doi: 10.1039/c9ra03472b (PMC9069925; doi:10.1039/c9ra03472b)
Supplement: RA-009-C9RA03472B-s001 [file RA-009-C9RA03472B-s001.pdf]

Electronic Supplementary Material (ESI) for RSC Advances.
This journal is © The Royal Society of Chemistry 2019

SUPPORTING INFORMATION

Amino-BODIPY as the Ratiometric Fluorescent Sensor for Monitoring Drug Release or "Power Supply" Selector for Molecular Electronics

Martin Porubský[a], Soňa Gurská[b], Jarmila Stanková[b], Marián Hajdúch[b], Petr Džubák[b] and Jan Hlaváč[b]*

[a] Department of Organic Chemistry, Faculty of Science, Palacký University Olomouc, 17. listopadu 12, 771 46 Olomouc, Czech Republic.

[b] Institute of Molecular and Translation Medicine, Faculty of Medicine and Dentistry, Palacký University, Hněvotínská 5, 779 00 Olomouc, Czech Republic. E-mail: jan.hlavac@upol.cz

Contents

Synthesis of quinolinone derivatives 1 and 21 3
Synthesis of quinolinone derivative 2 6
Synthesis of BODIPY derivatives 7 and 20 7
Synthesis of conjugates 4, 18, 19 8
Synthesis of conjugates 5, 6 11
^{1}H and ^{13}C NMR spectra of prepared compounds 15
Fluorescence monitoring of the conjugates 4-6 cleavage 35
Figure S1 35
Figure S2 35
Figure S3 36
Figure S4 36
Figure S5 37
Figure S6 37
Figure S7 38
Detection limit 38
Figure S8 39
References 40

Synthesis of quinolinone derivatives 1 and 21

The derivative **1** and **21** were synthesised according to the following scheme.

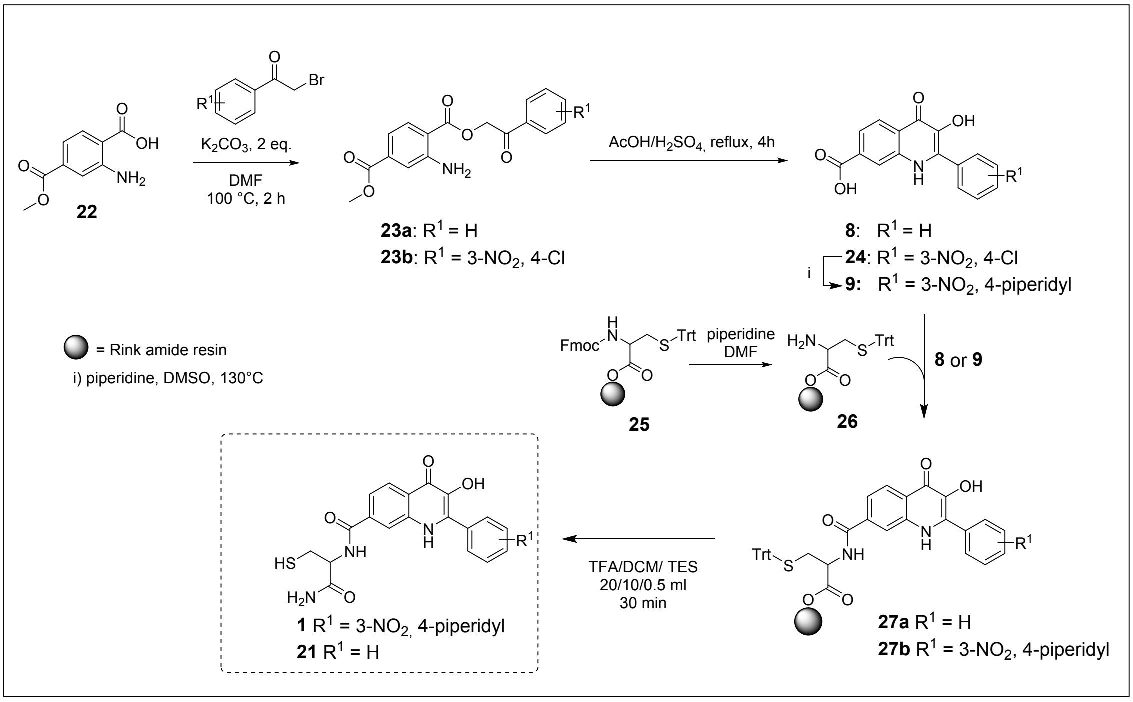

General procedure to methyl-1-phenacylesters of 2-aminoterephthalic acid 23.

Suspension of 2-amino-4-(methoxycarbonyl)benzoic acid **22**[1] (359 mg, 1.84 mmol) and K_2CO_3 (254 mg, 1.84 mmol) in DMF (8mL) was heated up to 90°C and stirred for 1h. Then the reaction mixture was allowed to cool to room temperature and corresponding bromoacetophenone (1.84 mmol) was subsequently added portionwise. Resulting mixture was stirred overnight at room temperature. The reaction mixture was then poured on the ice and precipitate was filtered off to give corresponding phenacylester **23**. Compounds were used as crude materials for the next step.

4-methyl 1-(2-oxo-2-phenylethyl) 2-aminoterephthalate (23a)

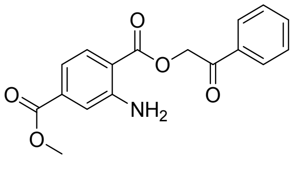

Yield: 95 %. **^{1}H NMR** $CDCl_3$ δ = 8.05 – 8.02 (m, 1H), 7.96 – 7.93 (m, 2H), 7.63 – 7.58 (m, 1H), 7.51 – 7.47 (m, 2H), 7.36 – 7.34 (m, J = 1.4 Hz, 1H), 7.27 – 7.24 (m, 1H), 5.54 (s, 2H), 3.89 (s, J = 5.3 Hz, 3H). **^{13}C NMR** δ = 192.38, 167.00, 166.73, 150.49, 135.38, 134.37, 134.23, 132.07, 129.15, 128.05, 118.26, 116.80, 113.30, 66.46, 52.57. **MS** (ESI) calcd for M+H: 314.10. Found: 314.09.

1-(2-(4-chloro-3-nitrophenyl)-2-oxoethyl) 4-methyl 2-aminoterephthalate (23b)

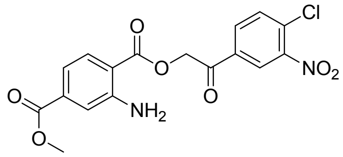

Yield: 93 %. **^{1}H NMR** DMSO-d$_6$ δ = 8.64 (d, *J* = 1.9 Hz, 1H), 8.28 (dd, *J* = 8.4, 1.9 Hz, 1H), 8.01 (d, *J* = 8.4 Hz, 1H), 7.91 (d, *J* = 8.4 Hz, 1H), 7.48 (d, *J* = 1.4 Hz, 1H), 7.09 (dd, *J* = 8.4, 1.5 Hz, 1H), 6.88 (s, 2H), 5.75 (s, 2H), 3.84 (d, *J* = 9.4 Hz, 3H). **^{13}C NMR** δ = 191.18, 166.02, 165.86, 151.32, 147.97, 134.54, 133.56, 132.58, 132.47, 131.39, 130.13, 124.83, 117.79, 114.45, 110.99, 66.83, 52.36. **MS** (ESI) calcd for M+H: 393.0484. Found: 393.07.

General procedure for synthesis of 3-hydroxy-2-phenylquinolin-4(1*H*)-one 8 and 24

Phenacylester intermediates **23a** or **23b** (7.65 mmol) were subjected to cyclization and subsequent hydrolysis reaction in the mixture of AcOH (60mL) and H_2SO_4 (15mL) under reflux for 4h. After completeness of the reaction the mixture was poured on the ice and precipitate was filtered off to afford corresponding 3-hydroxy-2-phenylquinolones **8** or **24**.

3-hydroxy-4-oxo-2-phenyl-1,4-dihydroquinoline-7-carboxylic acid (8)

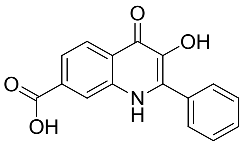

Yield: 75 %. **^{1}H NMR** DMSO-d$_6$ δ= 8.45 (d, *J* = 1.1 Hz, 1H), 8.27 (dd, *J* = 8.4, 4.3 Hz, 1H), 7.86 – 7.82 (m, 2H), 7.80 (dd, *J* = 8.6, 1.3 Hz, 1H), 7.62 – 7.53 (m, 3H). **^{13}C NMR** δ= 168.17, 166.83, 138.71, 137.09, 134.59, 132.39, 131.83, 129.78, 129.46, 128.46, 124.86, 123.73, 122.08, 121.07. **MS** (ESI) calcd for M+H 282.08. Found: 282.25.

2-(4-chloro-3-nitrophenyl)-3-hydroxy-4-oxo-1,4-dihydroquinoline-7-carboxylic acid (24)

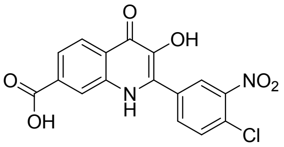

Yield: 78 %. **^{1}H NMR** DMSO-d$_6$ δ= 8.55 (d, *J* = 1.9 Hz, 1H), 8.39 (s, 1H), 8.25 (d, *J* = 8.5 Hz, 1H), 8.18 (dd, *J* = 8.4, 1.9 Hz, 1H), 8.01 (d, *J* = 8.5 Hz, 1H), 7.78 (d, *J* = 8.5 Hz, 1H) **^{13}C NMR** δ = 169.10, 166.78, 147.48, 139.34, 137.44, 134.65, 132.68, 132.20, 131.75, 130.13, 126.27, 125.86, 125.08, 123.99, 121.97, 121.02. **MS** (ESI) calcd for M+H: 361.02. Found: 361.04.

3-hydroxy-2-(3-nitro-4-(piperidin-1-yl)phenyl)-4-oxo-1,4-dihydroquinoline-7-carboxylic acid (9)

Solution of starting compound **24** (500 mg, 1.39 mmol) in DMSO (5mL) was treated with piperidine (344 μl, 3.48 mmol) and the resulting mixture was heated to 130°C overnight in the sealed tube. The reaction mixture was poured on the ice and resulting solid was filtered off.

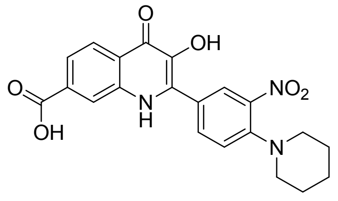

Yield: 78%. ^{1}**H NMR DMSO-d$_6$** δ= 13.14 (bs, 1H), 11.74 (bs, 1H), 8.40 (s, 1H), 8.31 (d, *J*= 2.0 Hz, 1H), 8.22 (d, J = 8.5 Hz, 1H), 8.03 (d, *J* = 8.3 Hz, 1H), 7.74 (dd, J = 8.5, 1.3 Hz, 1H), 7.43 (d, *J* = 8.8 Hz, 1H), 3.13 – 3.07 (m, 4H), 1.66 – 1.57 (m, 6H). ^{13}**C NMR** δ = 170.04, 167.39, 146.76, 140.79, 139.51, 137.84, 134.82, 132.76, 130.89, 127.20, 125.53, 124.49, 123.48, 121.94, 121.24, 120.88, 52.27, 25.90, 23.92. **MS** (ESI) calcd for M+H: 410.1347. Found: 410.37.

General procedure for synthesis of N-(1-amino-3-mercapto-1-oxopropan-2-yl)-3-hydroxy-2-phenyl-4(1*H*)-quinolinone-7-carboxamides 1 and 21

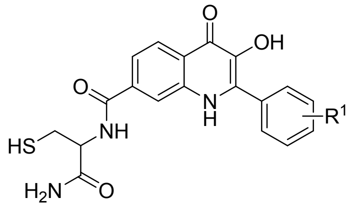

1 R^1 = 3-NO$_2$, 4-piperidyl
21 R^1 = H

Resin **25**

The Rink resin (1.0g, loading 0.6 mmol) was treated with solution of piperidine/DMF for 30 min, washed with DMF and DCM three times and then treated with solution of Fmoc-Cys(Trt)-OH (1.05 g, 1.8 mmol), HOBt (243 mg, 1.8 mmol) and DIC (279 ul, 1.8 mmol) in mixture of DCM/DMF (6 mL, 1:1). After 3 hours resin was washed with DMF and DCM three times to give resin **25** directly used for the next step.

Resin **26**

Resin **25** was treated with piperidine/DMF for 30 minutes and washed with DMF and DCM three times. The resin was used directly for the next step.

Resin **27a** or **27b**

Resin **26** was treated with the solution of **8** or **9** (1.2 mmol), HOBt (135 mg, 1.2 mmol) and DIC (155ul, 1.2 mmol) in DMF/Pyridine (6 mL, 1:1) for 3 hours and washed 3 times with DMF and DCM.

Derivatives **1** and **21**

Resin **25** was treated in mixture of TFA/DCM/TES (20:10:0.5) for 1h. The solvents were evaporated and the product was precipitated with Et_2O to afford pure desired products **1** or **21**.

N-(1-amino-3-mercapto-1-oxopropan-2-yl)-3-hydroxy-2-(3-nitro-4-(piperidin-1-yl)phenyl)-4-oxo-1,4-dihydroquinoline-7-carboxamide (1)

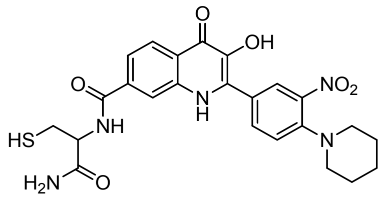

Yield: 94 %. **^{1}H NMR DMSO-d$_6$** δ= 11.74 (bs, 1H), 8.61 (d, J= 7.9 Hz, 1H), 8.30 (s,1H), 8.25 (s, 1H), 8.21 (d, J= 8.5 Hz, 1H), 8.03 (d, J= 8.5 Hz, 1H), 7.75 (d, J= 8.5 Hz, 1H), 7.54 (s, 1H), 7.43 (d, J= 8.8 Hz, 1H), 7.22 (s, 1H), 4.54 (dd, J= 8.3, 12.7 Hz, 1H), 3.10 (s, 4H), 2.95 – 3.03 (m, 1H), 2.83 – 2.92 (m, 1H), 2.39 (t, J= 8.4 Hz, 1H), 1.60 - 1.64 (s, 6H). **^{13}C NMR** δ= 172.20, 170.13, 166.58, 145.75, 140.80, 139.22, 137.95, 136.50, 134.83, 130.69, 127.19, 125.15, 123.67, 123.56, 120.88, 120.75, 119.28, 56.51, 52.29, 26.52, 25.91, 23.93. **HRMS** (ESI) m/z calcd for [$C_{24}H_{25}N_5O_6S$]+H: 512.1598; found: 512.1608.

N-(1-amino-3-mercapto-1-oxopropan-2-yl)-3-hydroxy-4-oxo-2-phenyl-1,4-dihydroquinoline-7-carboxamide (21)

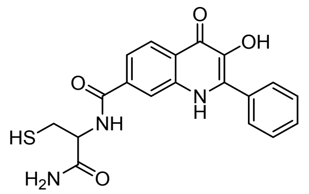

Yield: 95 %. **^{1}H NMR DMSO-d$_6$** δ = 11.82 (bs, 1H), 8.60 (d, *J*= 7.9 Hz, 1H), 8.26 (s, 1H), 8.22 (d, *J*= 8.50 Hz, 1H), 7.82 (d, *J*= 7.2 Hz, 2H), 7.76 (dd, *J*= 1.2, 8.5 Hz, 1H), 7.50 – 7.60 (m, 4H), 7.22 (s, 1H), 4.50 - 4.58 (m, 1H), 2.94 – 3.03 (m, 1H), 2.82 – 2.93 (m, 1H), 2.39 (t, *J*= 8.4 Hz, 1H) **^{13}C NMR** δ = 171.69, 169.48, 166.09, 138.55, 137.38, 135.93, 132.64, 132.09,129.03, 129.28, 128.31, 124.59, 123.09, 120.26, 118.90, 55.97, 26.00. **HRMS** (ESI) m/z calcd for $C_{19}H_{17}N_3O_4S$: 383.0940; found: 383.0944.

Synthesis of quinolinone derivative 2

The derivative **2** was synthesised according to the following scheme

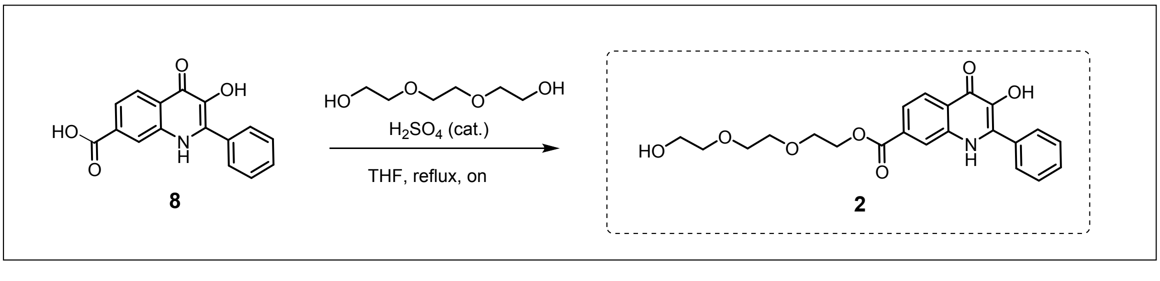

2-(2-(2-hydroxyethoxy)ethoxy)ethyl 3-hydroxy-4-oxo-2-phenyl-1,4-dihydroquinoline-7-carboxylate (2)

The suspension of starting acid **8**[2] (300 mg, 1.07 mmol) in THF was mixed with triglycol (1.0 ml, 10.7 mmol) and catalytic amount of H_2SO_4 (3 drops). The reaction mixture was heated to reflux overnight.

After the full consumption of the starting material, reaction was stopped and ethylacetate (100ml) was added. Resulting solution was washed with 10% aq. $NaHCO_3$, 3 times with water and with brine. Organic layer was dried over Na_2SO_4 and concentrated under reduced pressure to obtain 285 mg (65 %) of pure product **2.**

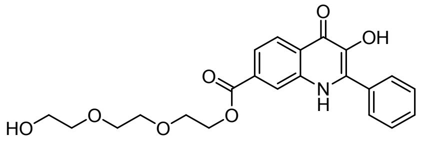

Yield: 65 %. **^{1}H NMR DMSO-d$_6$** δ= 11.87 (s, 1H), 8.46 (s, 1H), 8.29 – 8.25 (m, 1H), 7.84 (d, *J* = 7.2 Hz, 2H), 7.76 (dd, *J* = 8.6, 1.5 Hz, 1H), 7.60 – 7.53 (m, 3H), 4.47 – 4.44 (m, 2H), 3.81 – 3.78 (m, 2H), 3.62 – 3.60 (m, 2H), 3.56 – 3.53 (m, *J* = 2.3 Hz, 2H), 3.48 – 3.45 (m, *J* = 5.1 Hz, 2H), 3.43 – 3.40 (m, *J* = 4.4 Hz, 2H). **^{13}C NMR** δ= 169.58, 165.37, 139.10, 137.23, 132.69, 132.04, 130.91, 129.49, 129.29, 128.34, 125.29, 121.06, 120.93, 72.38, 69.92, 69.77, 68.36, 64.54, 60.22. **HRMS** (ESI) m/z calcd for [C22H22NO7]+H: 414.1547. Found: 414.1545.

Synthesis of BODIPY derivatives 7 and 20

The derivative **7** and **20** were synthesized according to the following scheme:

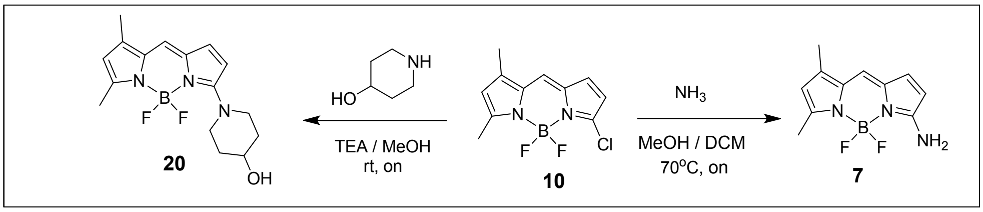

1,3-dimethyl-4,4-difluoro-4-bora-3a,4a-diaza-5-amino-s-indacene (7)

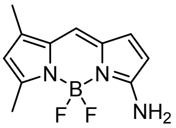

The solution of **10**[3] (200mg, 0.79 mmol) in dry dichloromethane (5mL) was treated with 7.0 M NH_3 in methanol (2.2 mL). Resulting mixture was stirred at 70°C overnight. After full consumption of starting material, reaction mixture was cooled down to room temperature and concentrated under vacuum. Crude product was purified by column chromatography (EtOAc/n-hexane 1:2) to give dark solid compound. Yield 131 mg (71%).

^{1}H NMR $CDCl_3$ δ= 6.91 (d, 1H, *J* = 4.6 Hz), 6.72 (s, 1H), 5.89 – 5.93 (m, 2H), 5.50 (s, 2H), 2.45 (s, 3H), 2.16 (s, 3H). **^{13}C NMR** δ= 160.61, 159.39, 147.59, 134.19, 133.34, 132.12, 116.91, 115.93, 109.74, 14.12, 11.11. **HRMS** (ESI) m/z calcd for $[C_{11}H_{12}BF_2N_3]$-H: 234.1009; found: 234.1009.

N-(4-hydroxypiperidine)-1,3-dimethyl-4,4-difluoro-4-bora-3a,4a-diaza-5-amino-s-indacene (20)

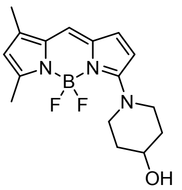

Starting compound **10**[3] (500 mg, 1.97 mmol) was dissolved in MeOH (10ml) and TEA (412 µl, 2.95 mmol), then 4-hydroxypiperidine (219 mg, 2.17 mmol) was added to the solution. Resulting mixture was stirred overnight at room temperature, extracted with EtOAc and washed three times with water. Drying over Na_2SO_4 was followed by evaporation to dryness. Yield 604 mg (96 %) of pure product.

^{1}H NMR $CDCl_3$ δ= 6.94 (d, 1H, *J*= 4.8 Hz), 6.65 (s, 1H), 6.13 (d, 1H, J= 4.8 Hz), 5.92 (s, 1H), 4.11 – 4.21 (m, 2H), 3.97 – 4.05 (m, 1H), 3.56 – 3.65 (m, 2H), 2.46 (s, 3H), 2.17 (s, 3H), 2.02 – 2.10 (m, 2H), 1.69 – 1.78 (m, 2H), 1.63 (bs, 1H). **^{13}C NMR** δ= 161.13, 146.52, 134.35, 133.42, 131.80, 129.92, 115.85, 115.54, 110.90, 66.73, 47.51, 34.41, 14.27, 11.03. **HRMS** (ESI) m/z calcd for $[C_{16}H_{20}BF_2N_3O]$+H: 320.1740; found: 320.1741.

Synthesis of conjugates 4, 18, 19

The derivative **4, 18** and **19** were synthesised according to the following scheme

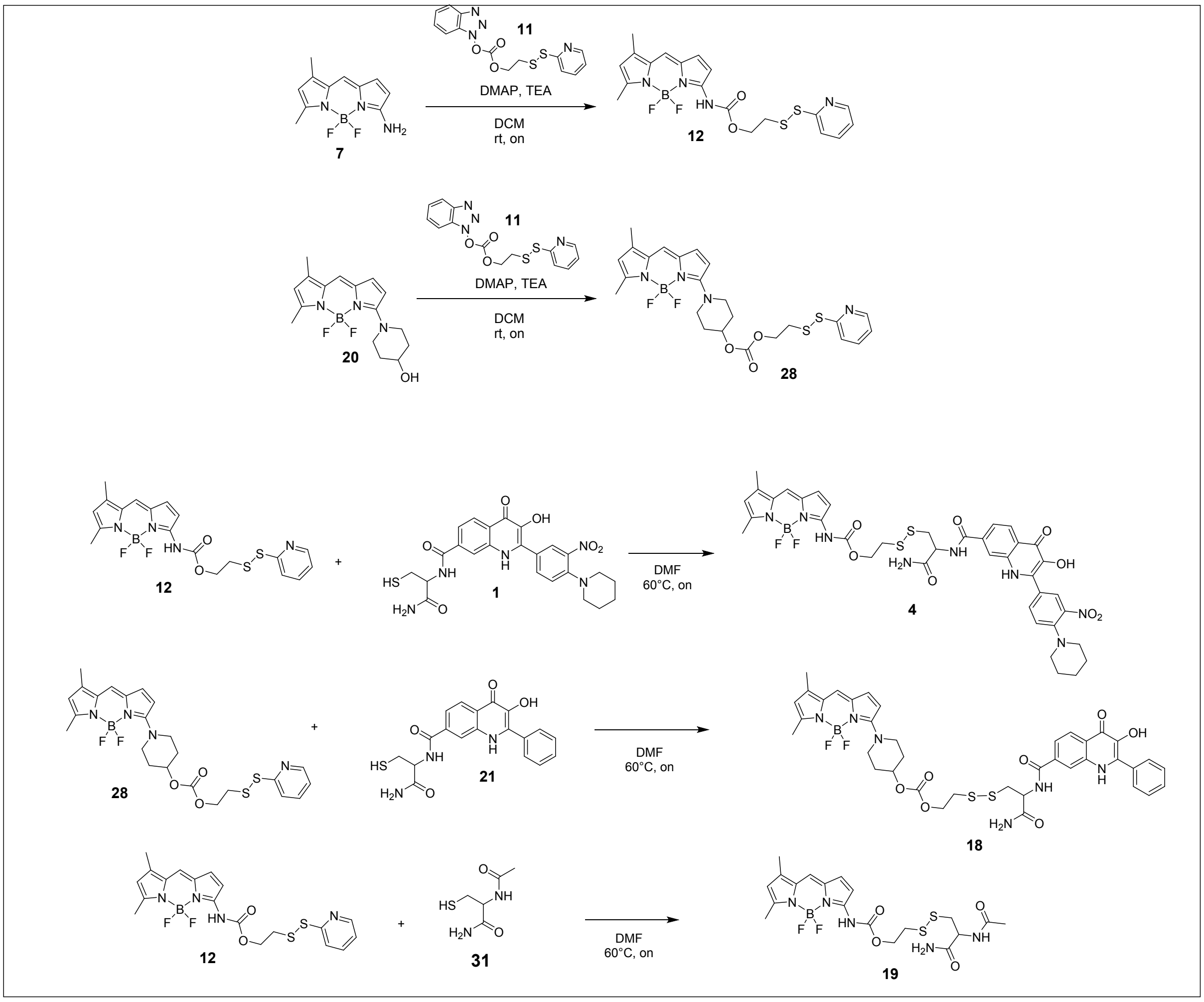

1,3-dimethyl-4,4-difluoro-4-bora-3a,4a-diaza-5-(carbamoyl-ethylpyridinyldisulfide)-s-indacene (12)

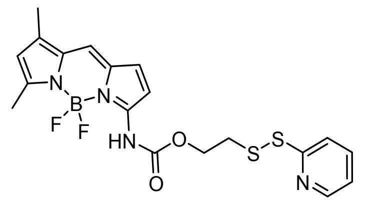

To a stirred solution of **7** (100 mg, 0.425 mmol) in dry DCM (10mL) DMAP (68 mg), TEA (178 µl) and activated disulfide linker **11**[4] (193 mg) were added portionwise. Resulting mixture was stirred at room temperature for 3 h. After the reaction was complete solvent was evaporated and crude mixture was directly purified by column chromatography (EtOAc/n-hexane 1:2) to afford 182 mg (95 %) of pure product **12**.

Yield: 95 %. **^{1}H NMR** $CDCl_3$ δ= 8.46 (d, 1H, J= 4.3 Hz), 8.00 (s, 1H, NH), 7.59 – 7.70 (m, 2H), 7.05 – 7.10 (m, 2H), 6.99 (s, 1H), 6.97 (d, 1H, J= 4.3 Hz), 6.83 (d, 1H, J= 3.9 Hz), 6.03 (s, 1H), 4.45 (t, 2H, J= 6.4 Hz), 3.08 (t, 2H, 6.4 Hz), 2.50 (s, 3H), 2.21 (s, 3H). **^{13}C NMR** δ= 159.54, 155.70, 151.43, 149.87, 149.58, 141.25, 137.20, 133.61, 130.87, 129.74, 122.08, 121.06, 120.12, 118.98, 109.26, 63.90, 37.21, 14.64, 11.34. **HRMS** (ESI) m/z calcd for $[C_{19}H_{19}BF_2N_4O_2S_2]$+H: 449.1083; found: 449.1089.

1-(5,5-difluoro-7,9-dimethyl-5H-4l4,5l4-dipyrrolo[1,2-c:2',1'-f][1,3,2]diazaborinin-3-yl)piperidin-4-yl (2-(pyridin-2-yldisulfanyl)ethyl) carbonate (28)

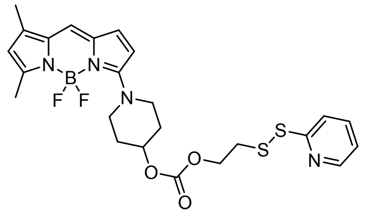

To a stirred solution of **20** (40 mg, 0.13 mmol) in dry DCM (5mL) DMAP (20 mg, 0.17 mmol), TEA (53 µl, 0.17 mmol) and activated disulfide linker **11**[4] (66 mg, 0.20 mmol) was added portionwise. Resulting mixture was stirred at room temperature overnight. After the reaction was complete solvent was evaporated and crude mixture was directly purified by column chromatography (EtOAc/n-hexane 1:2) to afford 66 mg (99 %) of pure product **28**.

^{1}H NMR $CDCl_3$ δ = 8.49 (dd, J = 4.5, 1.3 Hz, 1H), 7.72 – 7.64 (m, 3H), 7.15 – 7.11 (m, 1H), 6.95 (d, J = 4.7 Hz, 1H), 6.68 (s, 1H), 6.11 (d, J = 4.7 Hz, 1H), 5.93 (s, 1H), 4.95 – 4.89 (m, 1H), 4.42 (t, J = 6.49 Hz, 2H), 4.05 – 3.99 (m, 2H), 3.79 – 3.73 (m, 2H), 3.09 (t, J = 6.5 Hz, 2H), 2.46 (s, 3H), 2.18 (s, 3H), 2.15 – 2.05 (m, 2H), 1.96 – 1.87 (m, 2H). **^{13}C NMR** δ = 160.98, 159.42, 154.17, 149.86, 147.06, 137.20, 134.13, 133.37, 132.42, 130.07, 121.07, 120.02, 119.28, 116.06, 110.60, 73.00, 65.48, 47.11, 37.02, 30.76, 14.31, 11.07. **HRMS** (ESI) m/z calcd for $C_{24}H_{27}BF_2N_4O_3S_2$: 532.1586; found: 532.1588

General procedure for synthesis of the conjugates 4,18,19.

Starting compound **12** or **28** (0.06 mmol) and 2-acetamido-3-mercaptopropanamide **31** or corresponding quinolinone **1** or **21** (1,13) (0.08 mmol) were dissolved in dry DMF (3mL) and heated to 60°C. Resulting reaction mixture was stirred overnight. After complete consumption of starting materials the reaction mixture was cooled down and diluted with mixture ethylacetate-methanol (5:1) followed by washing with water repetaed 3 times. Organic layer was dried over Na_2SO_4 and

concentrated. Obtained crude product was purified by HPLC (AcCN/ammonium acetate (10mM) buffer 30:70 to 60:40 gradient) affording pure compounds **4**, **18** or **19**.

2-((3-amino-2-(3-hydroxy-2-(3-nitro-4-(piperidin-1-yl)phenyl)-4-oxo-1,4-dihydroquinoline-7-carboxamido)-3-oxopropyl)disulfanyl)ethyl (5,5-difluoro-7,9-dimethyl-5H-4l4,5l4-dipyrrolo[1,2-c:2',1'-f][1,3,2]diazaborinin-3-yl)carbamate (4)

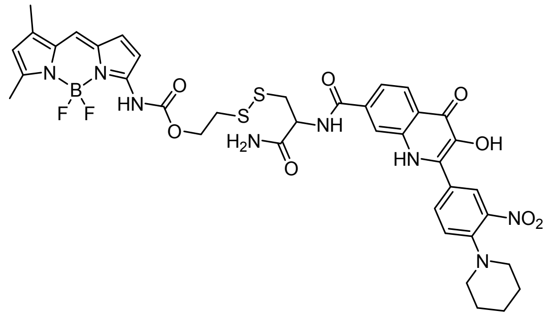

[1]**H NMR** DMSO-d_6 δ= δ 11.23 (s, 1H), 8.09 – 7.99 (m, 3H), 7.96 (d, *J* = 7.1 Hz, 1H), 7.79 (d, *J* = 8.2 Hz, 1H), 7.65 (s, 1H), 7.39 (d, *J* = 8.3 Hz, 1H), 7.00 (s, 1H), 6.85 (d, *J* = 8.5 Hz, 1H), 6.74 – 6.70 (m, 1H), 6.67 – 6.63 (m, 1H), 6.42 – 6.37 (m, 1H), 6.21 (s, 1H), 5.71 (s, 1H), 4.68 – 4.58 (m, 1H), 4.18 – 4.06 (m, 2H), 3.12 – 2.99 (m, 4.4 Hz, 2H), 2.96 – 2.87 (m, 2H), 2.79 (s, 4H), 2.72 – 2.67 (m, 2H), 2.12 (s, 3H), 1.87 (s, 3H), 1.40 (s, 4H), 1.32 (s, 2H). [13]**C NMR** δ= 166.77, 166.59, 155.12, 151.11, 149.07, 146.73, 141.81, 141.18, 140.50, 137.70, 135.94, 135.69, 134.10, 133.27, 130.80, 129.38, 127.21, 126.65, 125.00, 122.72, 122.35, 122.11, 120.25, 119.83, 118.83, 118.74, 108.87, 63.99, 52.10, 40.87, 40.77, 36.46, 25.56, 23.69, 14.34, 11.09. **HRMS** (ESI) m/z calcd for $[C_{38}H_{39}BF_2N_8O_8S_2]$-H : 847.2310; found: 847.2307. Yield: 34 %.

2-((3-amino-2-(3-hydroxy-4-oxo-2-phenyl-1,4-dihydroquinoline-7-carboxamido)-3-oxopropyl)disulfanyl)ethyl (1-(5,5-difluoro-7,9-dimethyl-5H-4l4,5l4-dipyrrolo[1,2-c:2',1'-f][1,3,2]diazaborinin-3-yl)piperidin-4-yl) carbonate (18)

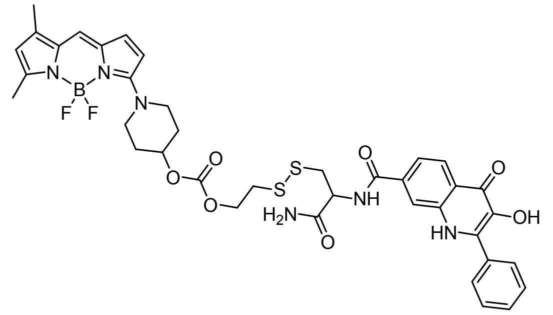

[1]**H NMR DMSO-d_6** δ= 11.81 (s, 1H), 8.81 (d, 1H, *J* = 7.7 Hz), 8.30 – 8.20 (m, 2H), 8.04 (s, 1H), 7.82 (d, 2H, *J* = 6.5 Hz), 7.75 (d, 1H, *J* = 8.2 Hz), 7.64 – 7.48 (m, 4H), 7.21 – 7.32 (m, 2H), 6.94 (s, 1H), 6.57 (d, 1H, *J* = 4.2 Hz), 5.90 (s, 1H), 4.84 (s, 1H), 4.72 (s, 1H), 4.33 (s, 2H), 4.05 (s, 2H), 3.75 (s, 2H), 3.20 – 3.11 (m, 2H), 2.98 – 3.09 (m, 2H), 2.30 (s, 3H), 2.12 (s, 3H), 2.03 (s, 2H), 1.71 (s, 2H). [13]**C NMR** δ= 171.71, 169.65, 166.07, 160.82, 153.58, 143.25, 138.58, 137.41, 135.84, 134.63, 132.38, 132.09, 129.26, 128.78, 128.27, 124.61, 123.13, 121.64, 121.33, 120.09, 119.41, 118.90, 114.97, 114.62, 112.90, 72.56, 65.18, 52.61, 46.76, 40.43, 36.04, 30.49, 13.87, 13.85, 10.61. **HRMS** (ESI) m/z calcd for $[C_{38}H_{39}BF_2N_6O_7S_2]$-H : 803.2299; found: 803.2298. Yield: 32 %.

3-(((2-((2-acetamido-3-amino-3-oxopropyl)disulfanyl)ethoxy)carbonyl)amino)-5,5-difluoro-7,9-dimethyl-5H-dipyrrolo[1,2-c:2',1'-f][1,3,2]diazaborinin-4-ium-5-uide (19)

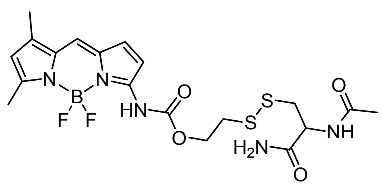

^{1}H NMR CDCl$_3$ δ= 8.09 (s, 1H), 8.00 (s, 1H), 7.01 (s, 1H), 6.99 (d, *J* = 4.4 Hz, 1H), 6.85 (d, *J* = 4.3 Hz, 1H), 6.77 – 6.72 (m, 2H), 6.05 (s, 1H), 5.88 (s, 1H), 4.81 – 4.77 (m, 1H), 4.50 – 4.47 (m, 2H), 3.12 (t, *J* = 6.5 Hz, 2H), 3.00 (t, *J* = 6.5 Hz, 2H), 2.50 (s, 3H), 2.21 (s, 3H), 2.02 (s, 3H). **^{13}C NMR** δ= 172.43, 170.75, 162.71, 155.87, 151.69, 149.41, 141.48, 133.70, 130.90, 129.72, 122.21, 119.10, 109.25, 77.41, 77.16, 76.91, 64.18, 52.25, 40.80, 37.05, 36.64, 31.58, 23.20, 14.71, 11.40. **HRMS** (ESI) m/z calcd for [$C_{19}H_{23}BF_2N_5O_4S_2$]-H : 498.1258; found: 498.1255. Yield: 38 %.

Synthesis of conjugates 5, 6

The conjugates **5** and **6** were synthesised according to the following scheme:

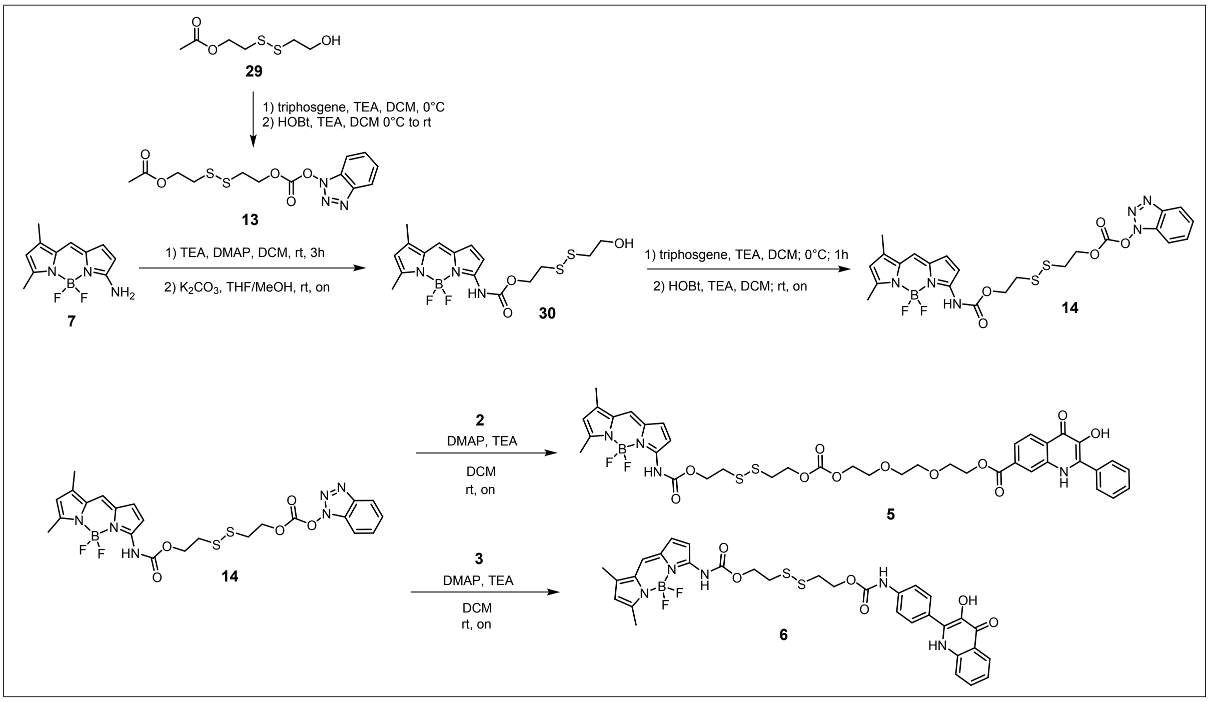

2-((2-((((1H-benzo[d][1,2,3]triazol-1-yl)oxy)carbonyl)oxy)ethyl)disulfanyl)ethyl acetate (13)

2-((2-Hydroxyethyl)disulfanyl)ethyl acetate[5] **29** (0.5g, 2.55 mmol) was dissolved in DCM (20mL) and cooled to 0°C. Then triethylamine (391 µl, 2.81 mmol) was added followed by addition of solution of triphosgene (250 mg, 0.84 mmol) in DCM (5 mL). After stirring for 1 hour at 0°C the solution of HOBt (379 mg, 2.81 mmol) and triethylamine (391 µl, 2.81 mmol) in DCM (5mL) was added slowly to the reaction mixture. The reaction mixture was then stirred overnight at room temperature. Reaction solution was diluted with DCM (100mL) and washed with water 3x80mL and brine. Organic layer was dried over Na_2SO_4 and concentrated under reduced pressure to obtain the desired product. Yield 774 mg (85%)

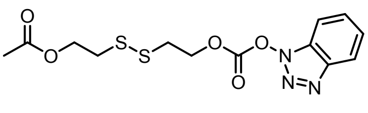

^{1}H NMR $CDCl_3$ δ = 8.23 – 8.20 (m, 1H), 8.02 – 7.99 (m, 1H), 7.79 – 7.75 (m, 1H), 7.57 – 7.53 (m, 1H), 4.80 (t, *J* = 6.7 Hz, 2H), 4.34 (t, *J* = 6.5 Hz, 2H), 3.13 (t, *J* = 6.7 Hz, 2H), 2.98 (t, *J* = 6.5 Hz, 2H), 2.07 (s, 3H). **^{13}C NMR** δ = 170.90, 147.29, 133.52, 132.98, 126.55, 120.75, 115.96, 115.27, 77.42, 77.16, 76.91, 66.64, 62.32, 37.42, 36.62, 20.97. **HRMS** (ESI) m/z calcd for $[C_{13}H_{16}N_3O_5S_2]$+H: 358.0526; found: 358.0523.

Synthesis of 5,5-difluoro-3-(((2-((2-hydroxyethyl)disulfanyl)ethoxy)carbonyl)amino)-7,9-dimethyl-5H-dipyrrolo[1,2-c:2',1'-f][1,3,2]diazaborinin-4-ium-5-uide (30)

DMAP (68 mg, 0.557 mmol), TEA (178 µl, 1.277 mmol) and disulfide linker **13** (228 mg, 0.638 mmol) was added portionwise to a stirred solution of **7** (100 mg, 0.425 mmol) in dry DCM (10mL). Resulting mixture was stirred at room temperature for overnight. After the reaction was complete solvent was evaporated, dissolved in THF/MeOH 3:1 (10 mL) and treated with K_2CO_3 (176 mg, 1.275mmol). After stirring overnight ethylacetate (100mL) was added and organic phase was washed 3 times with water, dried over Na_2SO_4 and concentrated under vacuum. Crude product was purified by column chromatography (hexane/EtOAc 2:1) to afford pure product 31. Yield 113 mg (64 %).

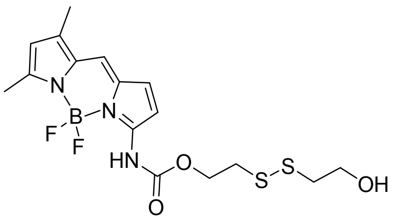

^{1}H NMR $CDCl_3$ δ = 8.08 (s, 1H), 7.01 (s, 1H), 7.00 (d, *J* = 4.4 Hz, 1H), 6.88 (d, *J* = 4.3 Hz, 1H), 6.05 (s, 1H), 4.49 (t, *J* = 6.7 Hz, 2H), 3.91 (t, *J* = 5.8 Hz, 1H), 3.00 (t, *J* = 6.7 Hz, 2H), 2.92 (t, *J* = 5.8 Hz, 2H), 2.51 (s, 3H), 2.23 (s, 3H). **^{13}C NMR** δ = 155.77, 151.65, 149.57, 141.35, 133.68, 130.91, 129.78, 122.15, 119.03, 109.29, 64.36, 60.40, 41.84, 37.05, 14.66, 11.36. **HRMS** (ESI) m/z calcd for $[C_{16}H_{19}BF_2N_3O_3S_2]$-H: 414.0934; found: 414.0930.

3-(((2-((2-((((1H-benzo[d][1,2,3]triazol-1-yl)oxy)carbonyl)oxy)ethyl)disulfanyl)ethoxy)carbonyl)amino)-5,5-difluoro-7,9-dimethyl-5H-dipyrrolo[1,2-c:2',1'-f][1,3,2]diazaborinin-4-ium-5-uide (14)

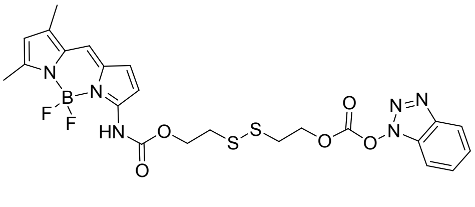

Solution of TEA (214 uL, 1.536 mmol) and triphosgene (146 mg, 0.492 mmol) dissolved in DCM (1 mL) was slowly added to a solution of compound **30** (510 mg, 1.229 mmol) in dry DCM (6 mL) at 0°C. Resulting reaction mixture was stirred for 2h at 0°C. Subsequently the solution of TEA (214 uL, 1.536 mmol) and HOBt (183 mg, 1.352 mmol) dissolved in DCM (2 mL) was added at 0°C. Reaction was allowed to warm to room temperature and was stirred overnight. Then EtOAc (200mL) was added to reaction mixture and organic layer was washed with water and brine. Organic layer was dried over Na_2SO_4 and concentrated under reduced pressure to give product **14**. Yield 657 mg (93 %).

^{1}H NMR $CDCl_3$ δ = 8.18 (d, *J* = 8.5 Hz, 1H), 8.04 (s, 1H), 7.96 (d, *J* = 8.4 Hz, 1H), 7.75 – 7.71 (m, 1H), 7.53 – 7.48 (m, 1H), 6.98 – 6.93 (m, 2H), 6.84 (d, *J* = 4.3 Hz, 1H), 6.02 (s, 1H), 4.80 (t, *J* = 6.7 Hz, 2H), 4.49 (t, *J* = 5.9 Hz, 2H), 3.16 (t, *J* = 6.7 Hz, 2H), 3.05 (t, *J* = 6.5 Hz, 2H), 2.47 (s, 3H), 2.21 (s, 3H). **^{13}C NMR** δ = 155.76, 151.52, 149.49, 147.23, 141.33, 133.64, 133.45, 132.87, 130.87, 129.73, 126.40, 122.10, 119.02, 115.81, 115.26, 109.22, 66.63, 64.16, 41.85, 37.46, 37.06, 36.64, 14.62, 11.36. **HRMS** (ESI) m/z calcd for $[C_{23}H_{22}BF_2N_6O_5S_2]$-H: 575.1160; found: 575.1149.

General procedure for synthesis conjugates **5** and **6**.

Starting compound **14** (0.06 mmol) and corresponding quinolinone derivative **2** or **3** (0.06 mmol) were dissolved in dry DCM (3mL). Subsequntly TEA (0.18 mmol) and DMAP (0.18 mmol) were added to the stirred solution of starting materials. Resulting reaction mixture was stirred overnight. After complete consumption of starting materials the mixture was diluted with ethylacetate and washed three times by water. Organic layer was dried over Na_2SO_4 and concentrated. Obtained crude product was purified by HPLC (AcCN/ammonium acetate buffer 30:70 to 60:40 gradient) affording pure compounds **5** or **6**.

1-((5,5-difluoro-7,9-dimethyl-5H-4l4,5l4-dipyrrolo[1,2-c:2',1'-f][1,3,2]diazaborinin-3-yl)amino)-1,10-dioxo-2,9,11,14,17-pentaoxa-5,6-dithianonadecan-19-yl 3-hydroxy-4-oxo-2-phenyl-1,4-dihydroquinoline-7-carboxylate (**5**)

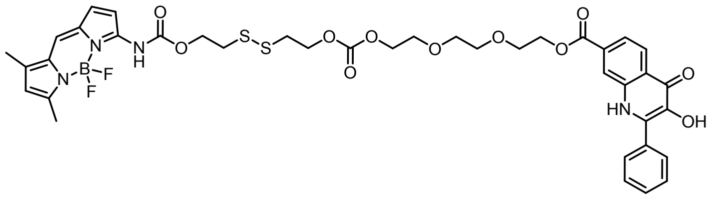

^{1}H NMR DMSO-d_6 δ= 11.83 (s, 1H), 8.46 (s, 1H), 8.26 (d, *J* = 8.5 Hz, 1H), 7.84 (d, *J* = 7.3 Hz, 2H), 7.75 (d, *J* = 8.6 Hz, 1H), 7.55 (dt, *J* = 24.6, 7.0 Hz, 4H), 7.24 (d, *J* = 4.0 Hz, 1H), 6.78 (t, *J* = 5.6 Hz, 1H), 6.20 (d, *J* = 9.0 Hz, 1H), 4.43 (dt, *J* = 12.7, 5.9 Hz, 4H), 4.30 (t, *J* = 6.3 Hz, 2H), 4.20 – 4.13 (m, 2H), 3.81 – 3.75 (m, 2H), 3.66 – 3.59 (m, 4H), 3.56 (dd, *J* = 5.7, 3.2 Hz, 2H), 3.07 (t, *J* = 6.4 Hz, 2H), 3.02 (t, *J* = 6.1 Hz, 2H), 2.42 (d, *J* = 6.5 Hz, 3H), 2.23 (d, *J* = 5.6 Hz, 3H). **^{13}C NMR** δ= 169.53, 165.30, 154.86, 154.32, 151.36, 148.92, 141.48, 139.01, 137.23, 133.02, 132.57, 131.98, 131.56, 130.89, 129.42, 129.22, 128.26, 125.20, 124.09, 123.59, 120.98, 120.86, 118.88, 109.09, 69.80, 69.70, 68.32, 68.12, 66.86, 65.13, 64.42, 63.77, 54.71, 36.29, 36.18, 14.15, 10.90. **HRMS** (ESI) m/z calcd for [C39H40BF2N4O11S2]-H: 853.2202; found: 853.2191. Yield: 35 %.

2-((2-(((4-(3-hydroxy-4-oxo-1,4-dihydroquinolin-2-yl)phenyl)carbamoyl)oxy)ethyl)disulfanyl)ethyl (5,5-difluoro-7,9-dimethyl-5H-4l4,5l4-dipyrrolo[1,2-c:2',1'-f][1,3,2]diazaborinin-3-yl)carbamate (**6**)

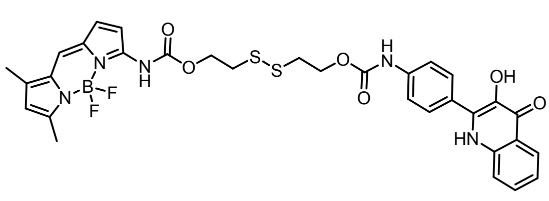

^{1}H NMR DMSO-d_6 δ= 11.66 (s, *J* = 13.0 Hz, 1H), 8.11 (d, *J* = 8.0 Hz, 1H), 7.72 (d, *J* = 8.3 Hz, 1H), 7.68 – 7.64 (m, 1H), 7.60 (s, 1H), 7.33 (s, 1H), 7.31 (s, 1H), 7.25 (d, *J* = 4.4 Hz, 1H), 6.79 (d, *J* = 4.4

Hz, 1H), 6.71 (d, *J* = 8.6 Hz, 2H), 6.21 (s, 1H), 5.75 (s, 1H), 4.46 – 4.38 (m, 4H), 3.11 – 3.03 (m, 4H), 2.43 (s, 3H), 2.23 (s, 3H). **^{13}C NMR** δ= 169.44, 162.26, 154.92, 152.66, 151.32, 150.94, 148.87, 143.39, 141.54, 139.07, 133.04, 131.64, 131.58, 130.78, 129.79, 129.40, 124.69, 123.67, 122.90, 118.90, 118.47, 116.86, 113.24, 109.13, 65.90, 63.82, 54.87, 36.21, 14.17, 10.91. **HRMS** (ESI) m/z calcd for [C32H31BF2N5O6S2]-H: 692.1626; found: 692.1624. Yield: 40 %.

^{1}H and ^{13}C NMR spectra of prepared compounds

Compound **1** (DMSO-d6)

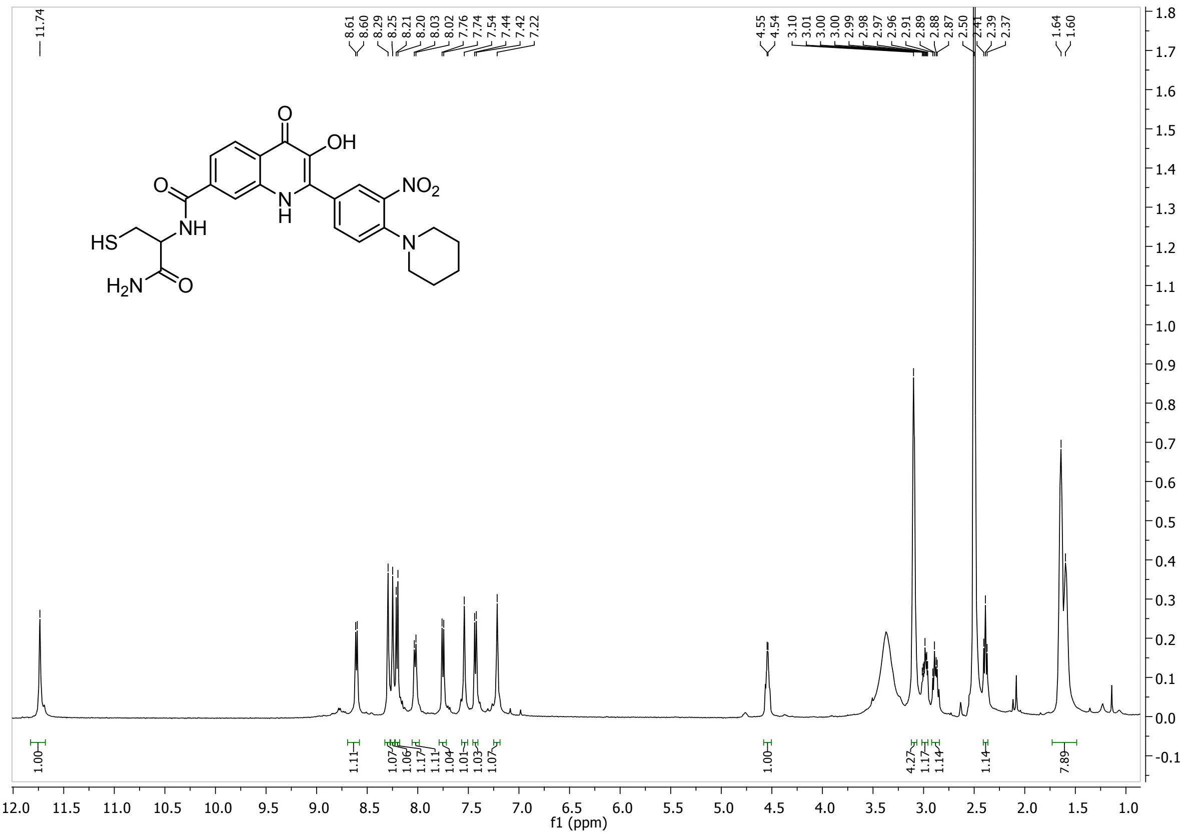

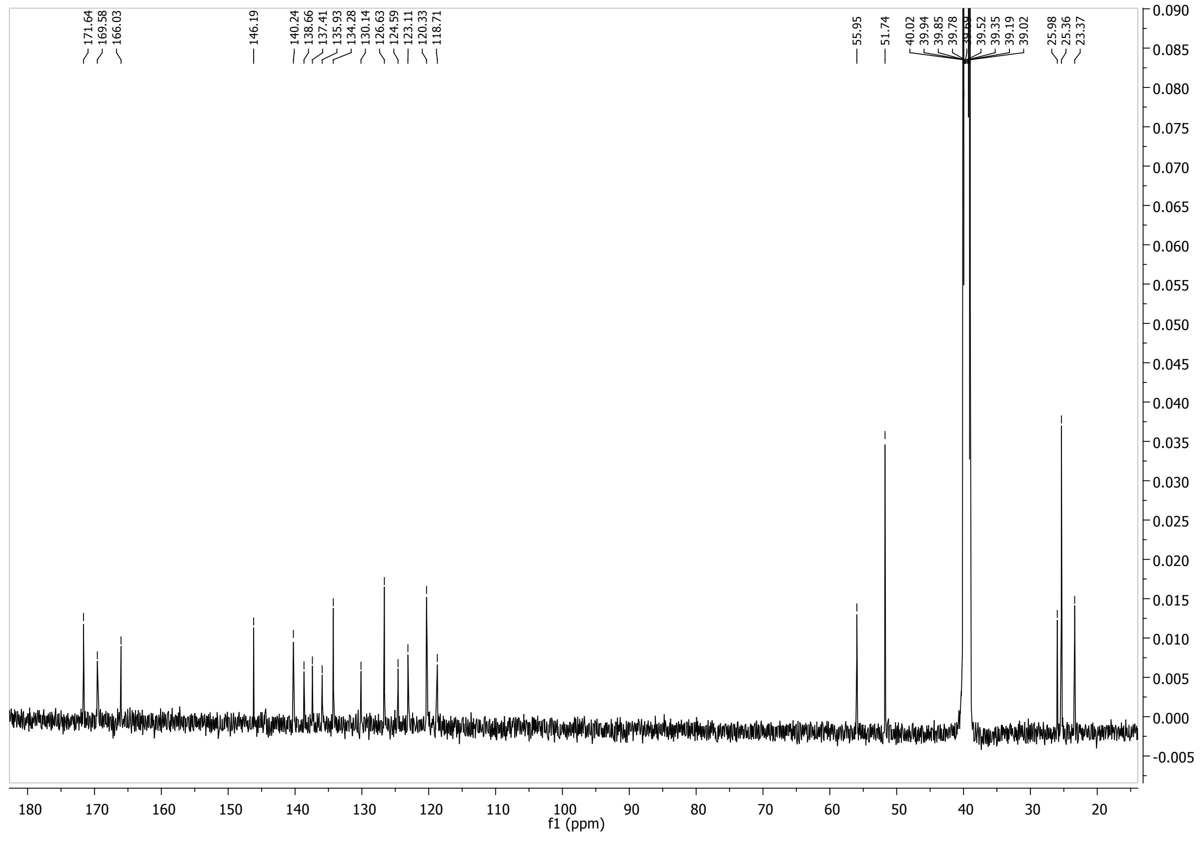

Compound **2** (DMSO-d6)

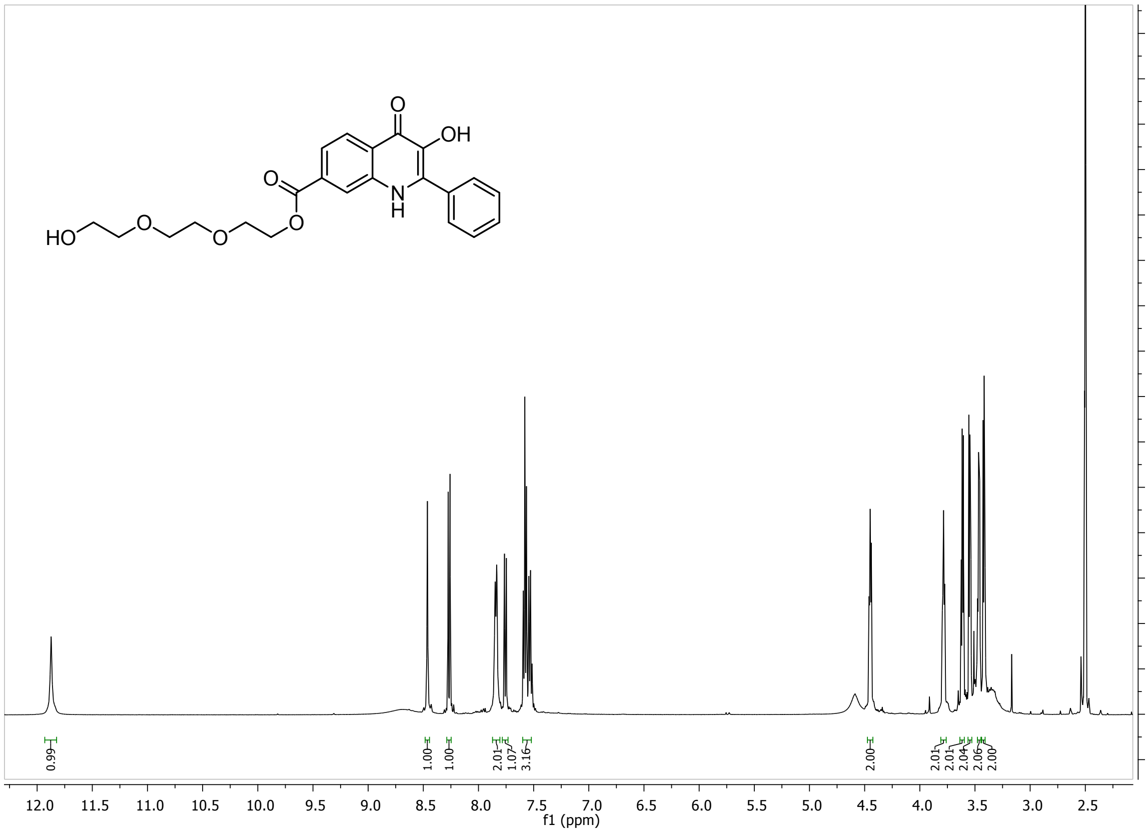

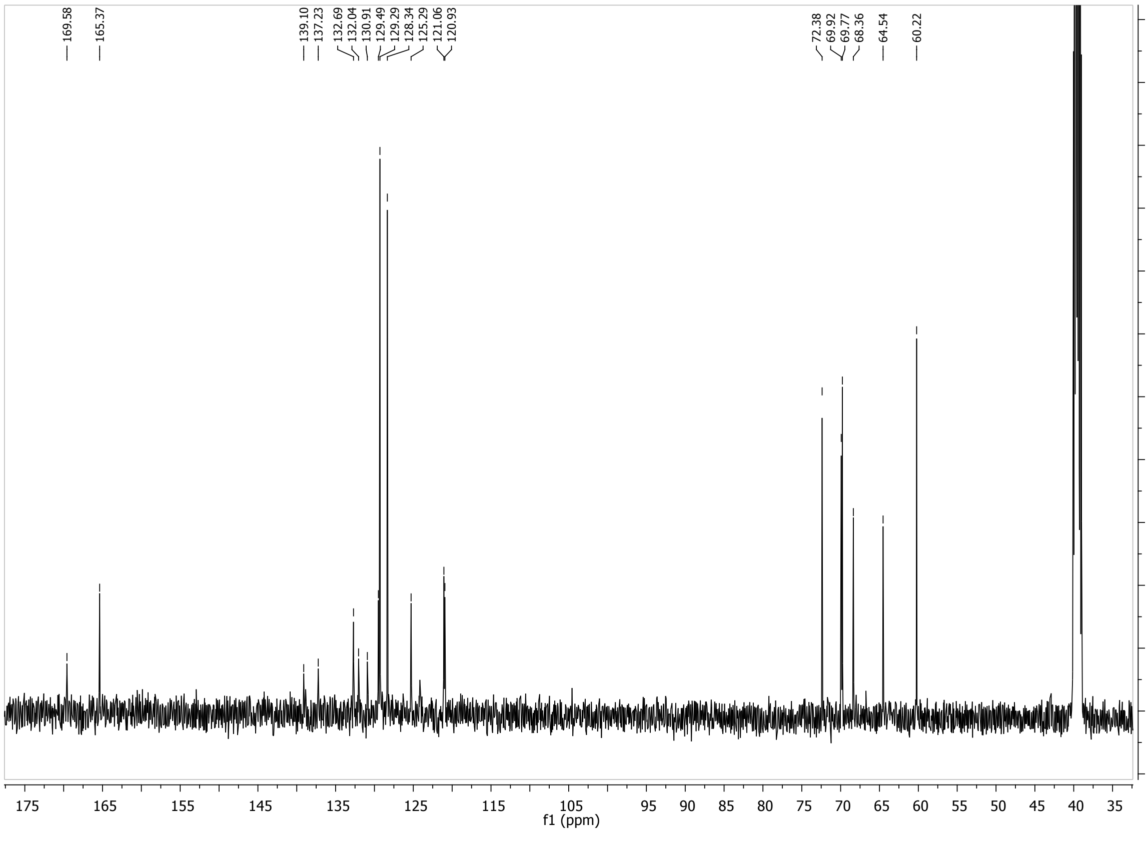

Compound **4** ($CDCl_3$/DMSO-d6)

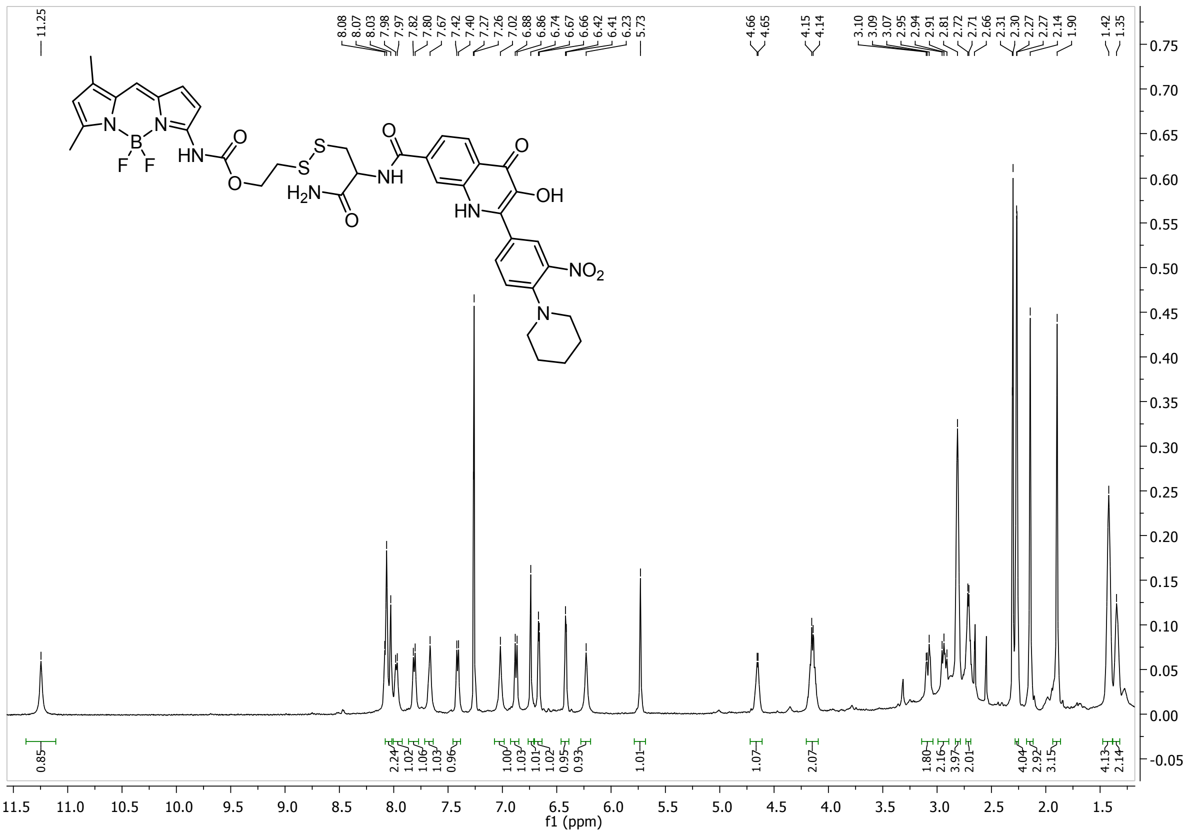

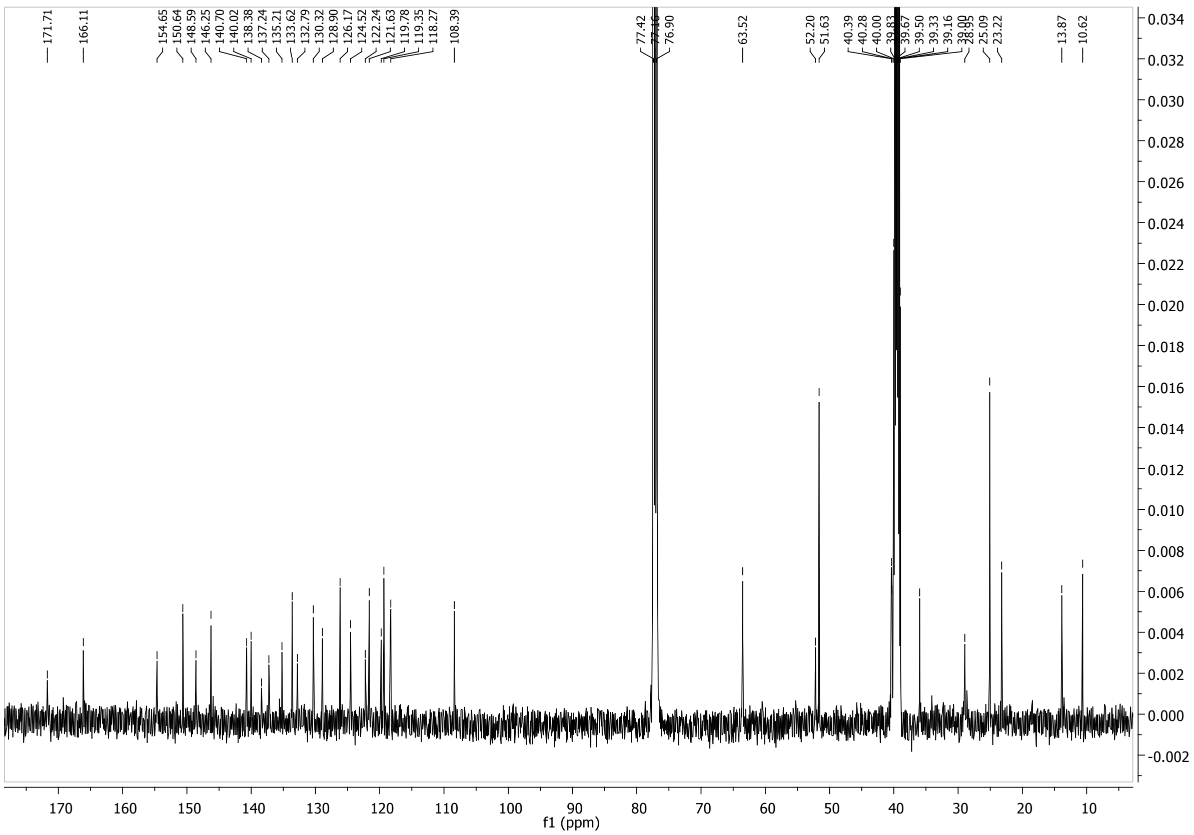

Compound **5** (DMSO-d6)

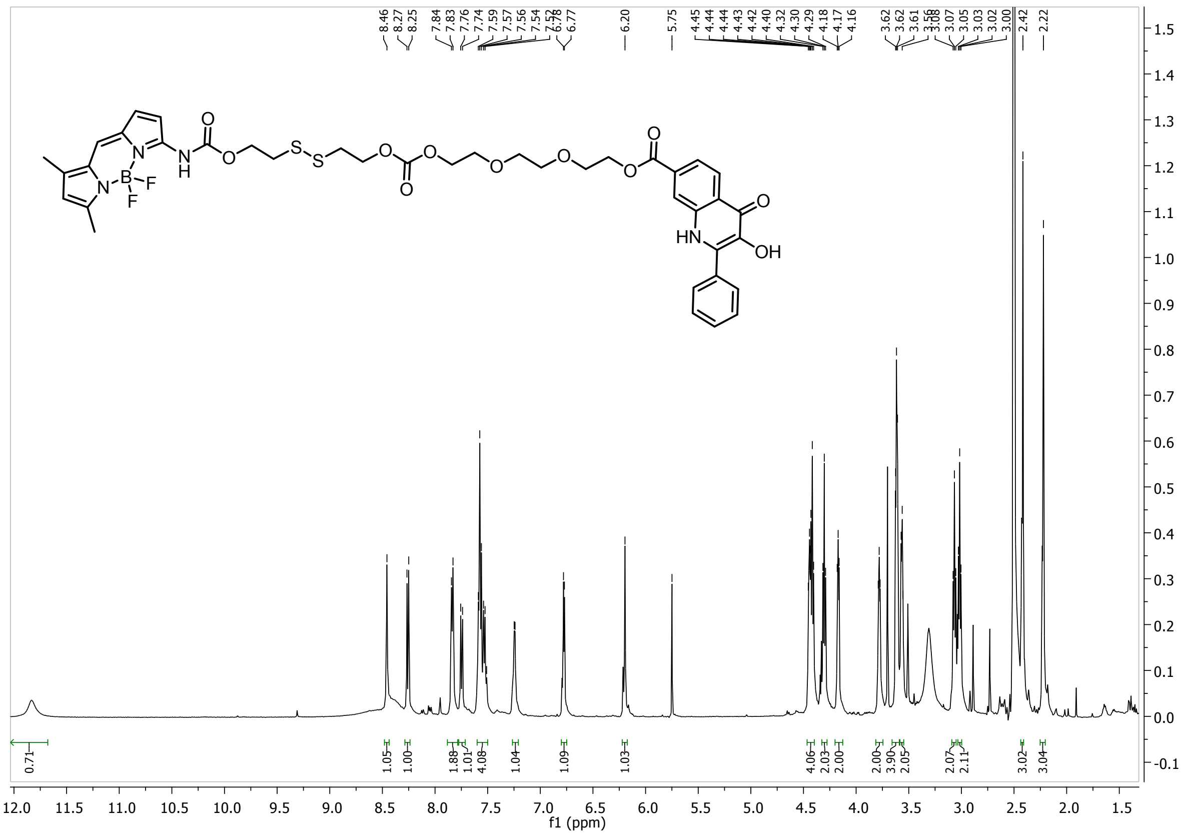

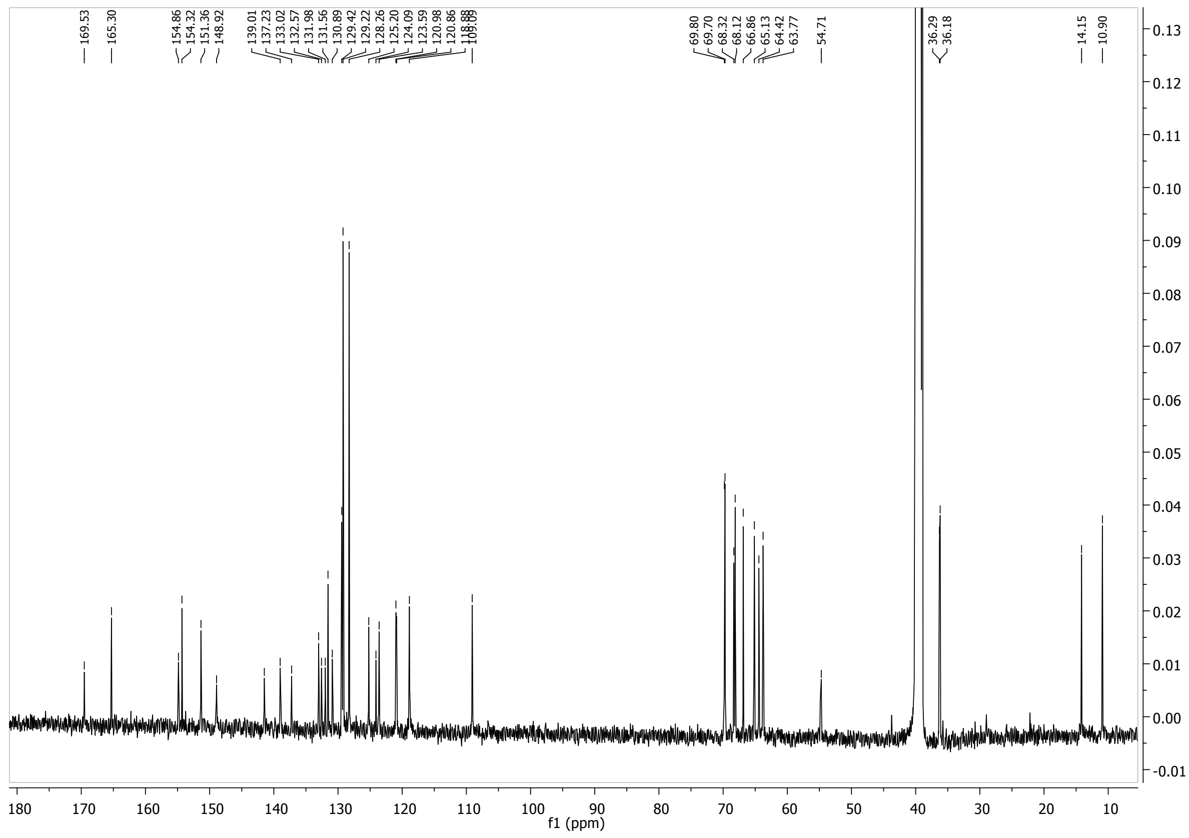

Compound **6** (DMSO-d6)

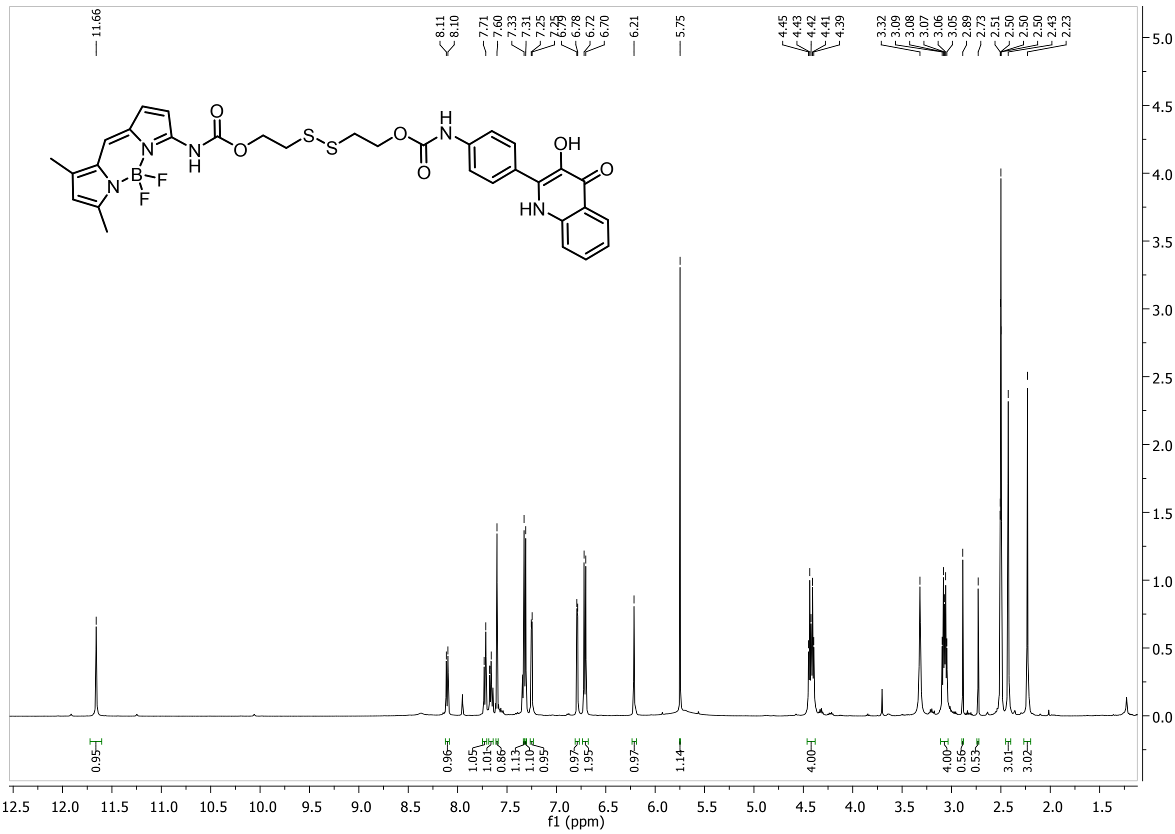

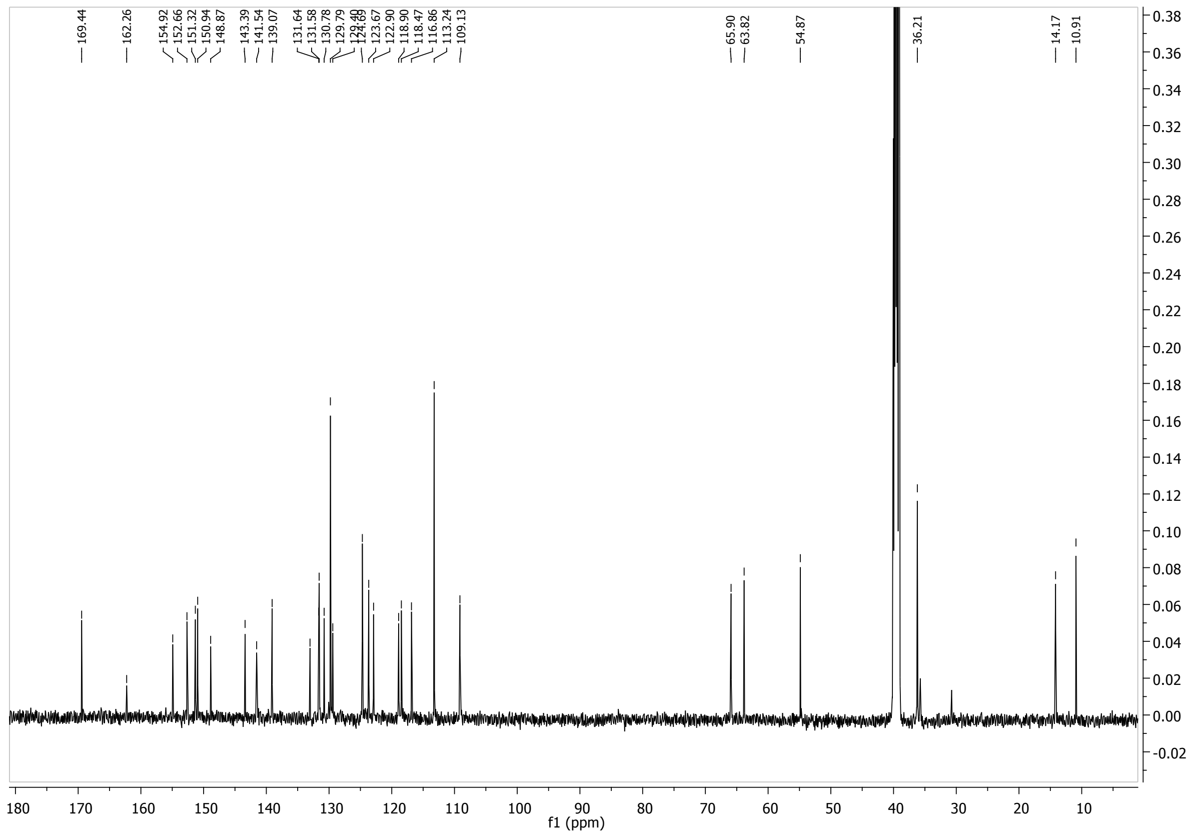

Compound **7** ($CDCl_3$)

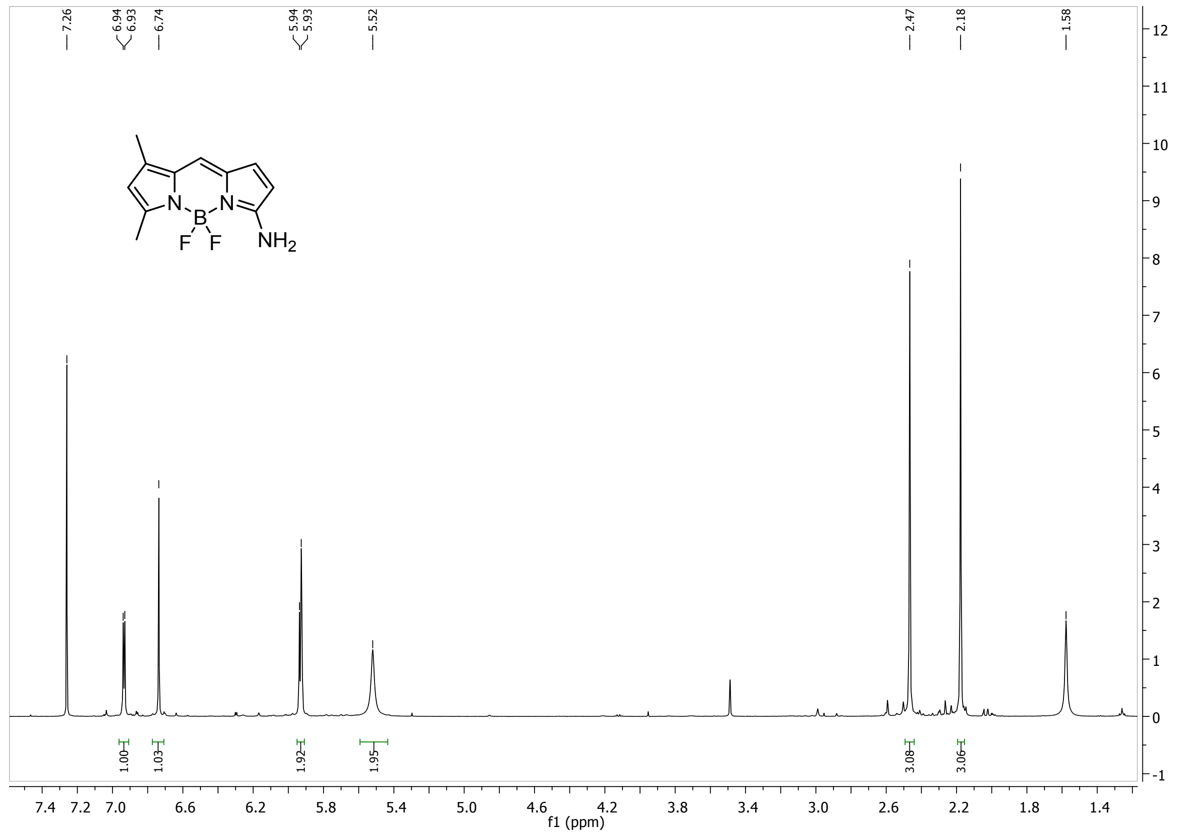

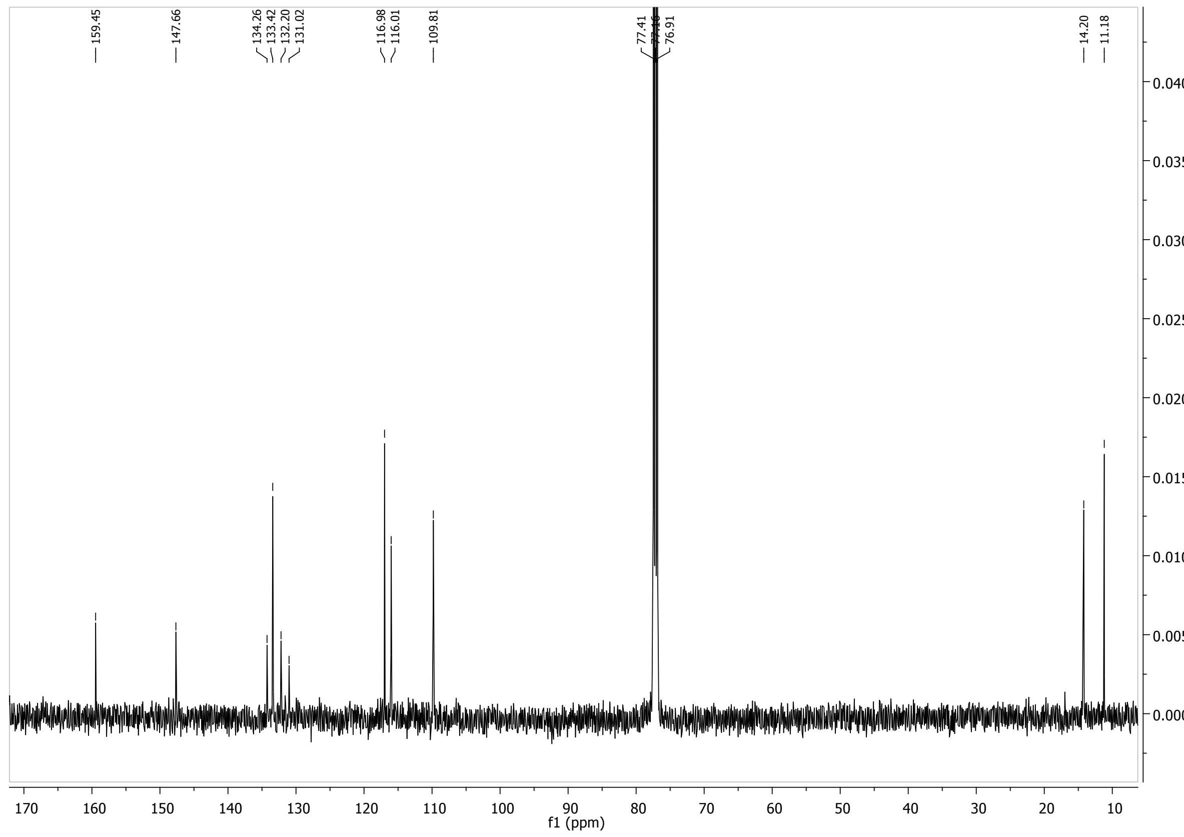

Compound **8** (DMSO-d6)

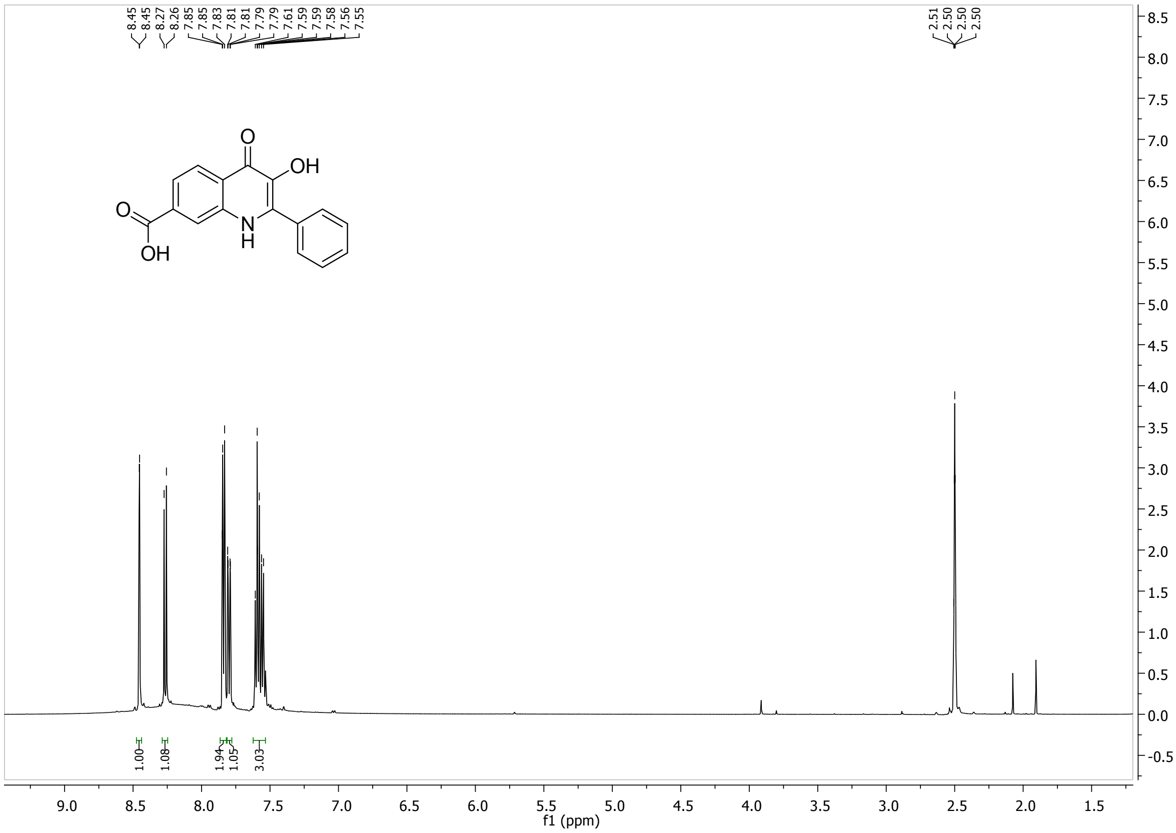

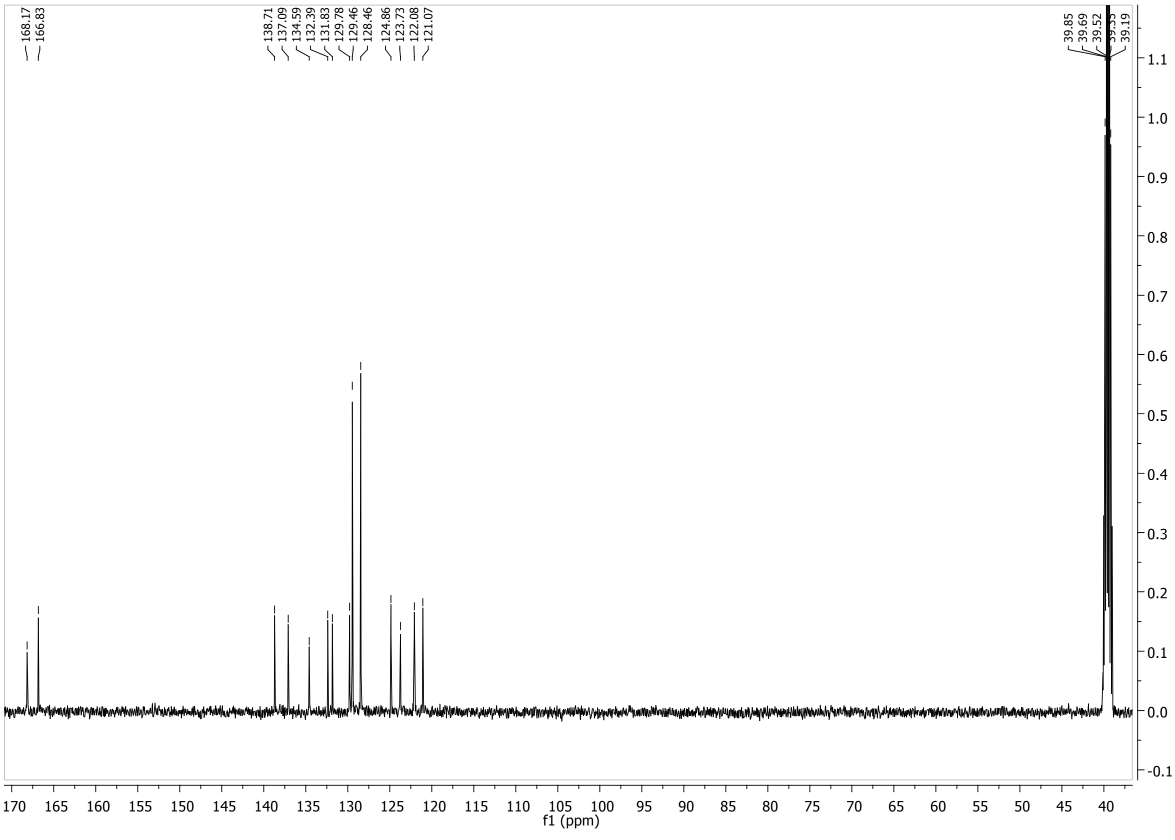

Compound **9** (DMSO-d6)

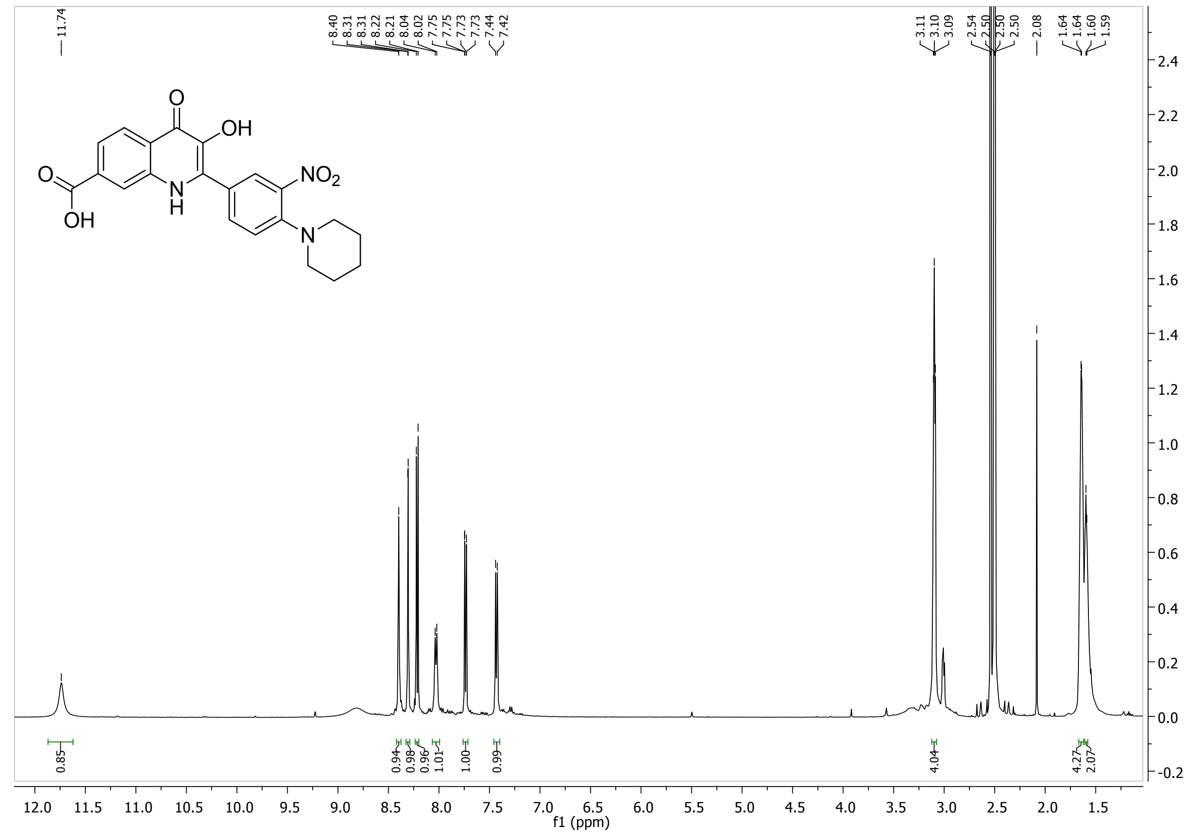

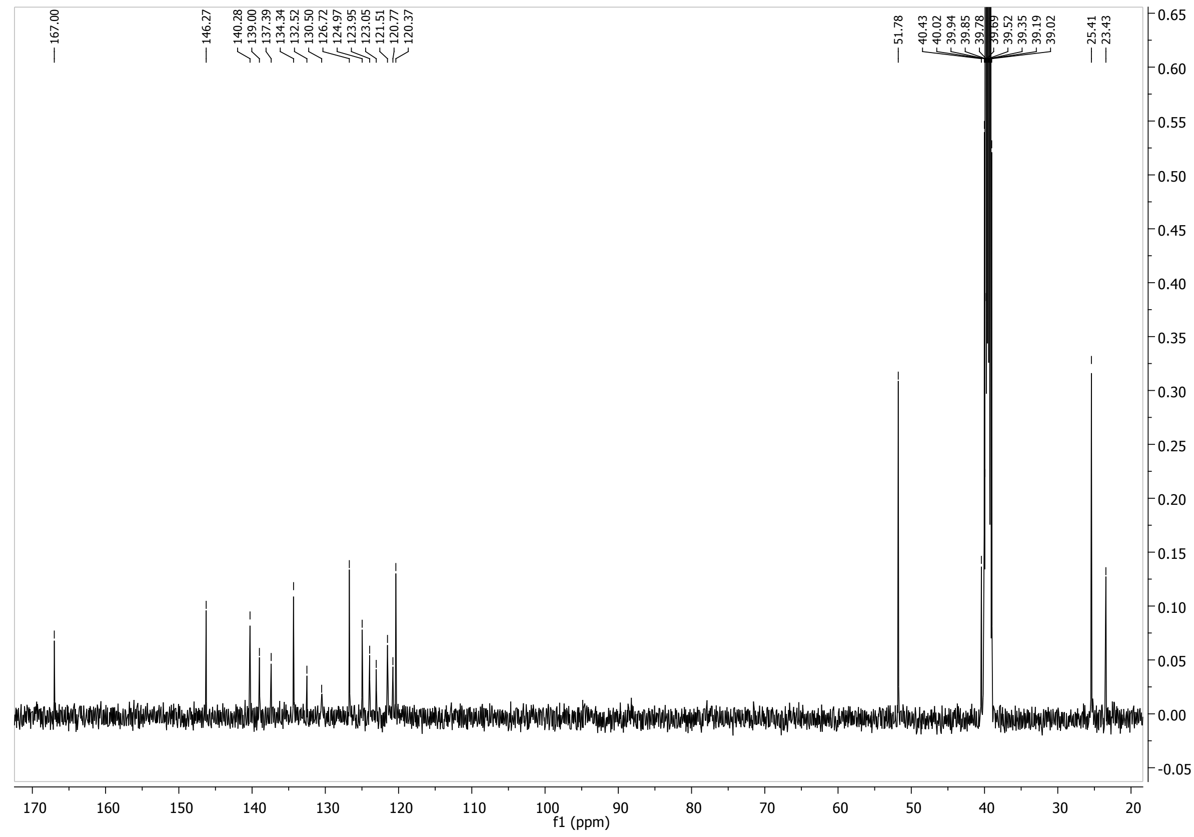

Compound **12** ($CDCl_3$)

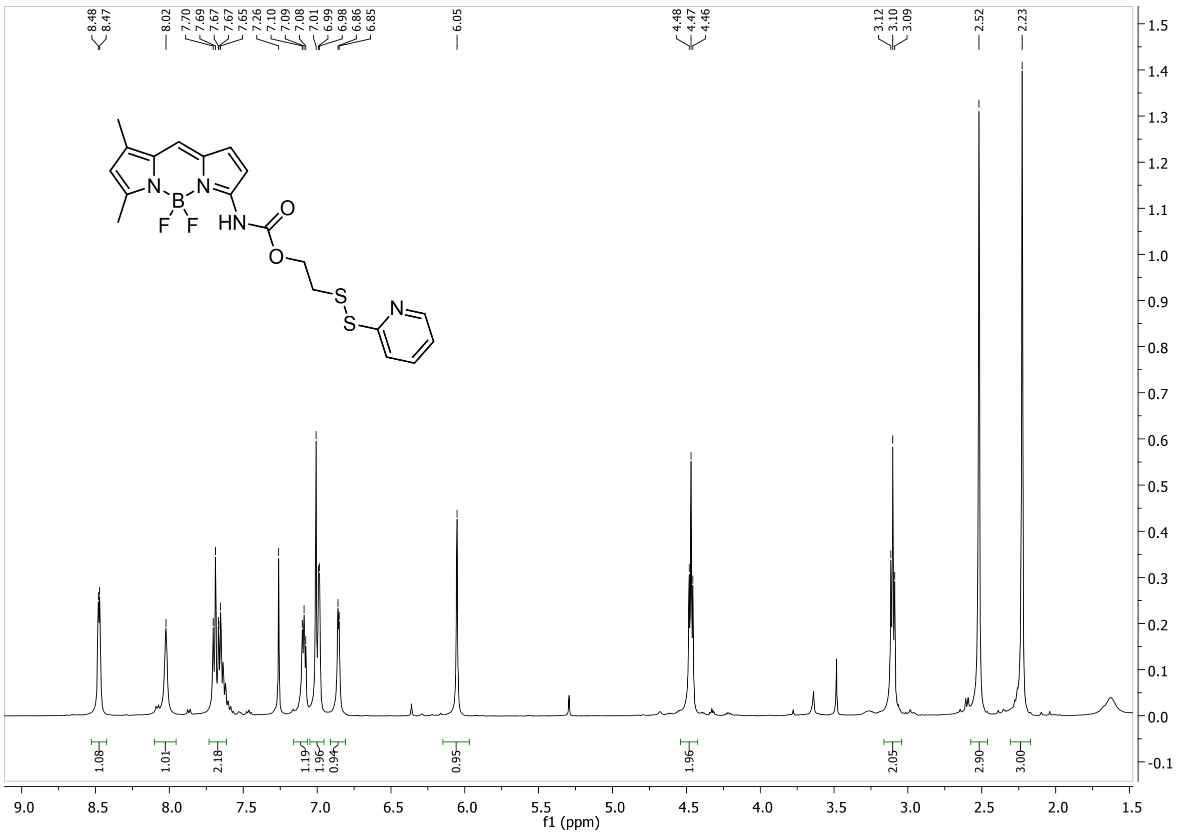

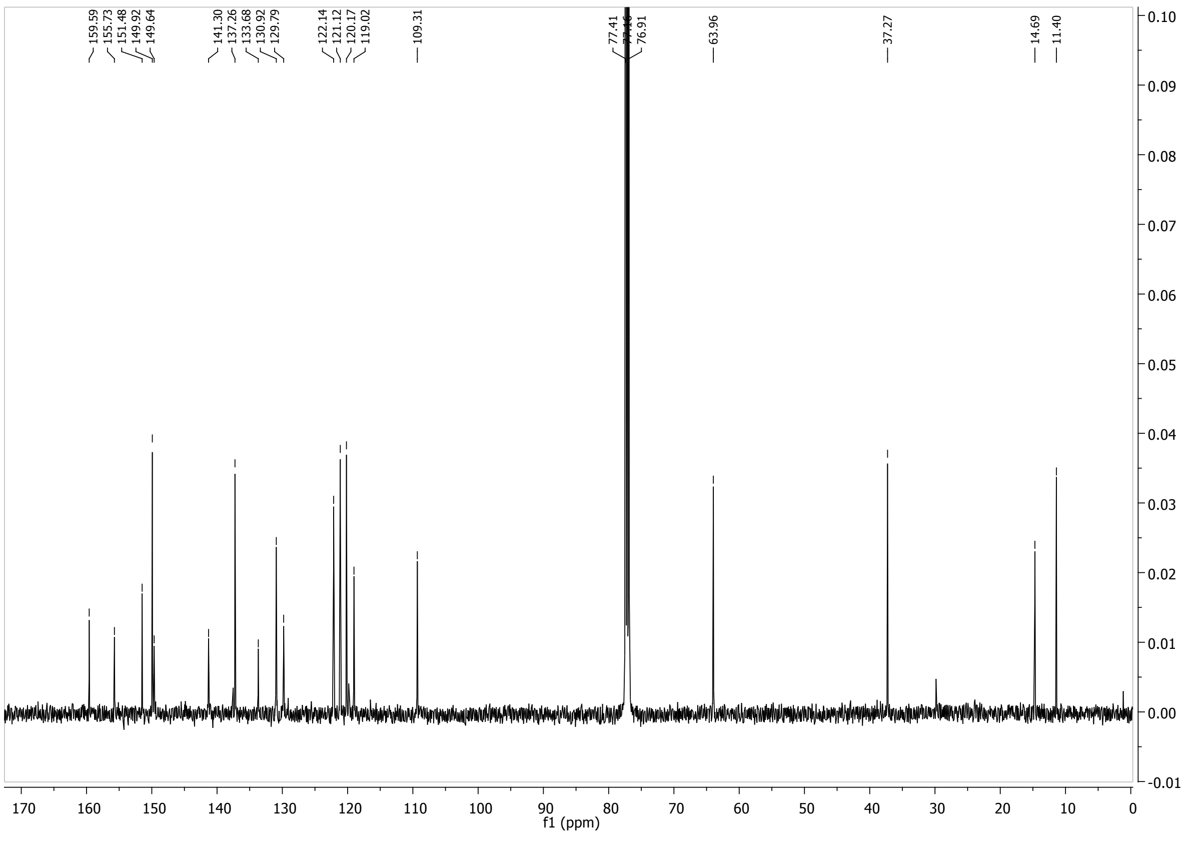

Compound **13** (CDCl3)

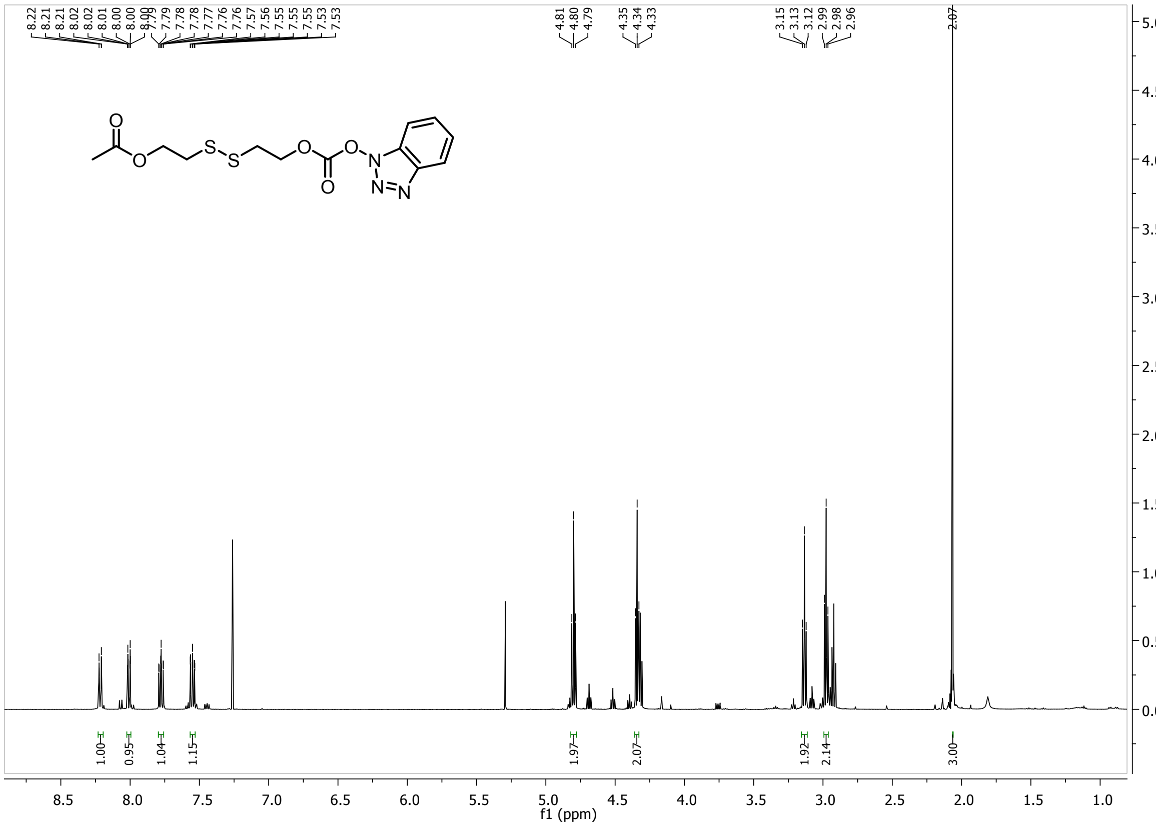

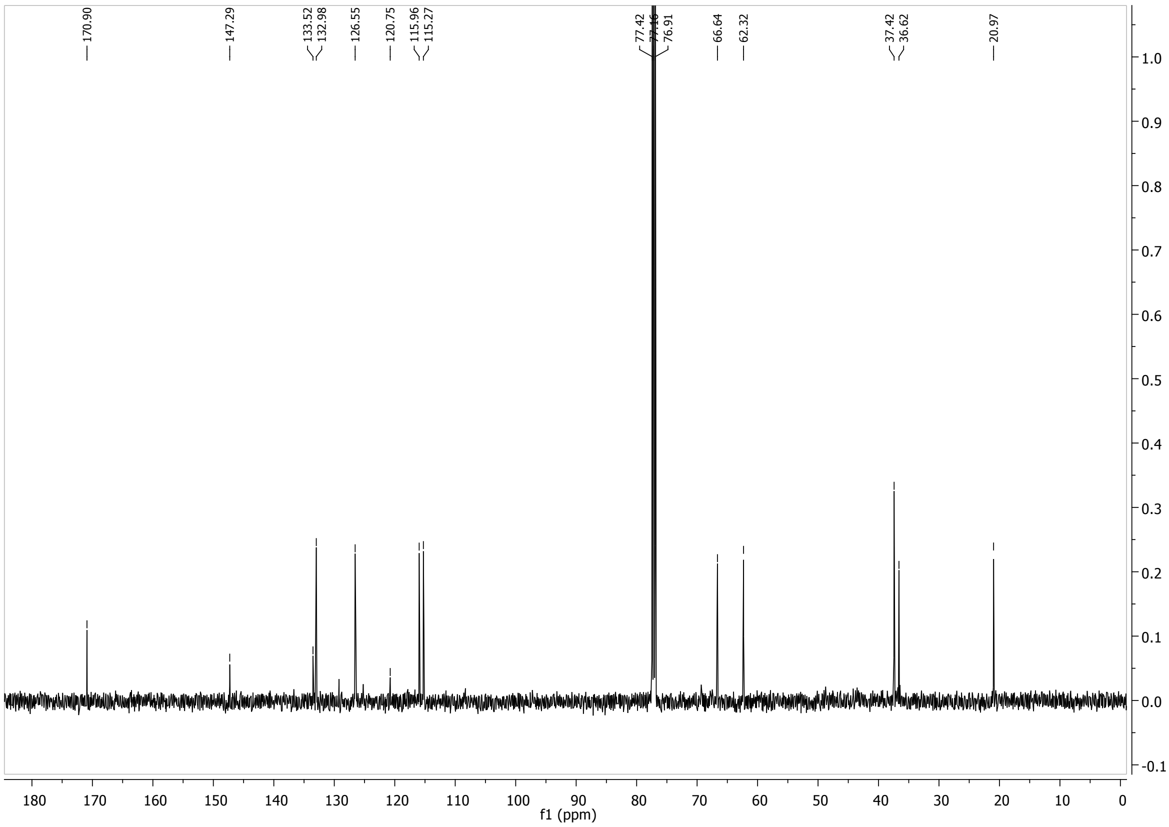

Compound **14** ($CDCl_3$)

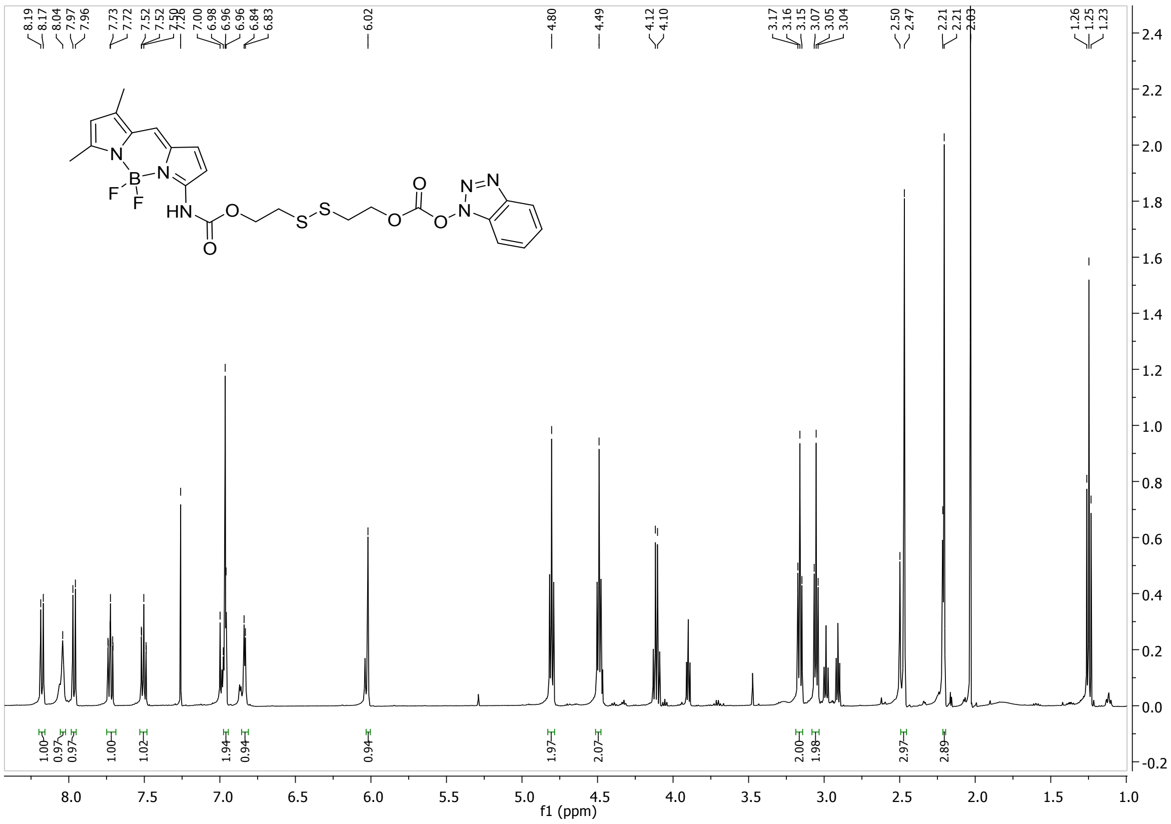

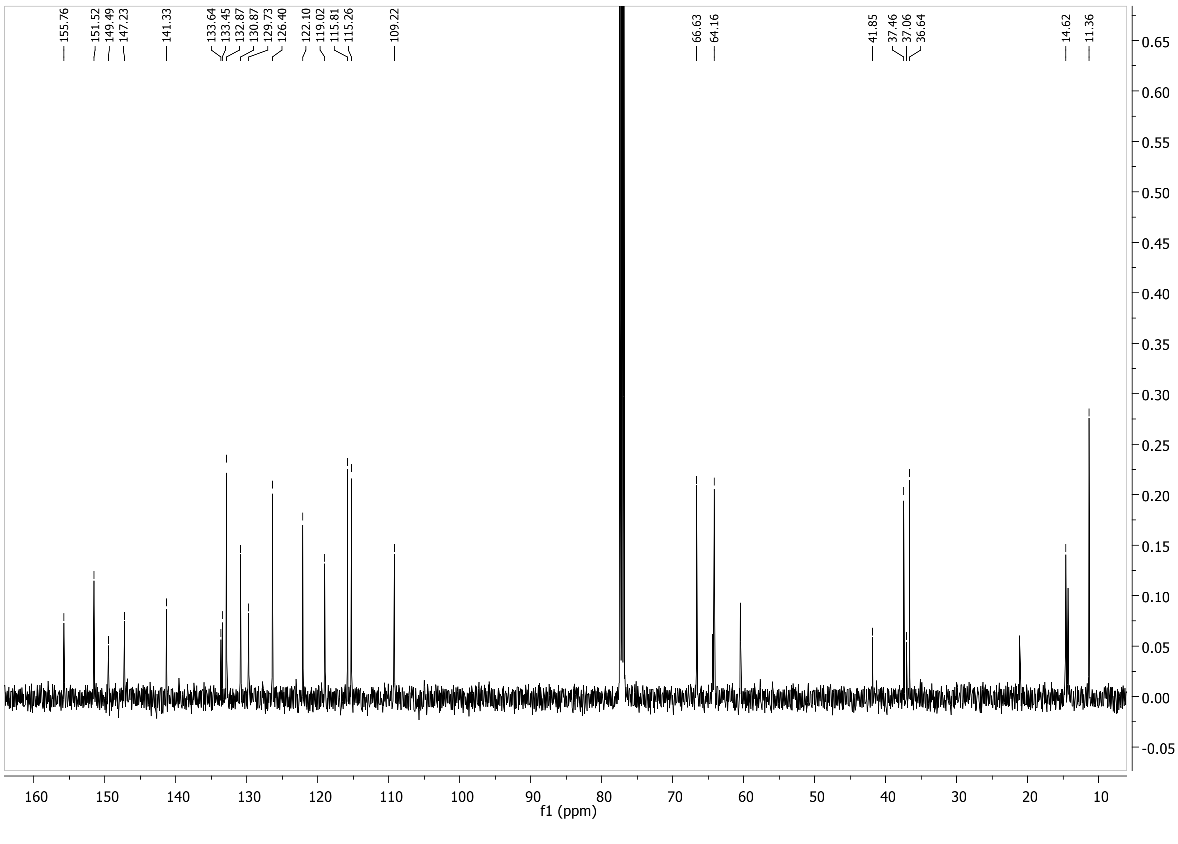

Compound **18** (DMSO-d6)

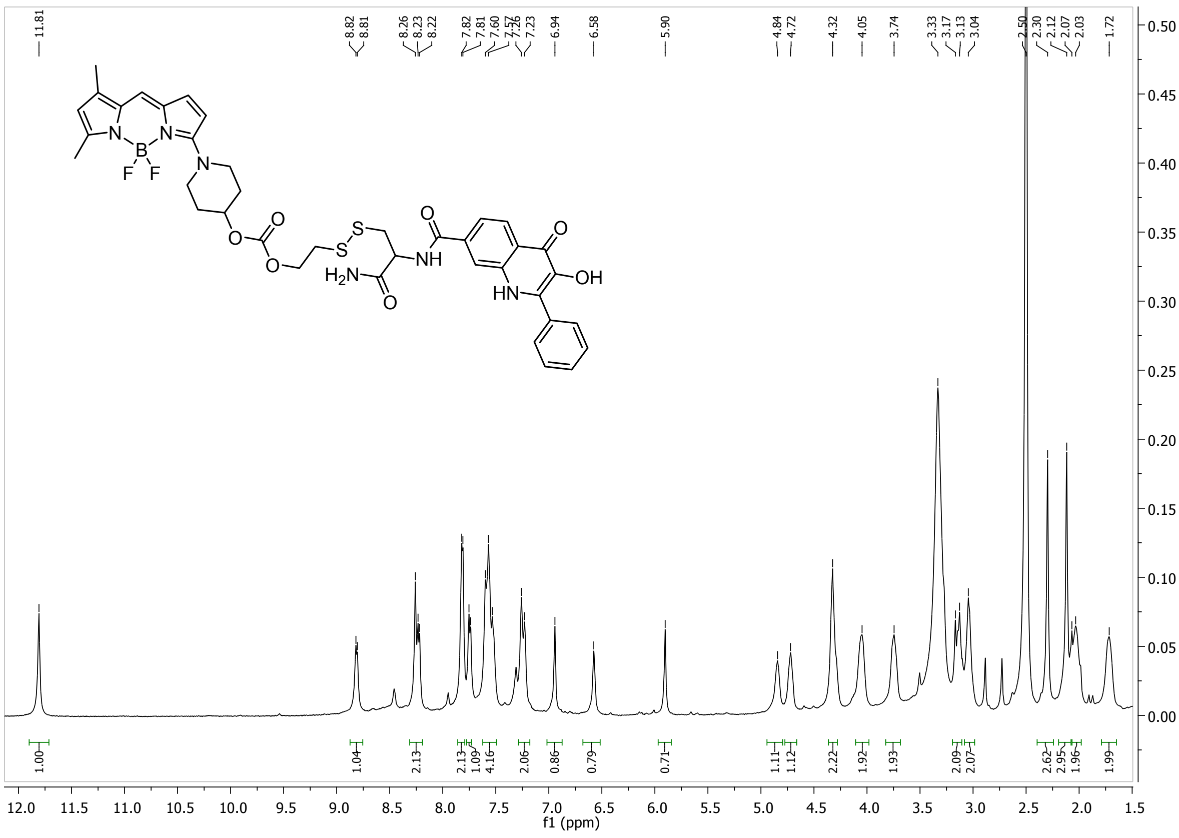

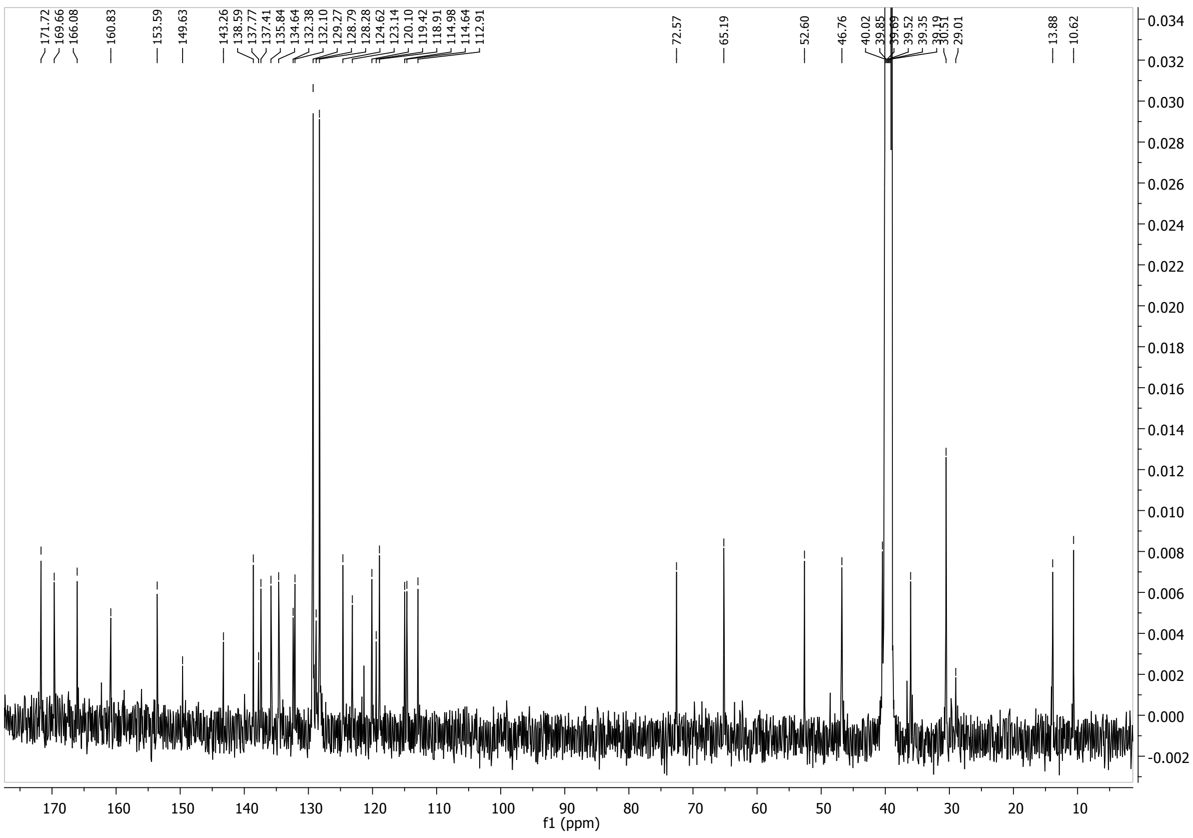

Compound **19** ($CDCl_3$)

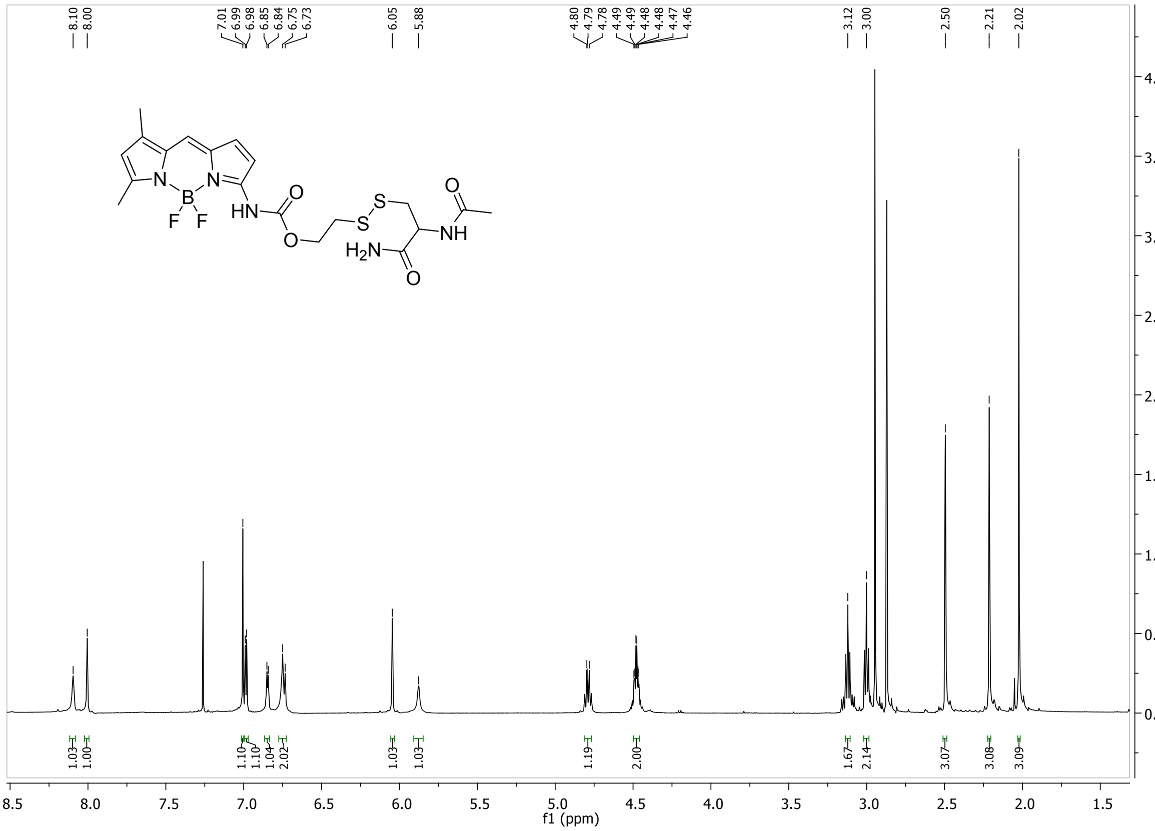

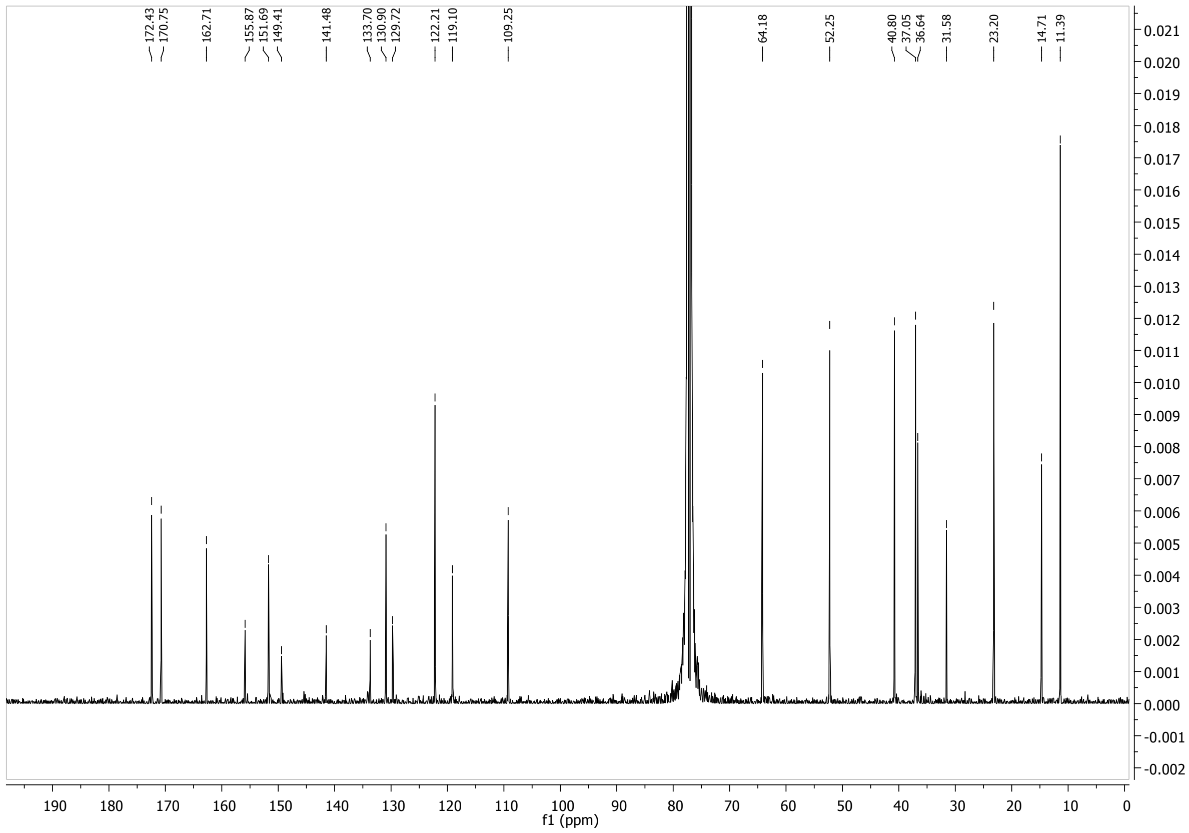

Compound **20** ($CDCl_3$)

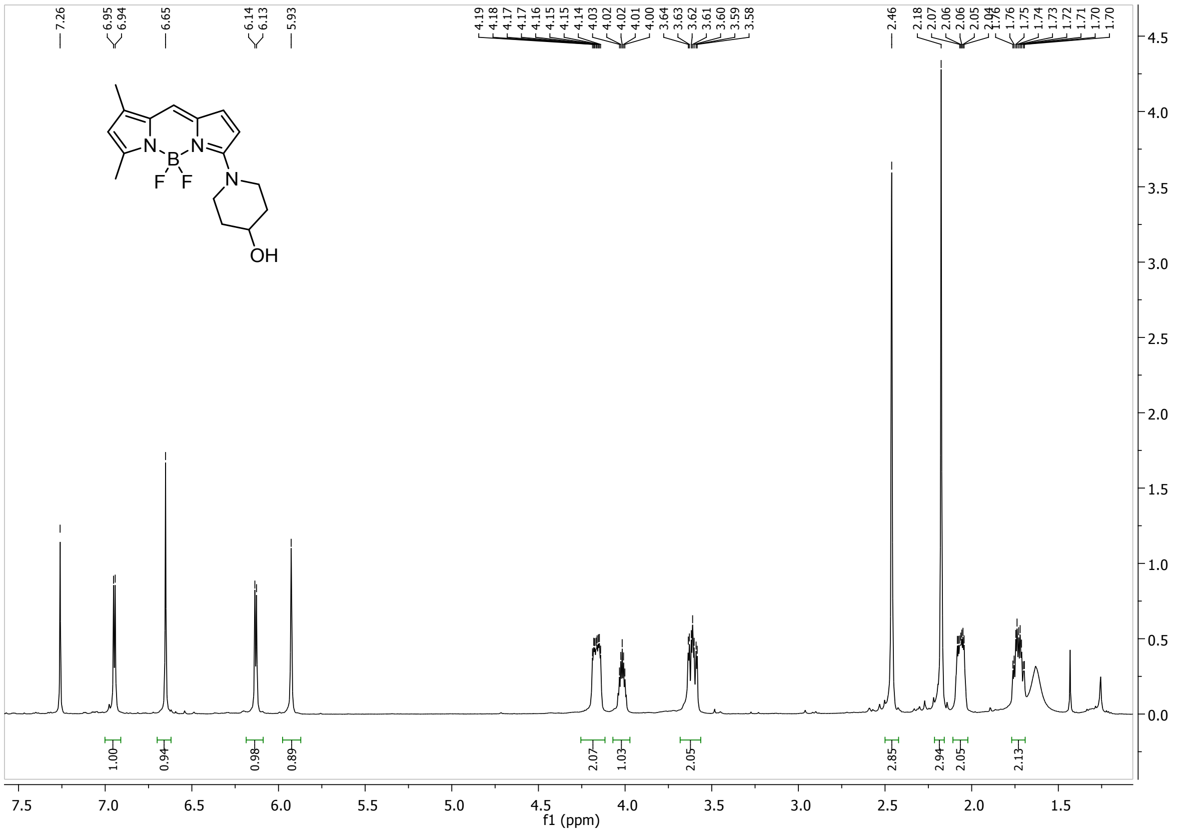

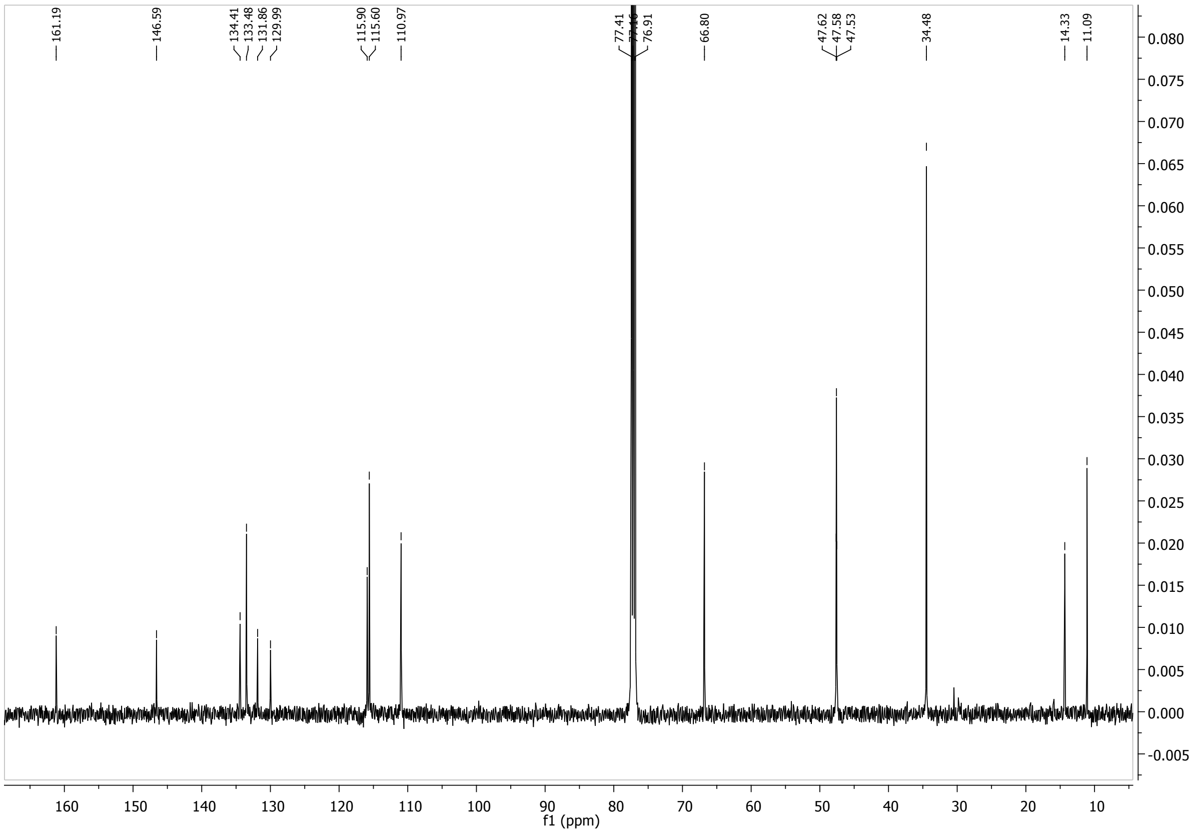

Compound **21** (DMSO-d6)

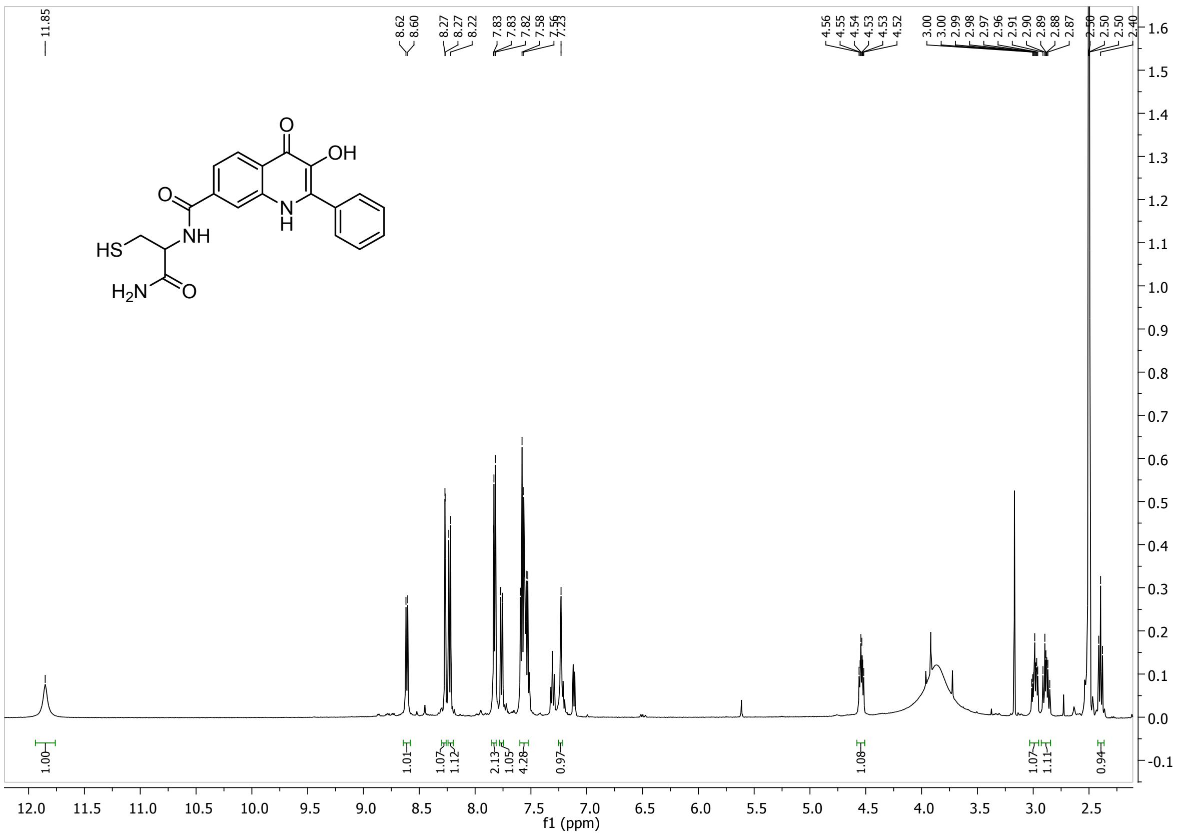

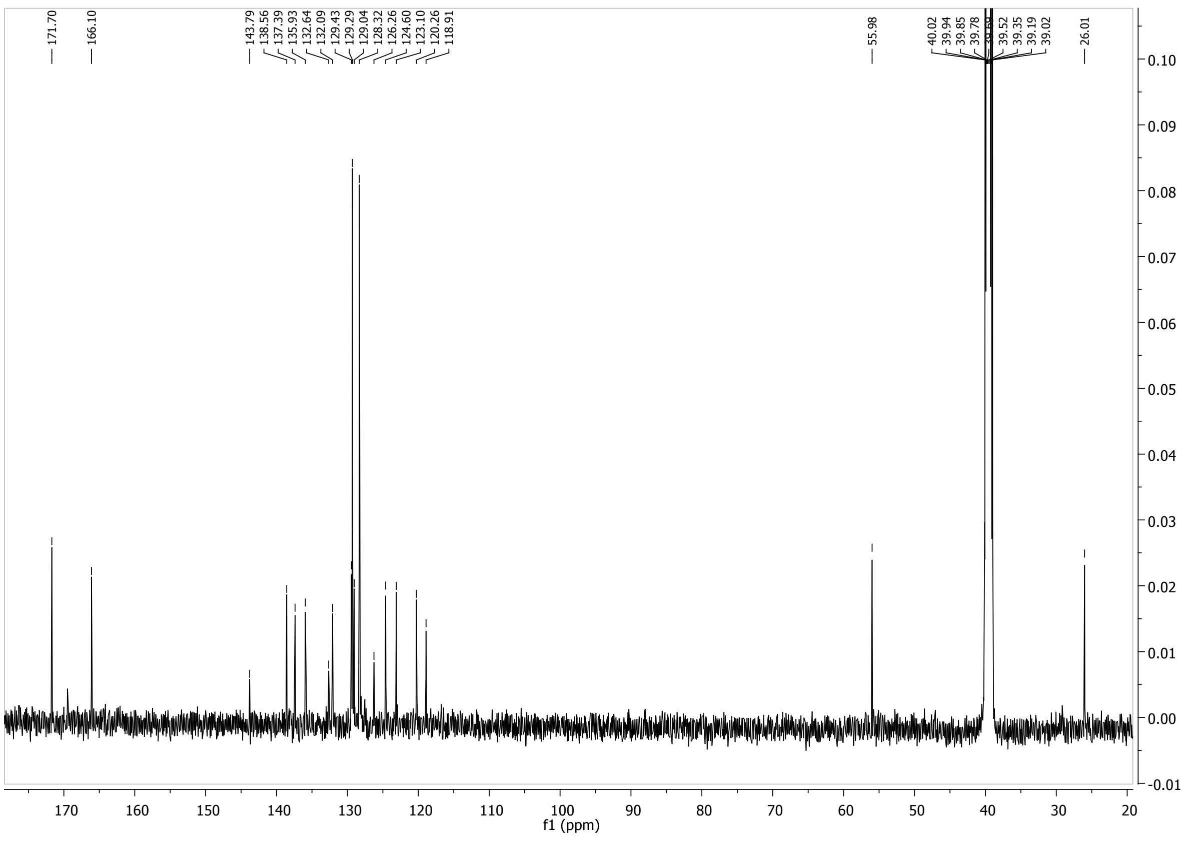

Compound **23a** ($CDCl_3$)

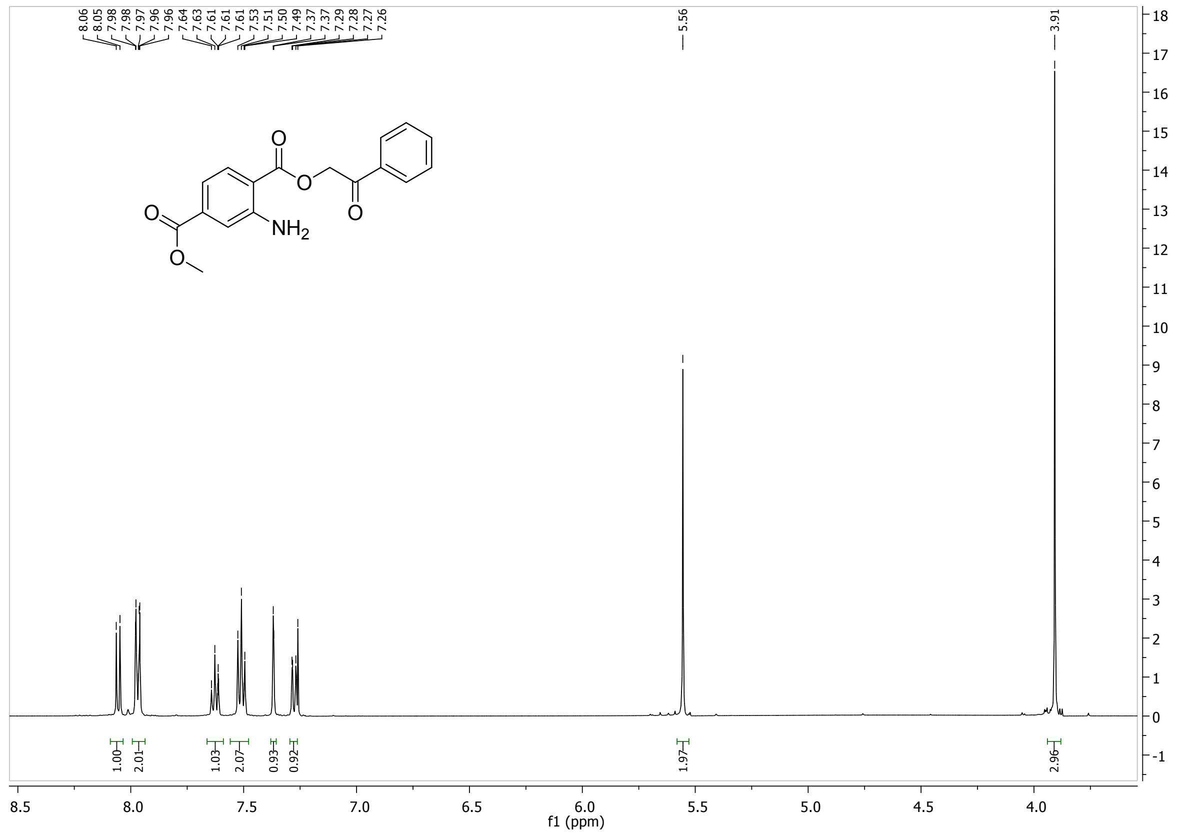

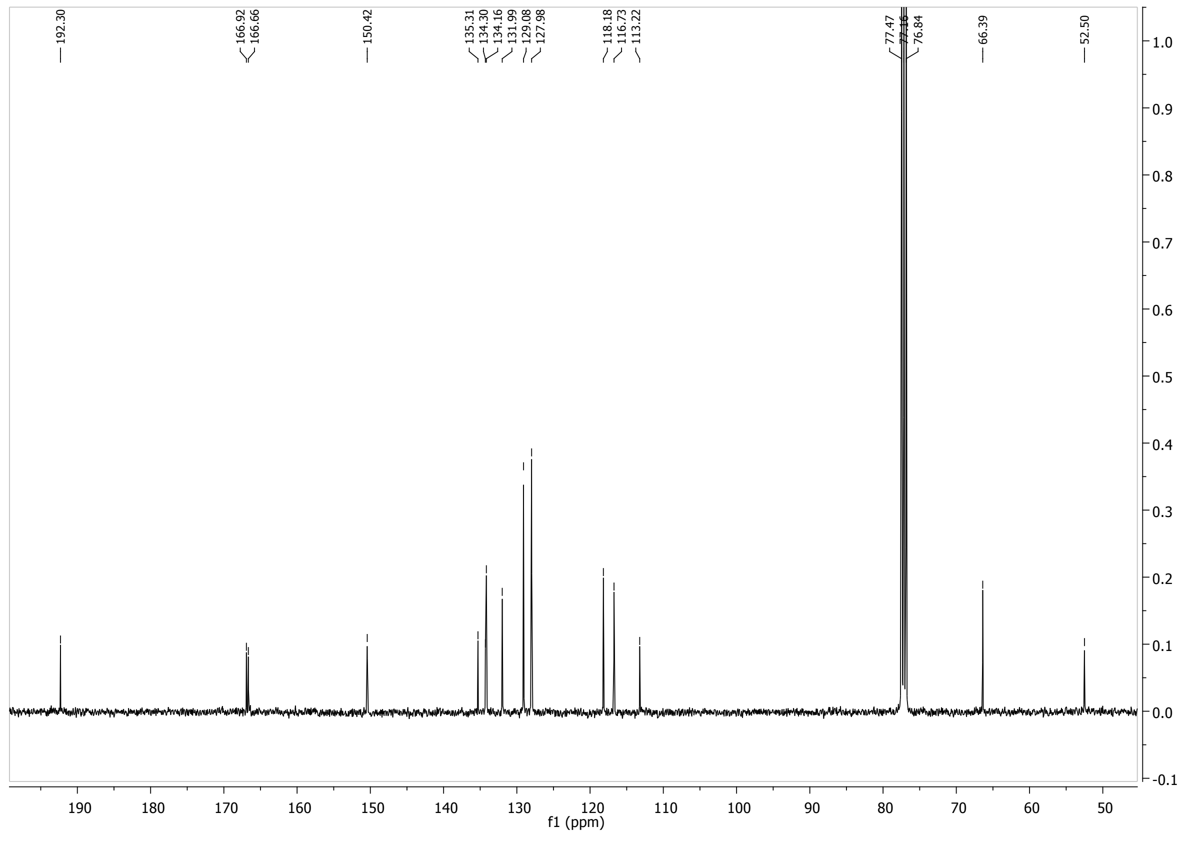

Compound **23b** (DMSO-d6)

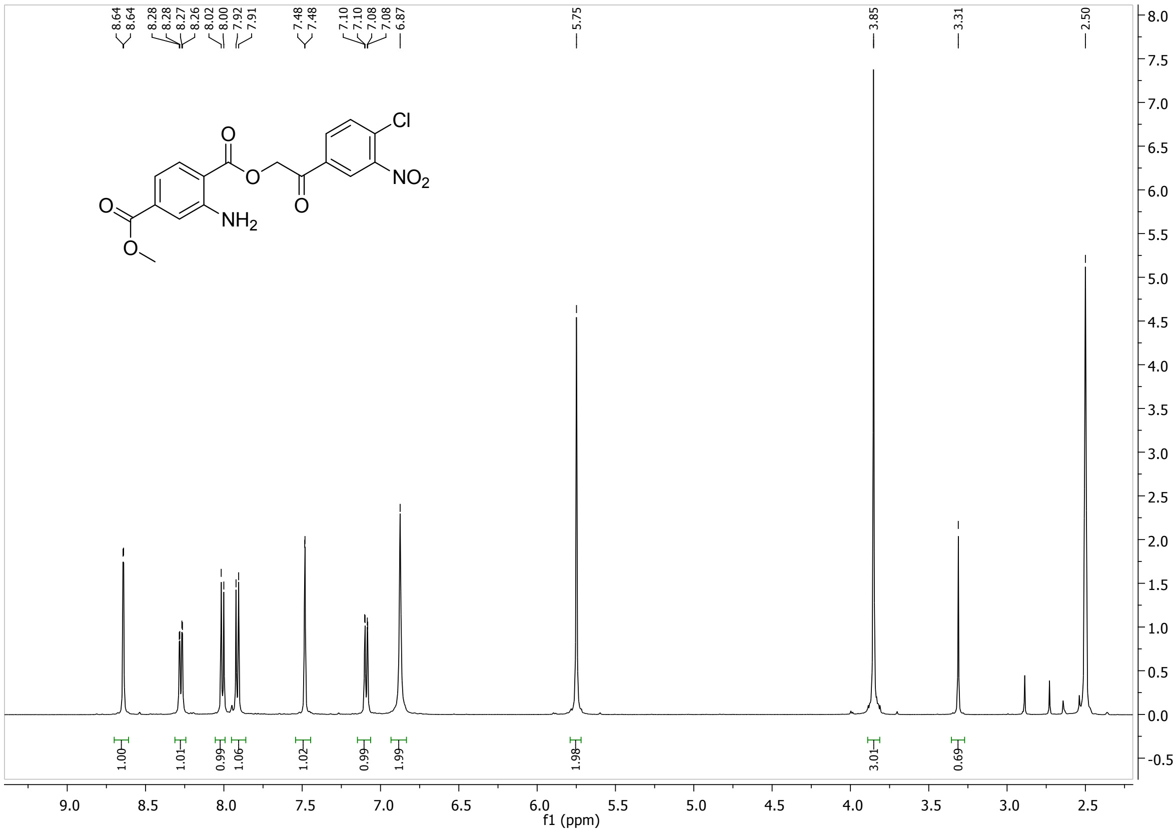

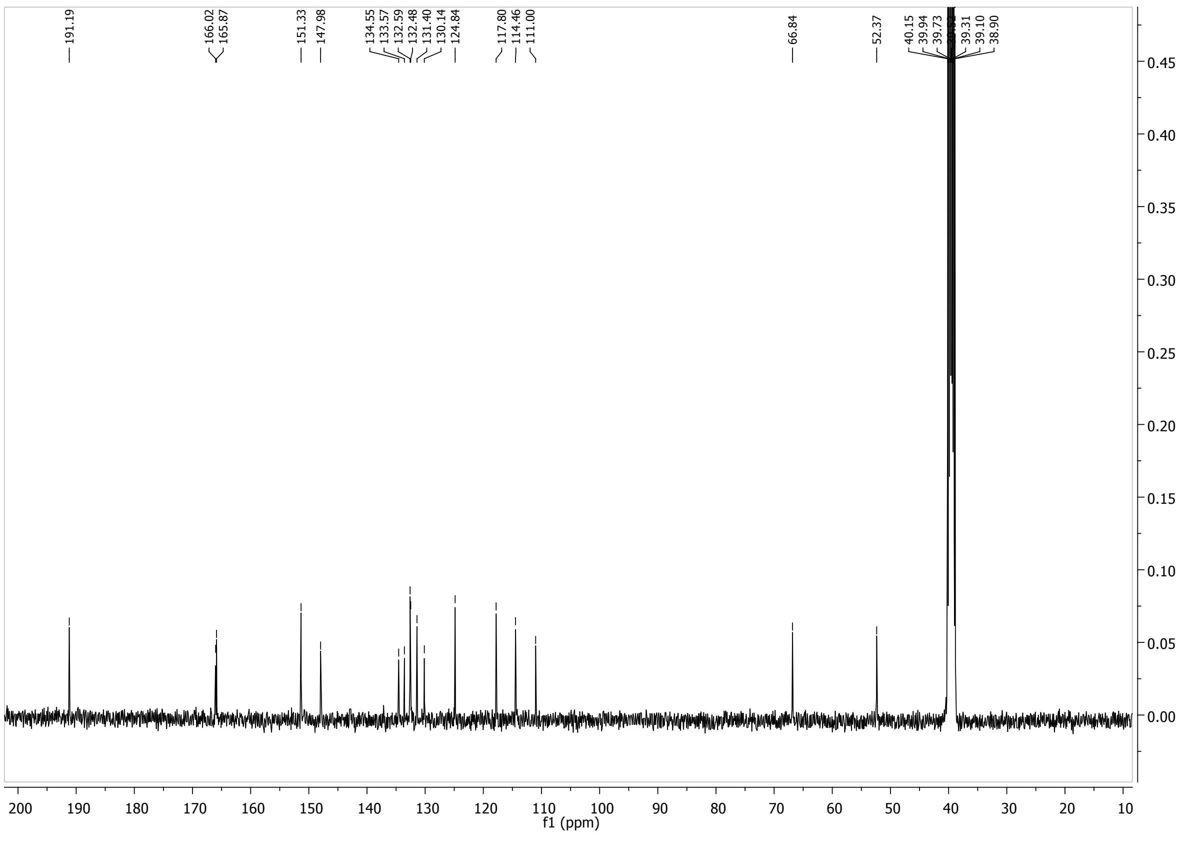

Compound **24** (DMSO-d6)

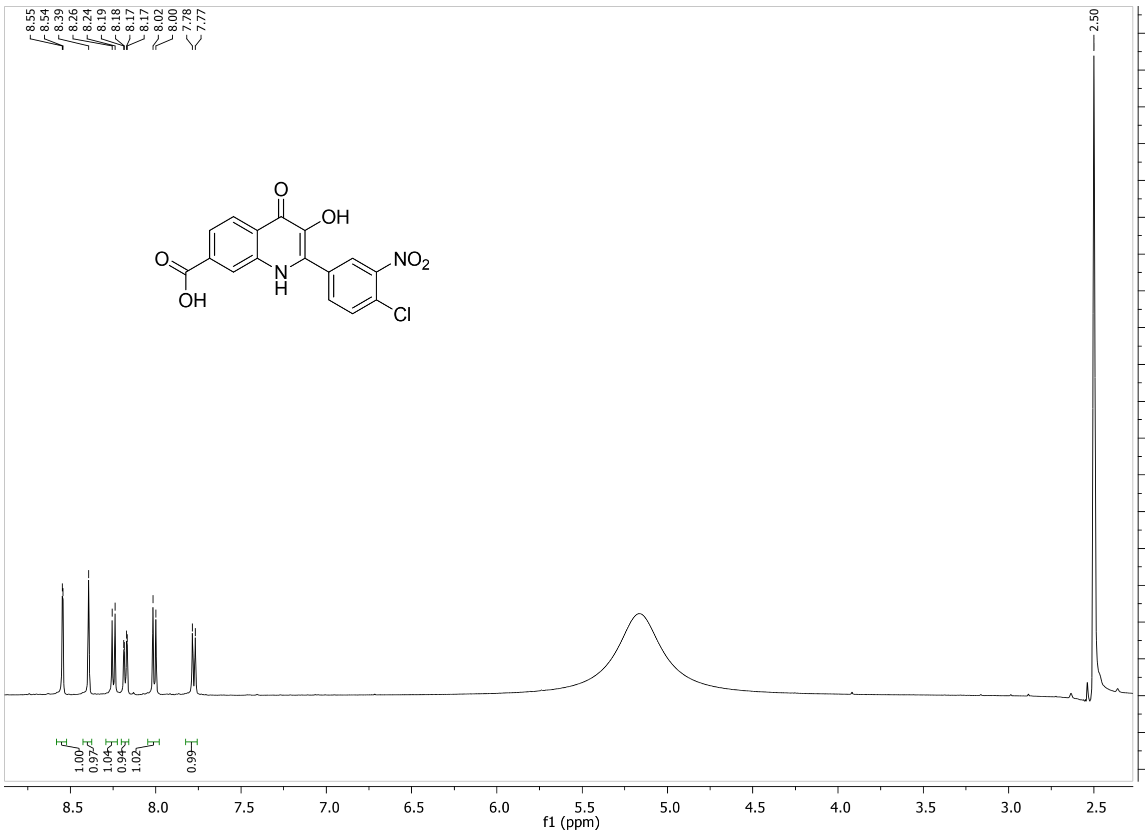

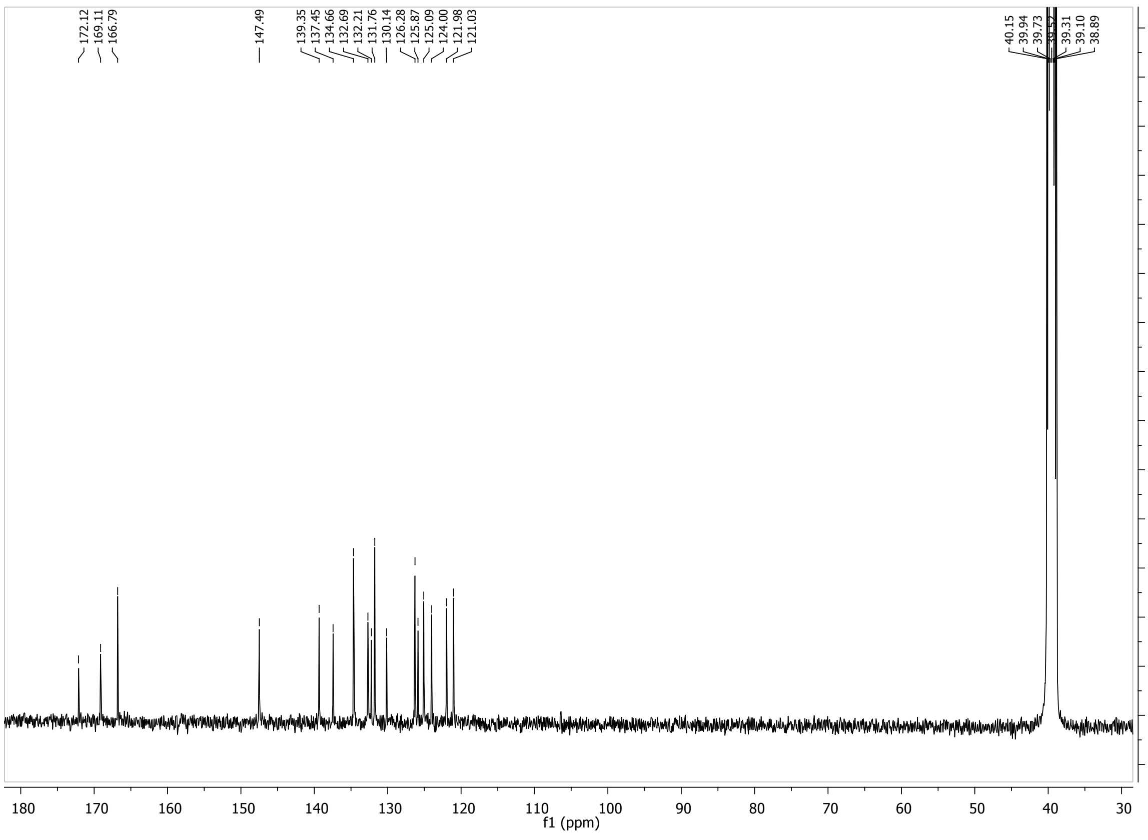

Compound **28** ($CDCl_3$)

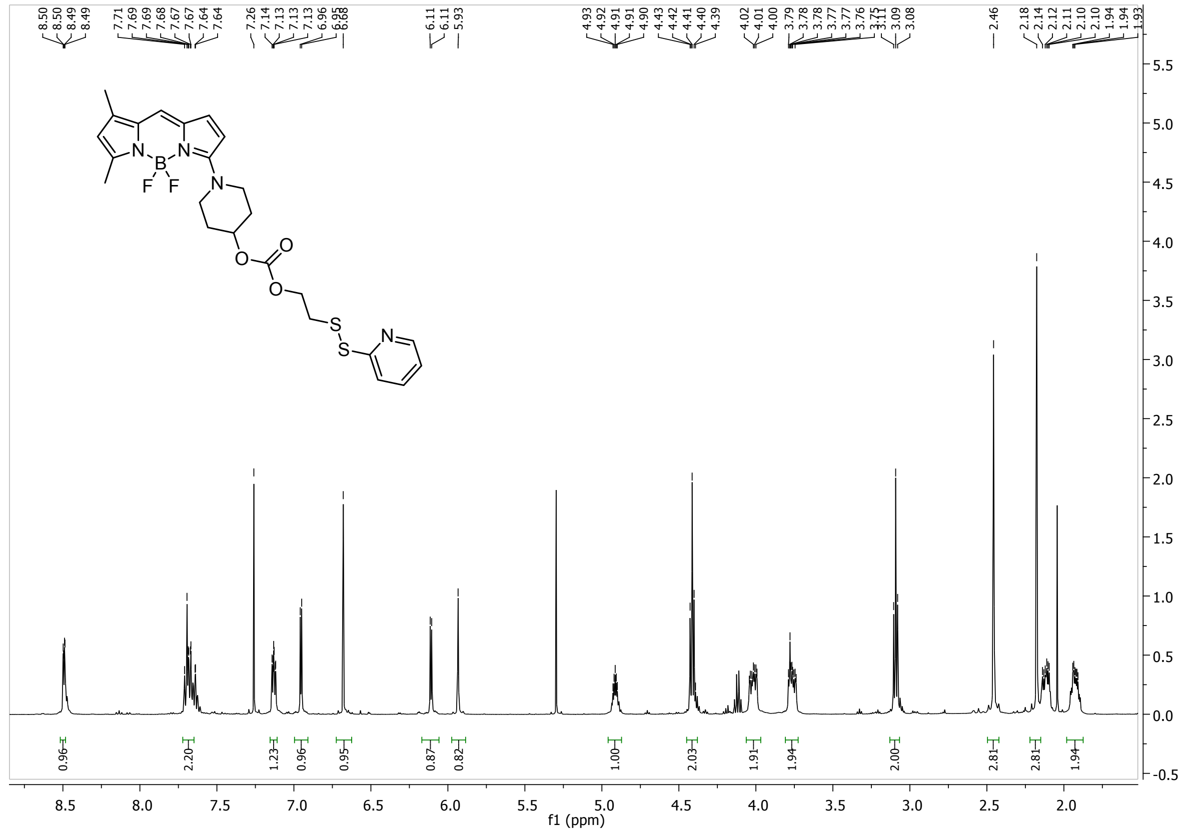

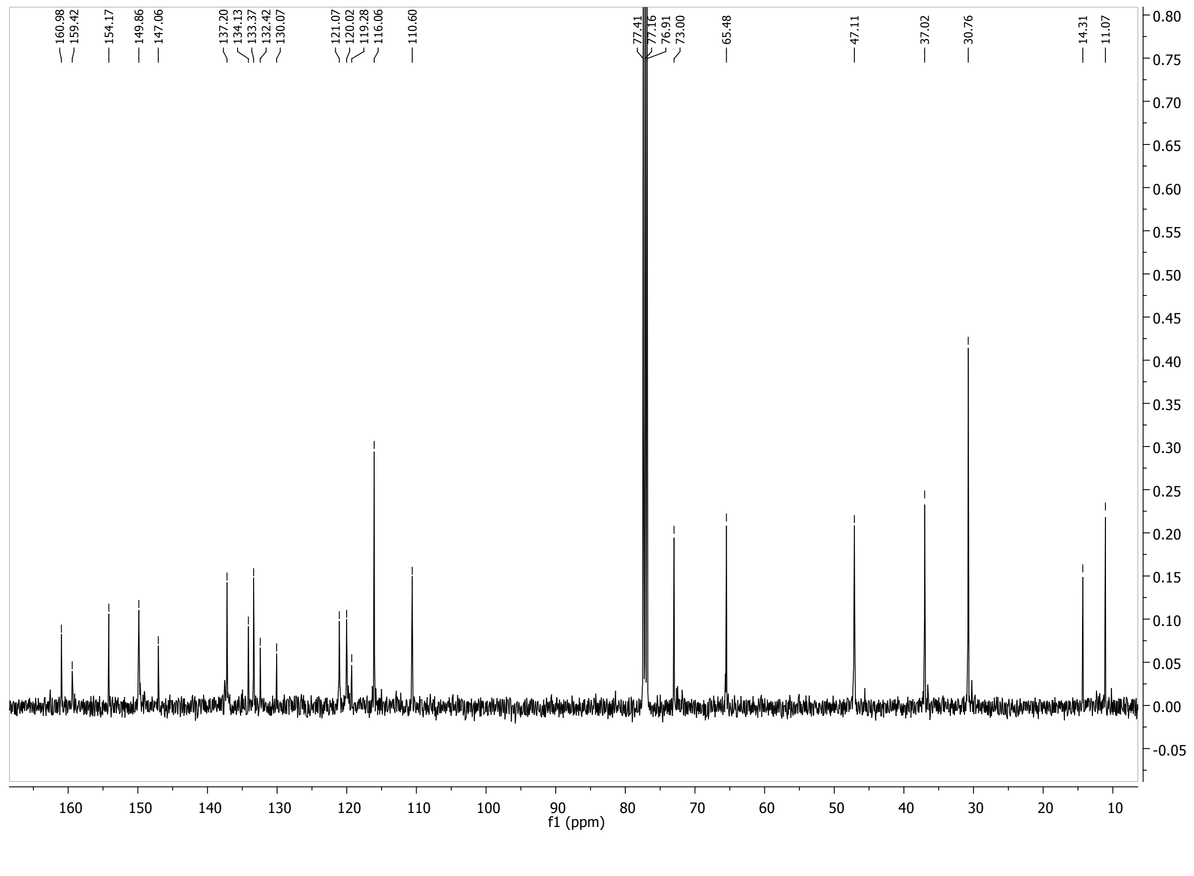

Compound **30** ($CDCl_3$)

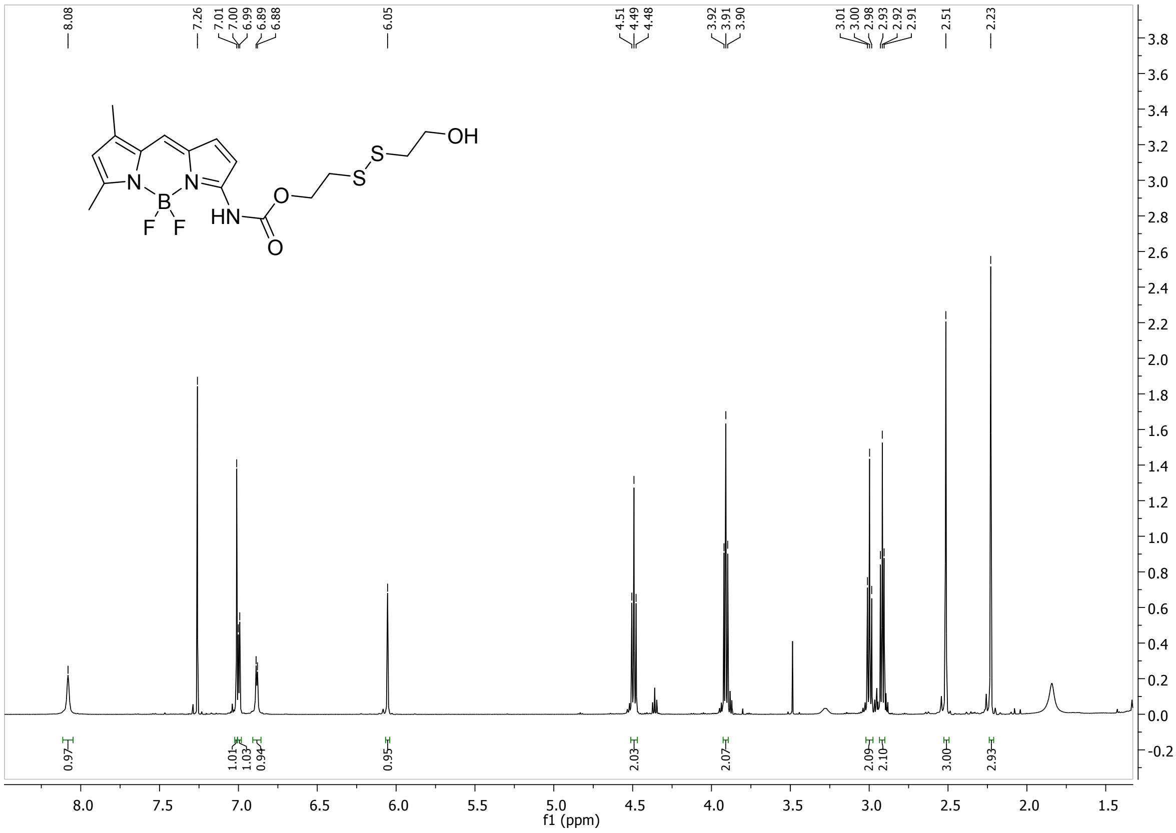

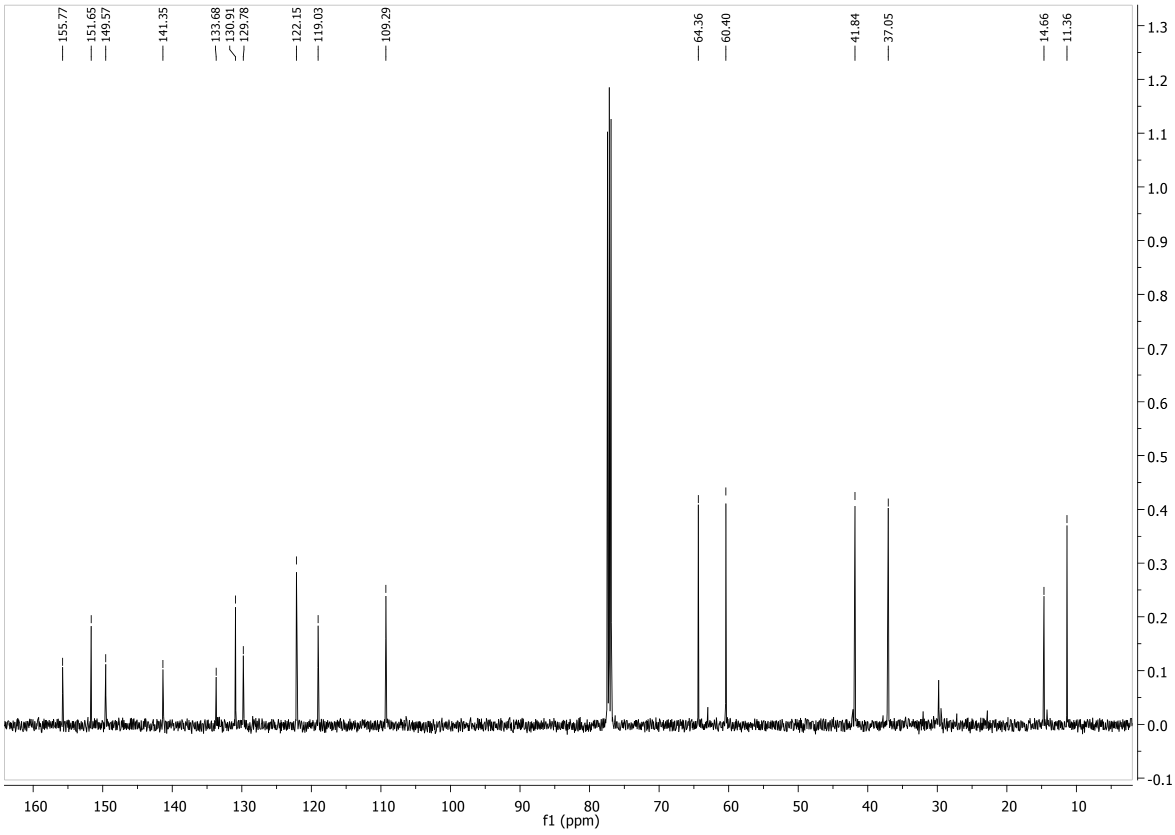

Fluorescence monitoring of the conjugates 4-6 cleavage

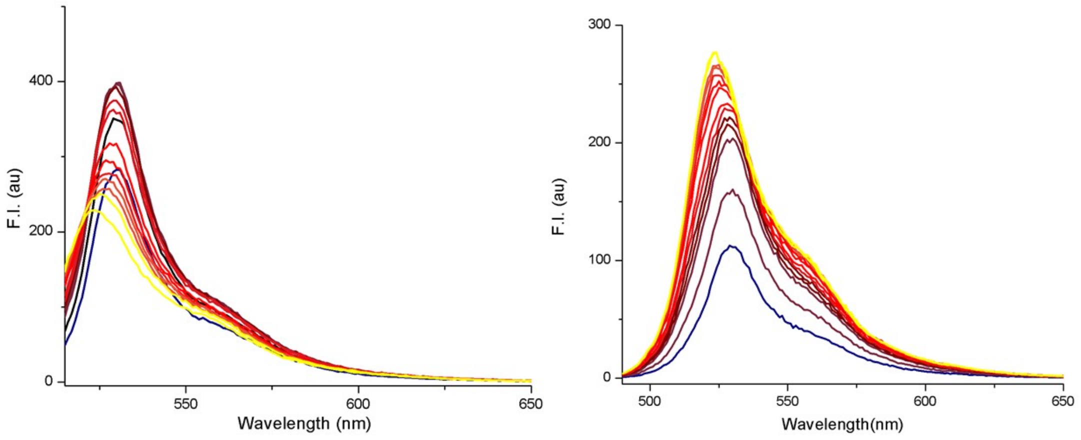

Figure S1. Decreasing of emission intensity of conjugate **4** (left) upon excitation by 510 nm and increasing of emission intensity upon excitation by 480 nm (right) in time during cleavage of conjugate **4** (5μM) by GSH (5mM, DMSO/HEPES 2:1, pH 7.4, 37°C).

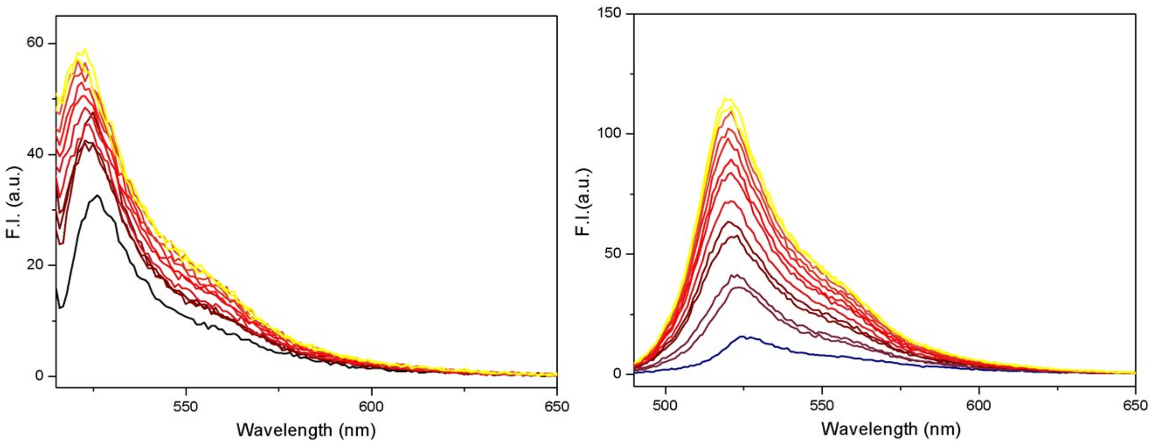

Figure S2. Increasing of emission intensity change of conjugate **5** upon excitation by 510 nm (left) and upon excitation by 480 nm (right) in time during cleavage of conjugate **5** (5μM) by GSH (5mM, 0.1M HEPES Buffer, pH 7.4, 37°C).

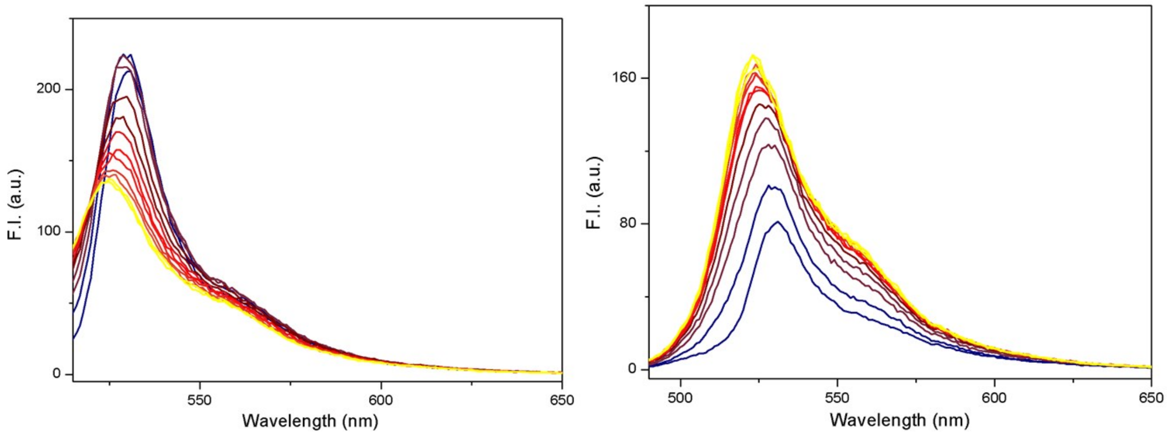

Figure S3. Decreasing of emission intensity of conjugate **5** (left) upon excitation by 510 nm and increasing of emission intensity upon excitation by 480 nm (right) in time during cleavage of conjugate **5** (5μM) by GSH (5mM, DMSO/HEPES 2:1, pH 7.4, 37°C).

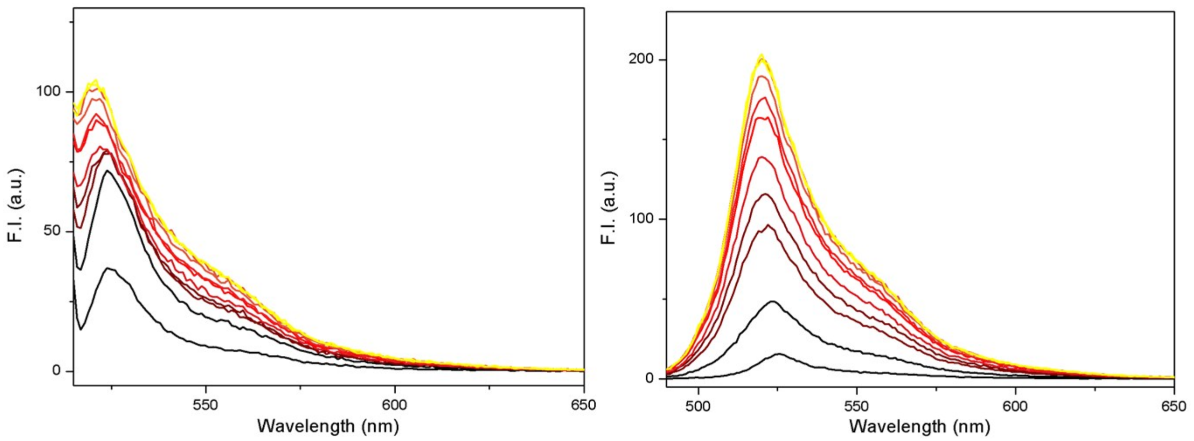

Figure S4. Increasing of emission intensity change of conjugate **6** upon excitation by 510 nm (left) and upon excitation by 480 nm (right) in time during cleavage of conjugate **6** (5μM) by GSH (5mM, 0.1M HEPES Buffer, pH 7.4, 37°C).

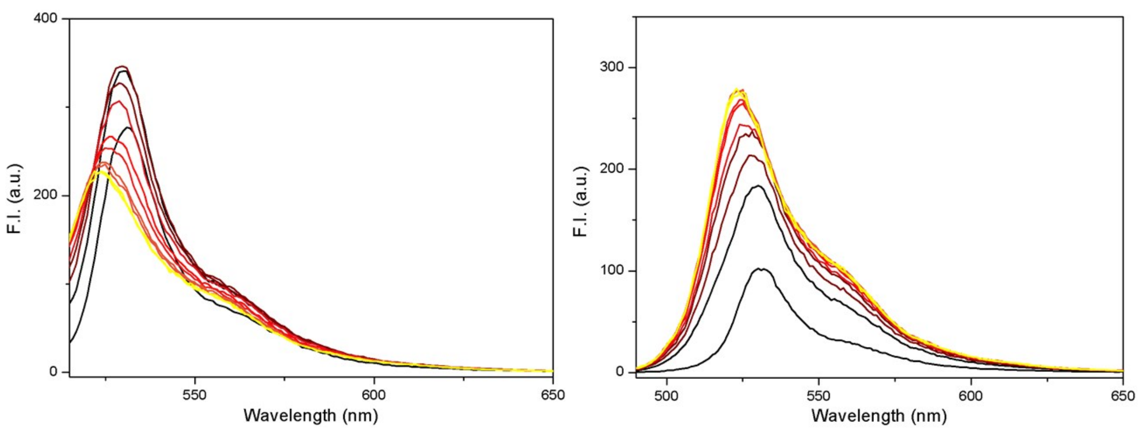

Figure S5. Decreasing of emission intensity of conjugate **6** (left) upon excitation by 510 nm and increasing of emission intensity upon excitation by 480 nm (right) in time during cleavage of conjugate **6** (5μM) by GSH (5mM, DMSO/HEPES 2:1, pH 7.4, 37°C).

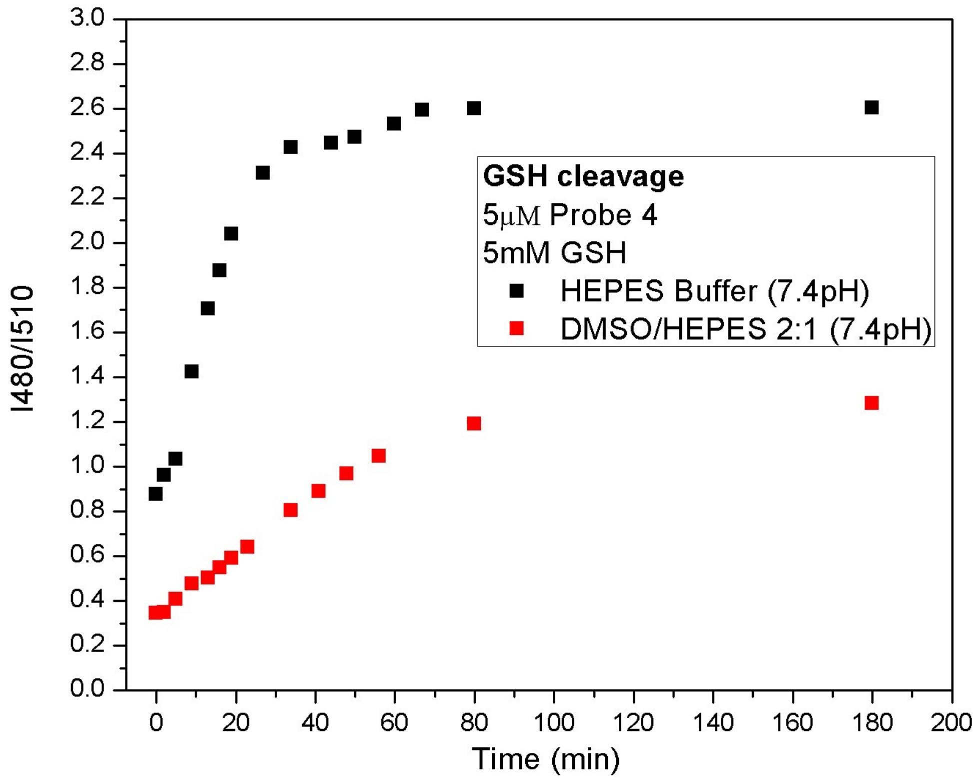

Figure S6. Comparison of the ratio of fluorescence emission at 525nm upon excitation at 480nm and 510nm (I_{480}/I_{510}) for the Probe **4** (5μM) in HEPES Buffer (0.1M, pH 7.4, 37°C) and DMSO/HEPES Buffer 2:1 (0.1M, pH 7.4, 37°C) during GSH (5mM) cleavage.

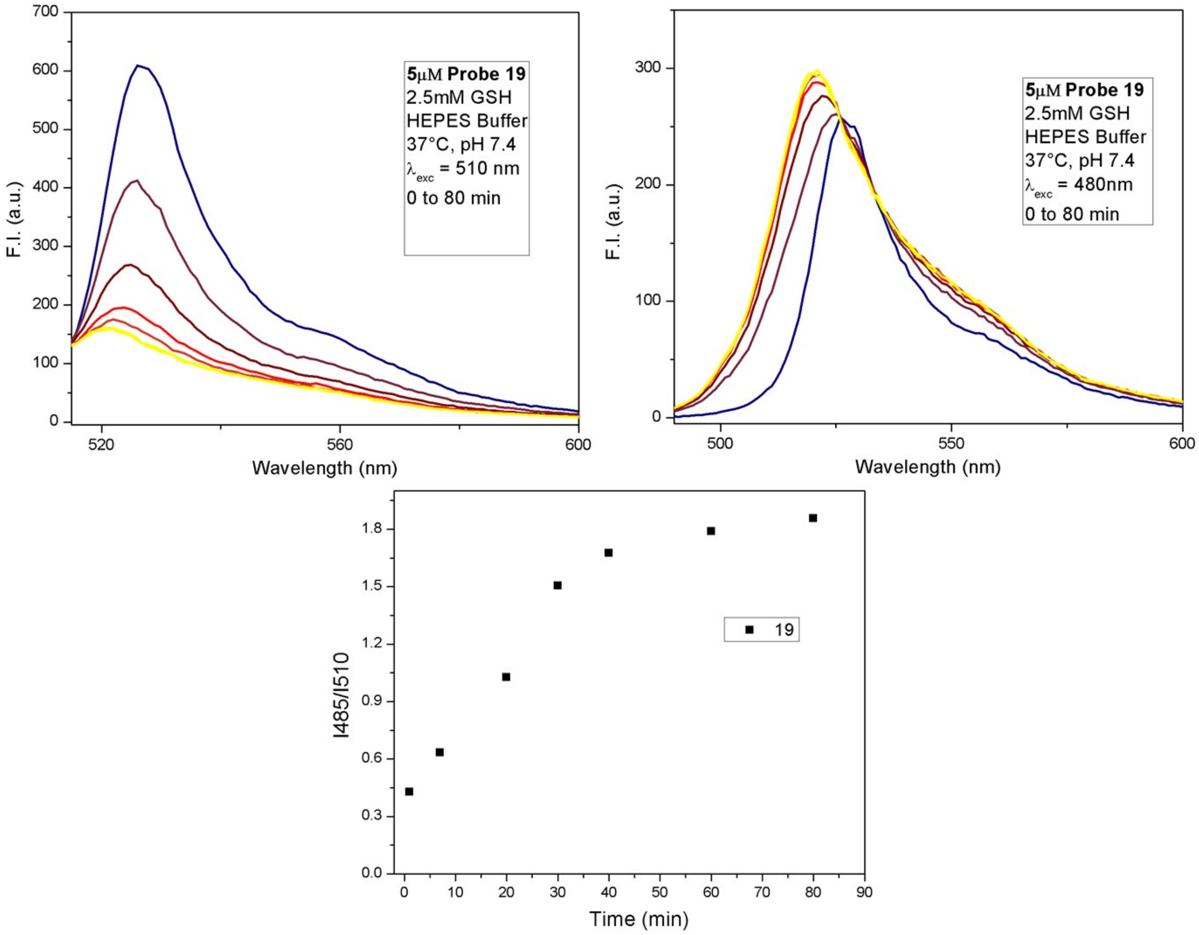

Figure S7. Emission intensity change of conjugate **19** (5μM) upon excitation by 510 nm (left upper) and upon excitation by 480 nm (right upper) in time during cleavage by GSH (2.5mM, 0.1M HEPES Buffer, pH 7.4, 37°C) and representation by the fluorescence ratio (I480/I510) change in time (bottom).

Detection limit

Detection limit (LOD) was calculated as follows:

$$LOD = \frac{3 \cdot \sigma}{s}$$

σ – standard deviation of response

s – slope of the calibration curve

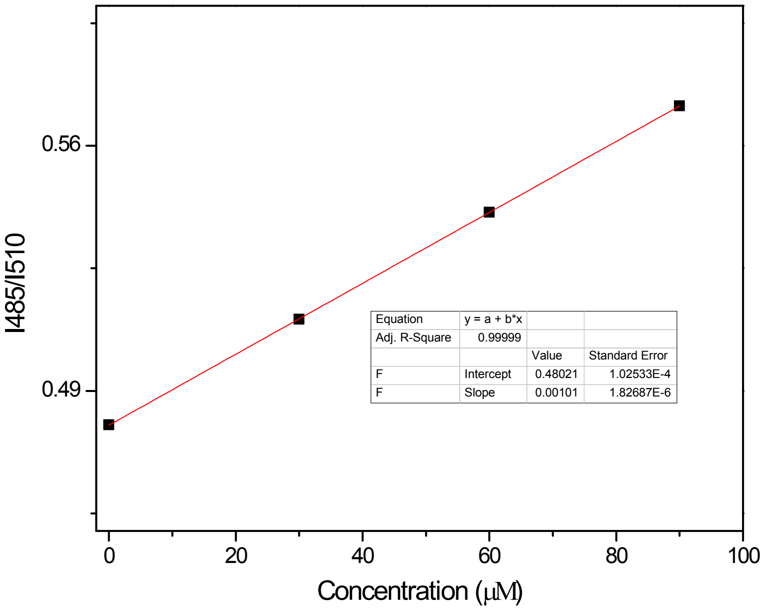

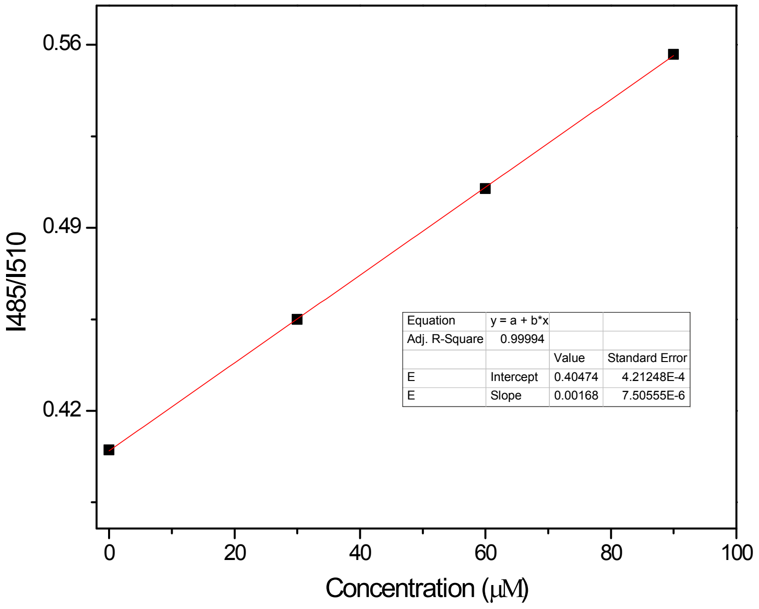

Figure S8. Detection limit determination for compounds **5** (LOD = 305 nM) and **6** (LOD = 752 nM). (GSH concentration 0 μM to 90μM, 0.1M HEPES Buffer, 7.4 pH, 37°C, 2h).

References

(1) Ursuegui, S.; Yougnia, R.; Moutin, S.; Burr, a; Fossey, C.; Cailly, T.; Laayoun, a; Laurent, a; Fabis, F. *Org. Biomol. Chem.* **2015**, *13* (12), 3625–3632.

(2) Soural, M.; Funk, P.; Kvapil, L.; Hradil, P.; Hlaváč, J.; Bertolasi, V. *Arkivoc* **2010**, *2010* (10).

(3) Leen, V.; Leemans, T.; Boens, N.; Dehaen, W. *European J. Org. Chem.* **2011**, No. 23, 4386–4396.

(4) Krajčovičová, S.; Gucký, T.; Hendrychová, D.; Kryštof, V.; Soural, M. *J. Org. Chem.* **2017**, *82* (24), 13530–13541.

(5) Azéma, L.; Lherbet, C.; Baudoin, C.; Blonski, C. *Bioorganic Med. Chem. Lett.* **2006**, *16* (13), 3440–3443.
